# Using game theory to thwart multistage privacy intrusions when sharing data

**DOI:** 10.1126/sciadv.abe9986

**Published:** 2021-12-10

**Authors:** Zhiyu Wan, Yevgeniy Vorobeychik, Weiyi Xia, Yongtai Liu, Myrna Wooders, Jia Guo, Zhijun Yin, Ellen Wright Clayton, Murat Kantarcioglu, Bradley A. Malin

**Affiliations:** 1 Department of Electrical Engineering and Computer Science, Vanderbilt University, Nashville, TN 37212, USA.; 2 Department of Biomedical Informatics, Vanderbilt University Medical Center, Nashville, TN 37203, USA.; 3 Department of Computer Science and Engineering, Washington University in St. Louis, St. Louis, MO 63130, USA.; 4 Department of Economics, Vanderbilt University, Nashville, TN 37235, USA.; 5 Center for Biomedical Ethics and Society, Vanderbilt University Medical Center, Nashville, TN 37203, USA.; 6 School of Law, Vanderbilt University, Nashville, TN 37203, USA.; 7 Department of Pediatrics, Vanderbilt University Medical Center, Nashville, TN 37232, USA.; 8 Department of Computer Science, University of Texas at Dallas, Richardson, TX 75080, USA.; 9 Institute for Quantitative Social Science, Harvard University, Cambridge, MA 02138, USA.; 10 Department of Electrical Engineering and Computer Sciences, University of California, Berkeley, Berkeley, CA 94720, USA.; 11 Department of Biostatistics, Vanderbilt University Medical Center, Nashville, TN 37203, USA.

## Abstract

Person-specific biomedical data are now widely collected, but its sharing raises privacy concerns, specifically about the re-identification of seemingly anonymous records. Formal re-identification risk assessment frameworks can inform decisions about whether and how to share data; current techniques, however, focus on scenarios where the data recipients use only one resource for re-identification purposes. This is a concern because recent attacks show that adversaries can access multiple resources, combining them in a stage-wise manner, to enhance the chance of an attack’s success. In this work, we represent a re-identification game using a two-player Stackelberg game of perfect information, which can be applied to assess risk, and suggest an optimal data sharing strategy based on a privacy-utility tradeoff. We report on experiments with large-scale genomic datasets to show that, using game theoretic models accounting for adversarial capabilities to launch multistage attacks, most data can be effectively shared with low re-identification risk.

## Teaser

Game theory indicates that minimal edits are needed to protect personal data against attacks on anonymity.

## INTRODUCTION

Person-specific biomedical data are now collected on a large scale in a wide range of settings. For instance, in the clinical realm, personal information is routinely stored in electronic health records. The biomedical research domain now supports studies that collect data on a diverse array of participants (*1*). In addition, most recently, the commercial setting has led to a number of ventures where data are collected, such as direct-to-consumer genetic testing companies that collect data from various consumers and build repositories that now cover more than 10% of the U.S. population (*2*). Many believe that sharing these data beyond their initial point of collection is crucial to maximizing the societal value of the data. However, data sharing efforts are often limited by privacy concerns, particularly over the identifiability of data subjects, the individuals to whom the data correspond (*3*).

Genomic data, which are shared in various settings in the United States, provide a clear illustration of the threat of data re-identification and the concern over the possibility. Linking genomic data to explicit identifiers (i.e., re-identification) poses a threat to the anonymity of data subjects. While data managers remove explicit identifiers (e.g., personal names and phone numbers) to adhere to de-identification guidance (*4*–*6*), numerous demonstration attacks have shown that data, and particularly genomic data, can be re-identified through a variety of means (*7*–*10*). Although individuals are incentivized to share their genomic data (*11*–*13*), they usually lack the ability to identify and assess privacy risks properly to make the informed sharing decisions (*12*, *14*).

It is important to recognize that not all re-identification attacks are equally easy to execute and that an oversimplified attack model can lead to an inaccurate measure of risk. Moreover, this inaccuracy is not biased in any particular direction; thus, risk may be underestimated in some cases but overestimated in others. Initially, attacks were based on a single stage (*15*–*17*), where the adversary linked two datasets, one de-identified and one identified, using attributes shared by these datasets (e.g., residual demographics or DNA sequences). More recently, attacks have evolved into multistage forms (*18*–*22*), where each stage reveals another piece of information about a targeted individual.

Here, we introduce the first approach to assessing and strategically mitigating risks by explicitly modeling and quantifying the privacy-utility tradeoff for data subjects in the face of multistage attacks. In doing so, we bridge the gap between more complex models of attack and informed data sharing decisions.

For illustration, we rely on the well-known two-stage attack model of Gymrek *et al.* (*18*), which, to re-identify genomic data, combines surname inference with linkage. Their attack specifically targeted 10 participants in the Center for the Study of Human Polymorphisms (CEPH) family collection, whose genomes were sequenced as part of the 1000 Genomes Project (*23*), by performing surname inference through public genetic genealogy databases made accessible by Ysearch and the Sorenson Molecular Genealogy Foundation (SMGF), now owned by Ancestry.com. In response, in consultation with the local institution managing the CEPH study, the National Institutes of Health moved certain demographics about the participants in the corresponding repository into an access-controlled database (*24*). Although the Ysearch and SMGF databases are no longer accessible to the public (*25*), it is not unreasonable to assume that similar databases may be made publicly accessible in the future.

Various approaches to preventing biomedical data re-identification have been developed from regulatory (*26*–*28*) and technological perspectives (*29*–*36*). However, most of these approaches focus on worst-case scenarios; thus, their impacts on data utility and privacy risk in practice are unclear. For example, the adversary considered in these approaches always attacks without taking into account the attack costs (*7*, *8*), which may lead to an overestimation of the privacy risk. In addition, the parameters in technical protection models [e.g., *k*-anonymity (*37*) or differential privacy (*38*)] are usually set without or before measuring their impacts in specific use cases (*39*, *40*), which may either sacrifice too much data utility or provide insufficient protection. To address this problem, risk assessment and mitigation based on game theoretic models have been introduced (*41*–*43*) (see note S1 for a summary of the current literature).

In our work, we show that a game theoretic model can reveal the optimal sharing strategy to data subjects. The model is such that we can conduct experiments involving protection against a multistage attack using either real-world datasets or large-scale simulated datasets. Our results demonstrate that the game theoretic model can efficiently assess and effectively mitigate privacy risks. The fine-grained sharing strategy recommended by our model can minimize the chance that a data subject will be successfully re-identified while maximizing the data utility and keeping the released dataset useful and the data sharing process fair.

## MATERIALS AND METHODS

**Fig. 1.**
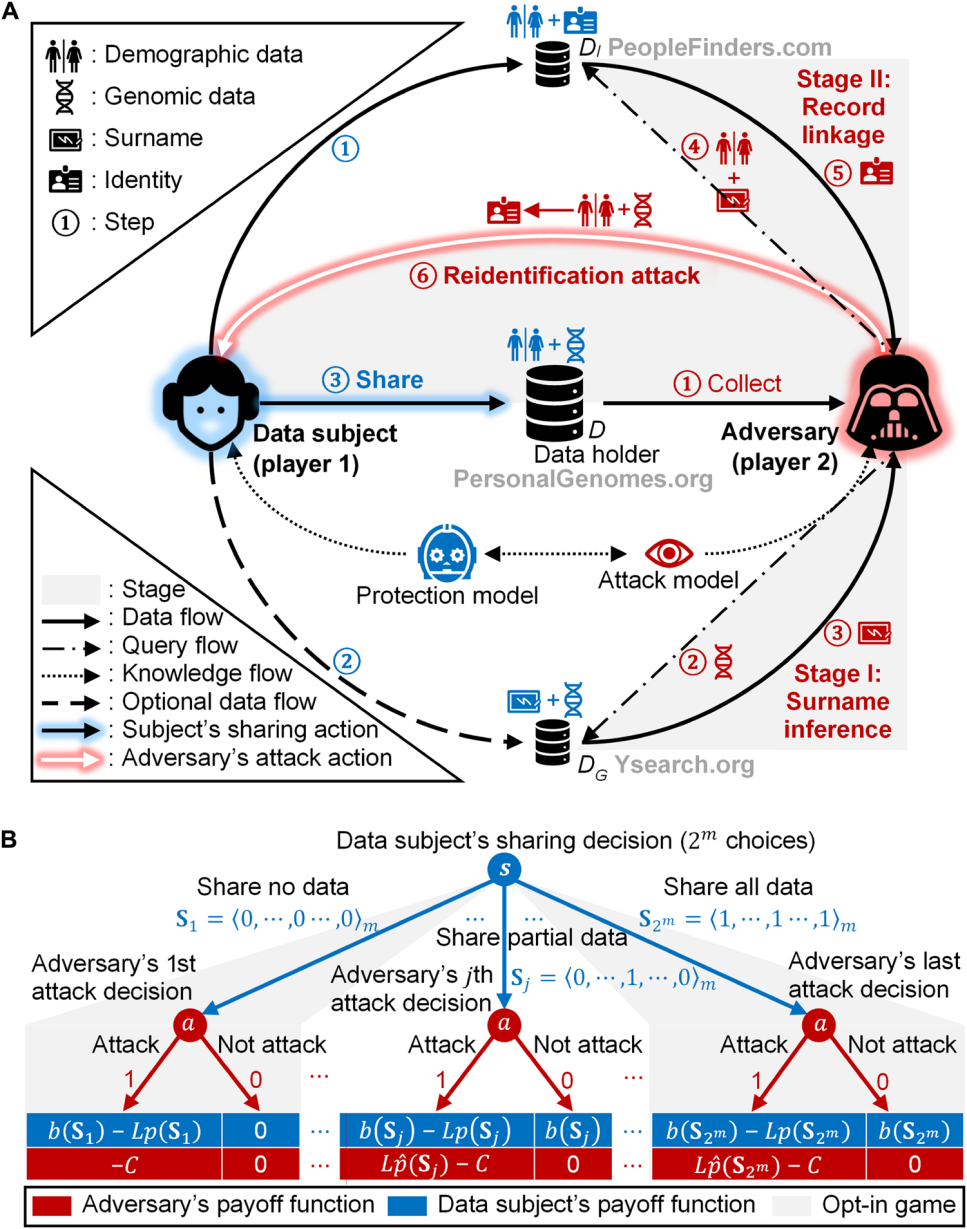
A multistage privacy attack and its game theoretic protection. (**A**) A system-wide perspective of a multistage re-identification attack and its protection. Person-specific data records of a subject are accessible to an adversary through three databases: a targeted genomic database (*D*), a genetic genealogy database (*D_G_*), and a public identified database (*D_I_*). The adversary re-identifies a genomic record by inferring surnames in stage I and linking it to a public record in stage II. The data subject selects a sharing strategy based on a game model only when sharing data in *D*. (**B**) A masking game represented in the extensive form. In a masking game, the data subject moves first, and the adversary moves next. Each terminal node is associated with both players’ payoffs. **S***j* is an *m*-dimensional vector of 0 and 1 values, representing the *j*th concrete action of the data subject. More denotation details are in the main text. The opt-in game is a special variation of the masking game in which the data subject only has those two strategies.

We investigate a situation in which a data subject chooses how much of the subject’s genomic data to share in a public repository, such as the 1000 Genomes Project (*23*), OpenSNP (*11*), or the Personal Genome Project (*44*). For example, the subject may share the entire sequence, a subset of single-nucleotide polymorphisms (SNPs), a subset of short tandem repeats (STRs), or nothing at all. Our goal is to determine the subject’s optimal data sharing decision, balancing the monetary benefit (or some benefit that can be translated into monetary terms) of data sharing and the re-identification risk. Re-identification risk, in turn, arises from two sources: first, an adversary, or anyone who has the incentive and means to attempt to determine the identities of subjects in anonymized shared datasets; and, second, other data about the subject that are already available to the public, possibly at some cost. In Fig. 1A, we illustrate this setting in the context of a particular re-identification attack introduced by Gymrek *et al.* (*18*), which we refer to as “the Gymrek attack,” in which the adversary first used the shared data together with Ysearch (another public dataset) to infer an individual’s surname and subsequently used this additional information to perform a record linkage attack using a third dataset (e.g., PeopleFinders).

We initially assume that the adversary and the data subject have the same beliefs over the probability that an attack will be successful. This allows us to formalize the encounter just described as a Stackelberg, or leader-follower, game (*45*), in which the subject acts as a leader, choosing how much of their genomic data to share, and the adversary is the follower who obtains the shared data and then decides whether to execute an attack (see Fig. 1B). We then take account of the fact that the adversary, since they do not know the values of the masked attributes, can only estimate the probability of the success of an attack, while the data subject has better information.

Note that the primary focus of the encounter is the genomic dataset that results from the explicit data sharing decision by the subject; however, two additional peripheral datasets (e.g., Ysearch and PeopleFinders) play a role in the re-identification attack and are viewed as a part of the overall environment by both the subject and the adversary. Note that neither has any impact on the availability of these datasets.

We model the subject’s decision about what portion of the data to share by a vector $s=<s_{1},\cdots ,s_{j},\cdots ,s_{m}>\in B^{m}$ of 0 and 1 values in which *m* is the number of attributes in the record, *s_j_* = 0 if the *j*th attribute is masked, and *s_j_* = 1 if the *j*th attribute is shared. Thus, if the subject shares a collection of STRs indexed by *j*, then an entry of 0 in this vector implies that the subject does not share the corresponding STR, whereas an entry of 1 means that this STR is shared. Since the sharing strategy **s** of the subject involves masking (outcome-equivalent to redacting) a subset of STRs, we refer to the subject’s strategies as masking strategies and the game as a masking game.

The adversary observes the subject’s released set of STRs, encoded by their strategy vector **s**, and decides whether to attack, which we encode as a binary choice *a* ∈ {0,1}, where *a* = 1 means that the adversary decides to attack and *a* = 0 means that they do not attack. If the adversary chooses to attack, then the actual execution of the attack follows a two-stage process, such as the one demonstrated by the Gymrek attack. Specifically, in stage I, the adversary infers the target’s surname by comparing the subject’s genomic record in database *D* with genomic records retrieved from database *D_G_*. In stage II, the adversary tries to re-identify the subject by linking the subject’s genomic record in database *D* with an identified record in database *D_I_* on a set of demographic attributes (namely, year of birth, gender, and state of residence) and the inferred surname. In general, the attack can have more than two stages, in which each stage infers new information, based on an additional dataset, which can be used in subsequent stages.

A crucial element of our game theoretic model of the interaction between the data subject and the adversary is the sequence of decisions. The subject (the leader) makes a decision first, choosing a masking strategy **s**. The adversary (the follower) then observes the data and, consequently, what has been masked and chooses whether to attack depending on the masking strategy **s**; that is, the adversary’s strategy is a function of **s**.

To formalize the consequences of the decisions by both the data subject and the adversary, we now introduce additional notation and assumptions. First, let *b*(**s**) capture the monetary benefit to the subject that results from sharing data using a masking strategy **s**. This benefit function is known to both the subject and the adversary. We assume that the benefit function is nondecreasing in the amount of data shared (see note S2 for a concrete example). Second, if a record is successfully re-identified by the adversary, then we assume that the subject incurs a loss of *L*, which is also the amount gained by the adversary.

The probability that an attack will be successful depends on the masking strategy used by the data subject. Since the data subject knows the values of the masked attributes, the data subject will have a better understanding of the probability of success of an attack, the re-identification risk probability, than the adversary. As noted above, in practice, the adversary can only estimate the likelihood of an attack’s success because the adversary, in contrast to the subject, does not know the values of the masked attributes. Initially, to enable us to present the game situation as a standard Stackelberg game, we assume that both the data subject and the adversary assign the same probability of success to each strategy **s**. Let *p*(**s**) denote this probability. Taking *p*(**s**) as given, both the data subject and the adversary can maximize their expected payoffs. Then, as further described below, taking expected payoffs as the actual payoffs, we have a Stackelberg game of perfect information and can study its equilibria.

With the notation in hand, we can now define both players’ expected payoff (utility) functions. The payoff of the data subject (subscript *d* for defender) is

$$v_{d}(s,a)=b(s)-\mathrm{Lp}(s)a$$ (1)

The payoff of the adversary is

$$v_{a}(s,a)=(\mathrm{Lp}(s)-C)a$$ (2)

where *C* is the cost paid by the adversary to execute the attack. Note that the adversary’s payoff is 0 if he or she chooses not to attack (i.e., *a* = 0), no matter what the subject does. Note also that we can generalize our model to other data sharing scenarios by redefining the benefit function *b*(**s**) and the re-identification risk probability function *p*(**s**) as appropriate.

With the specification of strategy sets and terminal payoffs, we have defined a Stackelberg leader-follower game. Our goal is to identify the subject’s strategy **s** (with some tie-breaking rule if needed) that is a part of a strong Stackelberg equilibrium (SSE; a special case of a subgame perfect Nash equilibrium) (*46*). Let ϕ(**s**) = {*a*∣(*Lp*(**s**) − *C*)*a* ≥ (*Lp*(**s**) − *C*)(1 − *a*)}; the function ϕ(**s**) is the set of best responses of the adversary to the subject’s strategy **s**. Then, SSE corresponds to the following optimization problem for the subject

$$\underset{s,a\in \phi (s)}{\text{max}}b(s)-\mathrm{Lp}(s)a$$ (3)

To solve the game, the subject can find the best (i.e., SSE) strategy (s^*^) using backward induction, where the subject first computes the best responses ϕ(**s**) of the adversary for all possible strategies **s**, and then choose the best strategy for the subject (e.g., through exhaustive search).

The key practical limitation of the discussion above is that, in practice, since the data subject has their complete dataset (which the adversary does not), they can compute the re-identification probability *p*(**s**). This is not, however, the case for the adversary. We capture this asymmetry by allowing the adversary to use an estimated re-identification probability, which we denote by $\hat{p}(s)$, in computing their payoff and thus their best response function. The data subject can also use $\hat{p}(s)$ in computing the best response function of the adversary, but then the data subject can use the true re-identification risk probability in calculating their optimal choice.

More precisely, both the data subject and the adversary can calculate the best response function of the ϕ(**s**) using the re-identification risk probability estimate $\hat{p}(s)$, say $\hat{\phi }(s)=\{a|(L\hat{p}(s)-C)a\geq (L\hat{p}(s)-C)(1-a)\}$. Then, the data subject, knowing when the adversary will attack, can calculate their optimal strategy,$\underset{s,a\in \hat{\phi }(s)}{\text{max}}b(s)-\mathrm{Lp}(s)a$, using the actual probability of success *p*(**s**). Note that the special feature of a Stackelberg game that the leader makes only one decision enables us to use the two possibly different re-identification risk probability functions.

We now describe precisely how we compute both the true (for the data subject) and estimated (for the adversary) re-identification probabilities. Specifically, in a two-stage attack, given **s**, *p*_1_(**s**) denotes the probability of success of stage I of the attack, *p*_2_(**s**) denotes the probability of success of stage II of the attack given that stage I succeeds, ${\hat{p}}_{1}(s)$ denotes the adversary’s estimation on the probability of stage I’s success, and *p*′(**s**) denotes the probability of stage II’s success given that stage I is omitted (i.e., no surname inference performed). Thus, we can represent the true and estimated probabilities of a two-stage re-identification attack’s success as shown in Eq. 4

$$\begin{array}{c} p(s)=p_{1}(s)p_{2}(s)a\prime (s)+p\prime (s)(1-a\prime (s))\\ \hat{p}(s)={\hat{p}}_{1}(s)p_{2}(s)a\prime (s)+p\prime (s)(1-a\prime (s))\\ a\prime (s)=\{\begin{array}{cc} 1, & {\hat{p}}_{1}(s)p_{2}(s)>p\prime (s)\\ 0, & {\hat{p}}_{1}(s)p_{2}(s)\leq p\prime (s) \end{array} \end{array}$$ (4)

where *a*′(**s**) is 1 if stage I is better executed and is 0 if stage I is better omitted. The specific settings of *p*_1_(**s**), *p*_2_(**s**), ${\hat{p}}_{1}(s)$, and *p*′(**s**) depend on the attack model and datasets used in an attack.

On the basis of Eqs. 3 and 4, the optimization problem can be represented specifically for a two-stage attack, if *a*′(**s**) = 1, as shown in Eq. 5

$$\underset{s,a\in \hat{\phi }(s)}{\text{max}}b(s)-Lp_{1}(s)p_{2}(s)a$$ (5)

where $\hat{\phi }(s)=\{a|(L{\hat{p}}_{1}(s)p_{2}(s)-C)a\geq (L{\hat{p}}_{1}(s)p_{2}(s)-C)(1-a)\}$. Further details about the derivations of the game model and the ways to set parameters are described in note S3. Note that all parameters can be reasonably set according to a use case. Specifically, they can be tailored to a specific dataset, the attack model, or the valuation provided by a subject. In addition, as our results show, extensive sensitivity and robustness analyses (i.e., stress tests) can help verify the sensitivity and robustness of the parameter settings against the uncertainty in an environment or a subject’s knowledge.

We note that in some cases, a subject can only choose from two options: They can either opt in and share all required information or opt out and share nothing. We can view this scenario as a variation of the game we defined above where the strategy of the subject is restricted to two options: share everything (**s** = <1, ⋯,1>) and share nothing (**s** = <0, ⋯,0>). We refer to this game as the opt-in game. This game’s model can be represented as a part of the masking game model, as shown in Fig. 1B (shaded in gray). We can restrict the strategy space for the defender (i.e., the data subject) to any subset of the entire set of strategies **s**.

Solving a game via exhaustive search may be time consuming, especially when a complex attack model is considered. Algorithms that could be used to accelerate the search process (e.g., the greedy algorithm and the pruning technique) are described in note S4. The implemented game solver with datasets included can be accessed from https://github.com/zhywan/msrigs (archived at https://doi.org/10.5281/zenodo.5543369). The notation used throughout the main text and all supplements is summarized in table S1.

## RESULTS

### Experimental design

To demonstrate our model and evaluate the effectiveness of our methods, we conducted two sets of experiments based on genomic datasets. In one set of experiments, we used real datasets composed of STRs on the Y chromosome (Y-STRs) derived from Craig Venter’s genomic record and the Ysearch dataset with 156,761 records and 100 Y-STRs, as they were used by Gymrek *et al.* (*18*). To protect the privacy of the corresponding subjects and enable replications of our investigation, we sanitized (i.e., modified for privacy protection) the original datasets without affecting the demonstration (see note S5 for details about the data sanitization process). In the other set of experiments, to evaluate the effectiveness of our methods in a larger and more controllable environment and to facilitate replications of our investigation without privacy concerns, we simulated a genetic genealogical population of 600,000 individuals (see note S6 for details about the data preparation process), from which multiple datasets were sampled. To further evaluate our methods’ effectiveness under various circumstances and uncertainties, we conducted a sensitivity analysis for eight parameters and three experimental settings and conducted a robustness analysis for three parameters. The default values for parameters for the experiments are provided in table S1.

To measure the effectiveness, we calculated the average payoff for a pool of *n* data subjects, whose records may be shared in genomic dataset *D*, as: $\bar{V}=\sum_{i=1}^{n}V_{i}$, where *V_i_* represents the *i*th subject’s optimal payoff. We further calculated the average data utility ($\bar{U}=\sum_{i=1}^{n}U_{i}$) and the average privacy ($\bar{P}=\sum_{i=1}^{n}P_{i}$) of those subjects to show how the game model can balance these two factors. That is, we can calculate effectiveness measures (namely, the average payoff, the average data utility, and the average privacy) after obtaining the optimal payoff, the corresponding data utility, and the corresponding privacy for each subject, given the best strategy ($s_{i}^{*}$), the data record, and parameter settings. For simplicity, we assumed that those subjects use the same parameter settings. More specifically, the *i*th subject’s data utility (*U_i_*) is defined as the benefit of sharing divided by the maximal benefit of sharing all data as follows: $U_{i}=b(s_{i}^{*})/B$. In addition, the *i*th subject’s privacy (*P_i_*) is defined as one minus the privacy risk (i.e., the probability to be successfully attacked) as $P_{i}=1-p(s_{i}^{*})a_{i}^{*}$, in which $a_{i}^{*}\in \phi (s_{i}^{*})$ is the adversary’s best response, and $p(s_{i}^{*})$ is the probability of success of an attack given $s_{i}^{*}$. Notably, the *i*th subject’s optimal payoff (*V_i_*) can be represented as a linear combination of the corresponding data utility and privacy: $V_{i}=v_{d}(s_{i}^{*},a_{i}^{*})=BU_{i}-L(1-P_{i})=BU_{i}+LP_{i}-L$, in which *L* is the loss from being re-identified. As the primary measure of effectiveness, the average payoff of those subjects is positively correlated with the metrics for utility and privacy.

Our simulated population was generated with 20 attributes, including ID, surname, year of birth, U.S. state of residence, and 16 genomic attributes. On the basis of the simulated population, we ran experiments to find the best sharing strategy for each subject in a targeted dataset in four game scenarios and four baseline scenarios. In each run of the experiments, from the simulated population, we randomly selected 1000 records for the targeted genomic dataset *D*, 20,000 records for the identified dataset *D_I_*, and 20,000 records for the genetic genealogy dataset *D_G_*. On the basis of these datasets, we compared eight scenarios: (i) no protection, (ii) demographics only, (iii) random opt-in, (iv) random masking, (v) opt-in game, (vi) masking game, (vii) no-attack masking game, and (viii) one-stage masking game. In all scenarios, the adversary aims to re-identify all records in dataset *D*, but he or she makes rational decisions according to their estimated payoff.

The following provides a brief description of how the recommended strategy is selected in each scenario. In the no-protection scenario, each subject in dataset *D* shares all attributes in their data record. In the demographics-only scenario, each subject in dataset *D* shares demographic attributes only. In the random opt-in scenario, each subject in dataset *D* randomly decides to share the entire data record with a certain probability according to a practical setting. In the random masking scenario, each subject in dataset *D* randomly decides to share each attribute in their data record with a fixed probability. In game scenarios, each subject in dataset *D* makes rational decisions by playing the masking game or one of its variations. In the masking game, the subject can mask a portion of data before sharing. However, in the opt-in game, the subject can only decide to opt in to share the entire data record or to opt out. In the no-attack masking game, we assume that the subject chooses no strategy that will make the adversary attack. Whereas in the one-stage masking game, we assume that dataset *D_G_* is not available to the adversary, and thus, the attack has only one stage. Further details about the datasets used in the experiments are summarized in Table 1, while the detailed settings associated with each scenario are provided in the final section of note S3.

**Table 1.** A summary of the datasets used in the experiments.

		**Set of experiments**	
		**Large-scale evaluation**	**Real-world demonstration**
**Targeted genomic dataset (*D*** **)**	**Dataset**	Simulated genomic dataset	Craig Venter’s data
	**Attributes**	Year of birth, U.S. state, 16 STRs	Year of birth, U.S. state, 50 STRs
	**Records**	1000	1
**Genetic genealogy dataset** **(*D_G_*)**	**Dataset**	Simulated genetic genealogy dataset	Ysearch
	**Attributes**	Surname, 16 STRs	Surname, 50 STRs
	**Records**	20,000	58,218
**Public identified dataset** **(*D_I_*)**	**Dataset**	Simulated demographic dataset	PeopleFinders
	**Attributes**	ID, name, year of birth, U.S. state	Name, age, U.S. state
	**Records**	20,000	~250 million

From the perspective of a data holder (e.g., the controller of a public data repository) who cares about the data quality and the fairness of the collected dataset from a pool of subjects, we defined two types of measures (namely, usefulness and fairness) to evaluate the downstream effects of the adopted data protection/sharing strategy in each scenario. The usefulness of a data sharing solution is based on the distance between the distributions of the shared and the unprotected data (see note S7 for details). The fairness of a data sharing solution (e.g., fairness with respect to usefulness or fairness with respect to privacy) is based on the Gini coefficient of the specific measures corresponding to each demographic group (see note S8 for details).

### Experiments based on a large-scale simulated population

**Fig. 2.**
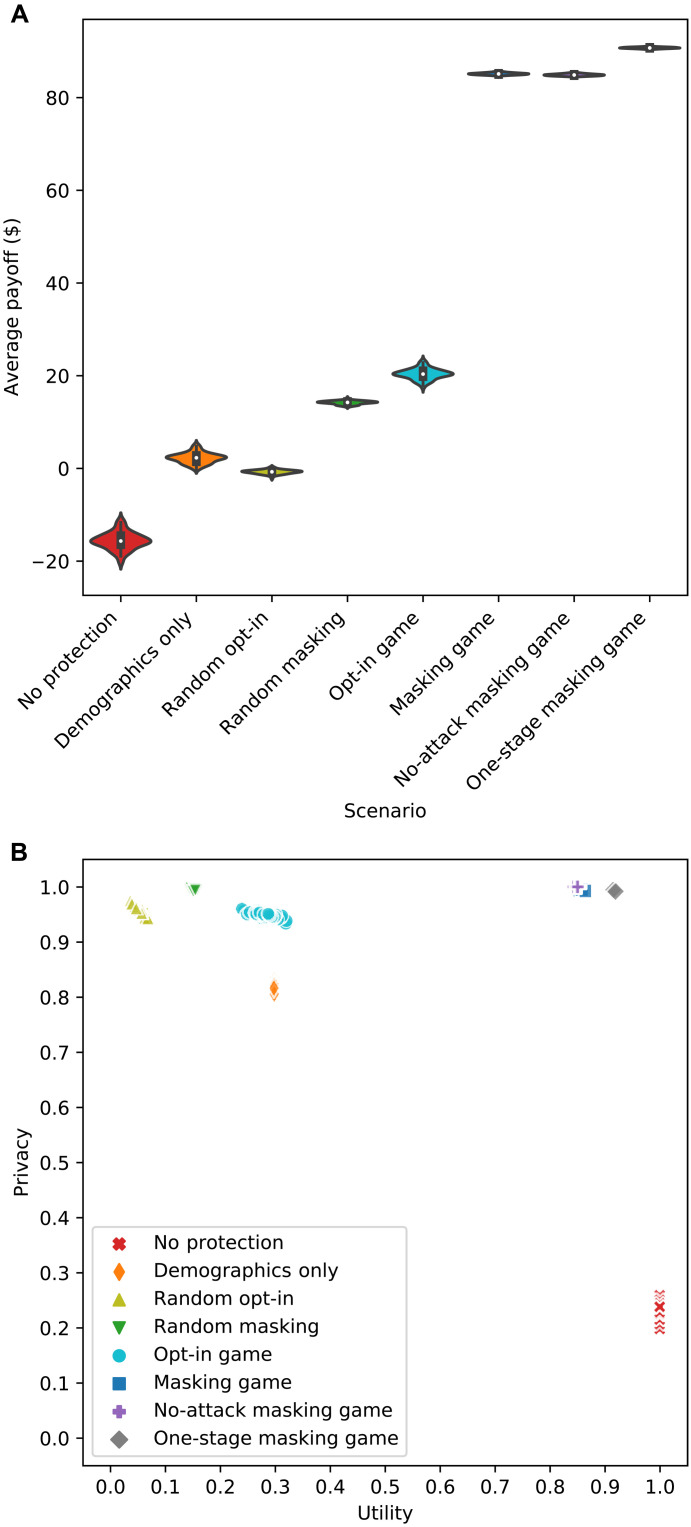
Effectiveness measures of the protection in eight scenarios against a multistage re-identification attack targeting data from 1000 subjects. (**A**) Violin plot of eight distributions of the data subjects’ average payoffs, where each distribution corresponds to one scenario. The violin plot (depicted using Seaborn) combines boxplot and kernel density estimate for showing the distribution of data subjects’ payoffs in each scenario. A Gaussian kernel is used with default parameter settings. (**B**) Scatterplot of data subjects’ average privacy metrics and average utility metrics, where each mark corresponds to one scenario and 1 run (of 100 runs).

We ran the experiment 100 times using the backward induction algorithm with pruning and depicted the results in Fig. 2. Figure 2A shows a violin plot of the distributions of the data subjects’ average payoffs in all eight scenarios, and Fig. 2B shows a scatterplot of the data subjects’ average privacy and utility in each scenario.

Several observations are worth highlighting. First, in Fig. 2A, it can be seen that the subjects’ average payoff is lowest in the no-protection scenario and highest in the masking game. Second, the subjects’ average payoff is improved substantially in the masking game, compared to that in the opt-in game. This observation illustrates one of the essential advantages of providing some degree of granular choice in the data sharing process. Third, the masking game works better when the adversary uses fewer data resources and, thus, keeps fewer stages in the attack. Last, a universal strategy, whether it is sharing all data or sharing demographics only, or a randomized strategy brings a negative or negligible average payoff to the subjects.

In Fig. 2B, the results representing the opt-in game are all in the plot’s top left corner, which implies that the subjects’ strategies in this scenario tend to achieve high privacy but low utility. By contrast, the results representing the no-protection scenario are all in the plot’s lower right corner, which implies that the subjects’ strategies in this scenario tend to achieve high utility but low privacy. Only the results representing the masking game (and two of its variations) are in the plot’s top right corner, where the subjects’ strategies achieve relatively high utility and high privacy at the same time. Notably, the subjects’ strategies in the no-attack masking game guarantee full privacy protection with a substantial amount of shared data. In addition, a slightly higher level of data utility is achieved when the attack has only one stage. The subjects’ strategies in the remainder of the scenarios, however, are all worse than those in game scenarios. Specifically, compared to what the masking game does, the random opt-in scenario and the random masking scenario bring the subjects a similar privacy level but a much lower level of data utility. By contrast, compared to the opt-in game, the demographics-only scenario brings the subjects a similar utility level but a lower privacy level. However, compared to the outcome for the no-protection scenario, the demographics-only scenario always provides the subjects with much higher privacy level, which highlights the power of the surname inference stage in the Gymrek attack. Notably, with the game theoretic protection, the power of the surname inference stage can be reduced to the minimum, as shown by the difference between the masking game and its one-stage variation.

The effectiveness measures in the first run of the experiments are reported in Table 2 with additional statistics (i.e., SDs) of payoffs, data utilities, and privacy measures for the group of subjects. It can be seen that the subjects obtain a higher average payoff and higher average privacy when they make decisions based on game models. In addition, the subjects’ average payoff in the masking game is three times greater than the one in the opt-in game. The masking game also achieves higher average utility and higher average privacy than the opt-in game does for the subjects. More specifically, about 30% of data are shared, and about 6% of the subjects are expected to be re-identified in the opt-in game. By contrast, about 86% of data are shared, and fewer than 1% of the subjects are expected to be re-identified in the masking game. Compared to all baseline scenarios and the opt-in game, the masking game achieves higher average payoff and average privacy with lower SDs for the subjects. By contrast, although the opt-in game generates a positive average payoff, a few subjects’ payoffs are still negative because the corresponding SD is higher than the average. All baseline scenarios provide the subjects with a negative or relatively low average payoff. Among all scenarios, the no-protection scenario exhibits the highest SD (or variation) among subjects in terms of payoff and privacy, while the opt-in game exhibits the highest SD (or variation) among subjects in terms of data utility.

**Table 2.** Effectiveness measures of protection scenarios against a multistage re-identification attack in the first run of the experiments. Scenarios include (1) no protection, (2) demographics only, (3) random opt-in, (4) random masking, (5) opt-in game, (6) masking game, (7) no-attack masking game, and (8) one-stage masking game.

**Notation**	**Description**	**Scenario**							
		**Baseline**				**Game**			
		**1**	**2**	**3**	**4**	**5**	**6**	**7**	**8**
$\bar{V}$	Average payoff of data subjects	−$13.31	−$1.92	−$0.62	$13.83	$21.64	$85.22	$84.94	$90.63
σ*_V_*	SD of data subjects’ payoffs	$57.65	$33.38	$11.96	$12.50	$35.85	$7.23	$7.22	$5.78
$\bar{U}$	Average data utility of subjects	1	0.298	0.043	0.1472	0.301	0.8599	0.8494	0.9153
σ*_U_*	SD of subjects’ data utility	0	0	0.2029	0.1032	0.4587	0.0746	0.0722	0.0623
$\bar{P}$	Average privacy of data subjects	0.2446	0.8141	0.9672	0.9941	0.9436	0.9948	1	0.994
σ*_P_*	SD of data subjects’ privacy	0.3844	0.2225	0.173	0.0637	0.1271	0.0194	0	0.0209

Table 2 further demonstrates the additional stage’s contribution to the two-stage re-identification attack in terms of the chance of success. Note that in the demographics-only scenario, the adversary can only perform a one-stage attack. It can be seen that, in the no-protection scenario, about 76% of the subjects are expected to be re-identified, whereas, in the demographics-only scenario, about 19% of the subjects are expected to be re-identified. That is, the additional stage raised the success rate of the attack by a factor of 4. Nevertheless, such a high re-identification rate implies that most targeted subjects are uniquely identifiable from the identified dataset *D_I_*. The additional stage’s contribution is further demonstrated by comparing two masking games with a different number of attack stages. It can be observed that the game theoretic protection against the one-stage attack brings better averages and SDs with respect to both payoff and utility. Notably, the additional stage induces the subjects to yield only about 6% of their data utility to secure a substantially lower privacy risk, 13% fewer re-identified subjects.

**Fig. 3.**
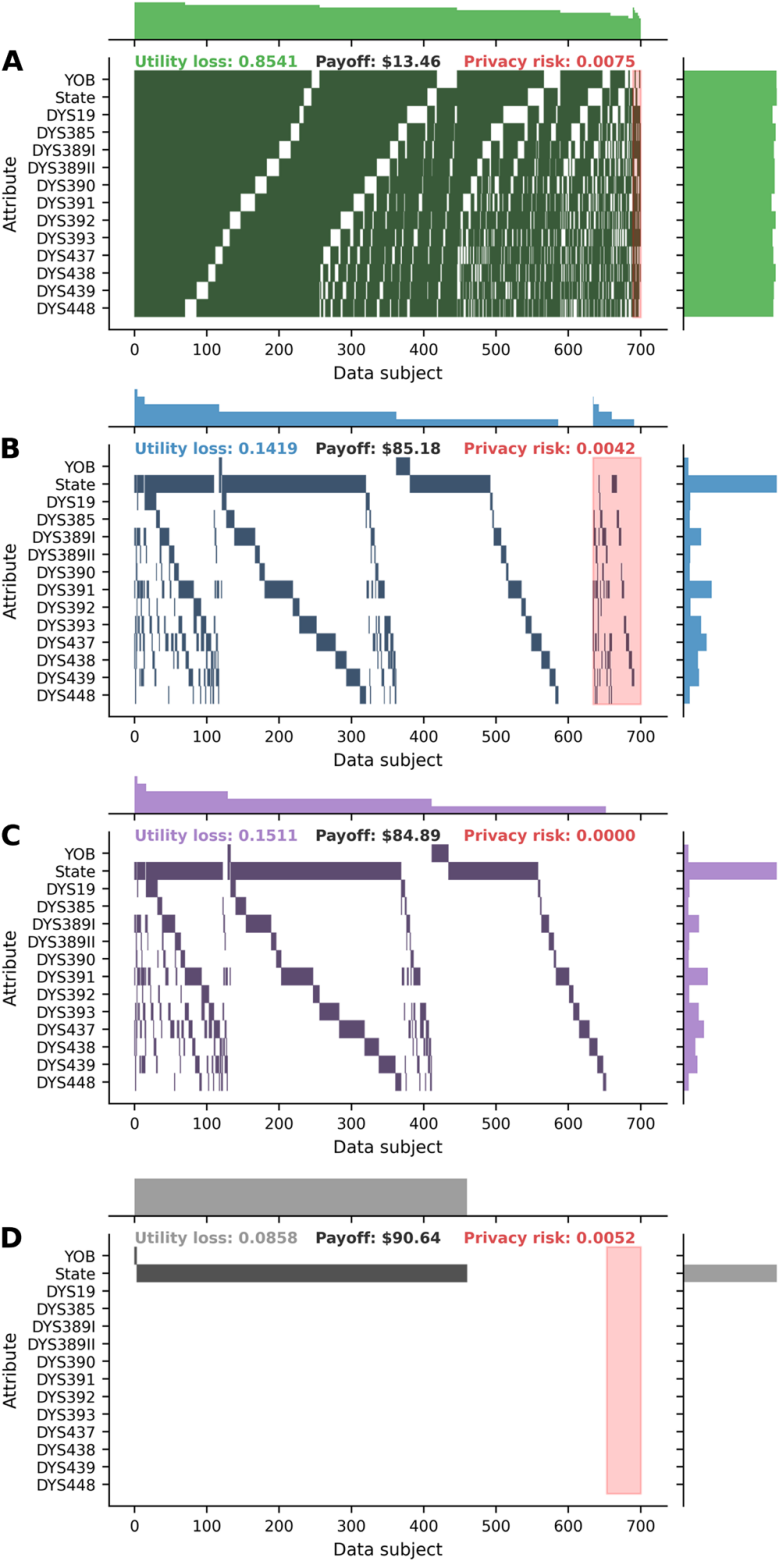
Best strategies for the first 700 data subjects in the first run of experiments in the random masking scenario and three masking game scenarios. (**A**) Random masking scenario. (**B**) Masking game. (**C**) No-attack masking game. (**D**) One-stage masking game. Each nonwhite block indicates that a data subject masks a specific attribute. Each row represents an attribute, and each column represents a data subject. The distribution for data subjects (attributes) is summarized in the histogram on the top (on the right) with the number of bins equal to the number of data subjects (attributes). Data subjects are split into two groups: those on the left that will not be attacked and those on the right that will be attacked (shaded in red). Columns (or data subjects) within each group are sorted by the number of masked attributes in descending order. Rows (or attributes) are sorted by the order of attributes in the dataset. For each scenario, the average payoff, utility loss, and privacy risk are presented at the top center, top left corner, and top right corner, respectively. For each data subject, utility loss is defined as one minus data utility, and privacy risk is defined as one minus privacy. YOB, year of birth; DYS, DNA Y-chromosome segment.

Figure 3 shows the best strategies for the first 700 data subjects in the first run of the experiments in the masking scenarios. In the masking game, only a small portion of data is masked for most subjects. Notably, the no-attack masking game brings full privacy protection, and the one-stage masking game brings better average payoff, to those subjects. By contrast, in the random masking scenario, the subjects’ average utility loss is substantially higher, although the average privacy risk is almost the same as those in the game scenarios.

Table 3 summarizes the computational efficiency of our implementation for each scenario, in terms of average running time across 100 runs. We solved each masking game twice using the backward induction algorithm and the greedy algorithm with pruning. The efficiency of computing the resulting strategies for all 1000 data subjects in each scenario is evaluated on a machine with a six-core 64-bit central processing unit clocked at 4.19 GHz and a 32-gigabyte random-access memory clocked at 2400 MHz. It can be seen that the implementation of the opt-in game scenario runs the fastest, with a speed that is almost the same as the fastest baseline scenario runs. In addition, the masking game solved using the greedy algorithm with pruning runs at a rate that is more than two orders of magnitude faster than the masking game solved using the backward induction algorithm with pruning while achieving approximately the same results (see note S4 for details). However, it runs about one order of magnitude slower than all of the baseline scenarios and the opt-in game scenario.

**Table 3.** Computational efficiency of protection scenarios against a multistage re-identification attack averaged across 100 runs of the experiments. Scenarios include (1) no protection, (2) demographics only, (3) random opt-in, (4) random masking, (5) opt-in game, (6) masking game, (7) no-attack masking game, and (8) one-stage masking game. BIAP, backward induction algorithm with pruning; GAP, greedy algorithm with pruning.

	**Scenario**										
	**Baseline**				**Game**						
	**1**	**2**	**3**	**4**	**5**	**6**		**7**		**8**	
						**BIAP**	**GAP**	**BIAP**	**GAP**	**BIAP**	**GAP**
**Running time (seconds)**	7.00	6.96	7.01	9.87	6.93	7935.28	93.63	7286.98	84.39	7.88	66.13

According to the results of the empirical evaluation in figs. S1 and S2, the masking game can achieve 7% higher usefulness and 22% higher fairness with respect to usefulness than the random masking approach does while keeping the privacy and the fairness with respect to privacy at a similar level. Neither can the other baseline scenario based on the *k*-anonymity protection model (*37*) outperform the masking game in terms of these two types of measures in the experiments (see notes S7 and S8 for details).

### Sensitivity and robustness analyses on parameters and settings based on simulated datasets

**Fig. 4.**
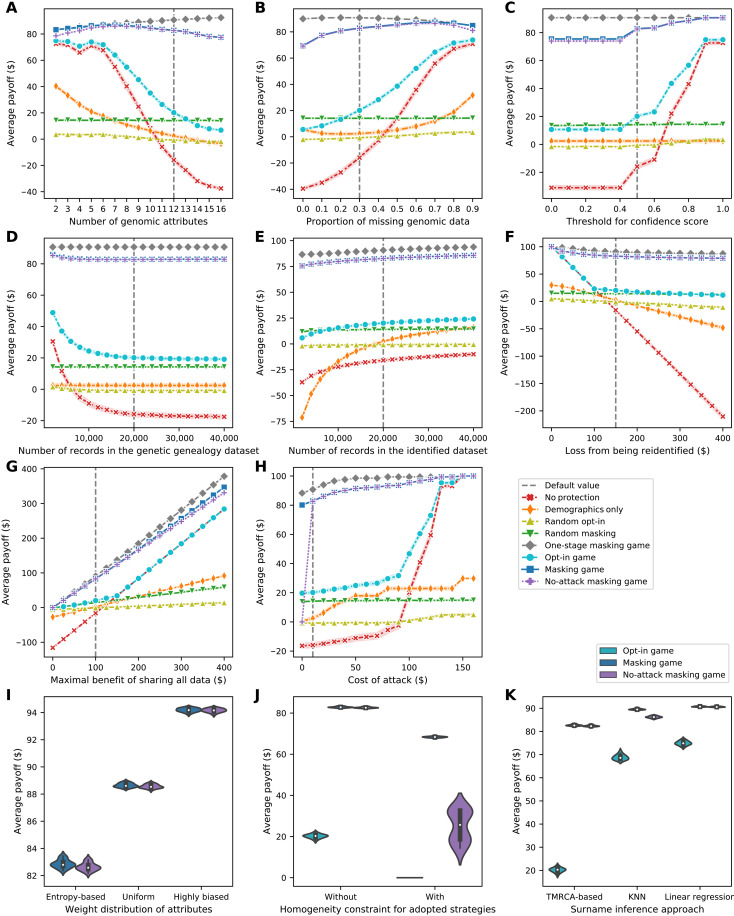
Sensitivity of the data subjects’ average payoff as a function of the parameters and settings in the model. (**A**) Line plot of the sensitivity to the number of genomic attributes. (**B**) Line plot of the sensitivity to the proportion of missing genomic data. (**C**) Line plot of the sensitivity to the threshold for confidence score. (**D**) Line plot of the sensitivity to the number of records in the genetic genealogy dataset. (**E**) Line plot of the sensitivity to the number of records in the identified dataset. (**F**) Line plot of the sensitivity to the loss from being re-identified. (**G**) Line plot of the sensitivity to the maximal benefit of sharing all data. (**H**) Line plot of the sensitivity to the cost of an attack. (**I**) Violin plot of payoff distribution’s sensitivity to the strategy adoption setting. (**J**) Violin plot of the sensitivity to the surname inference approach. (**K**) Violin plot of the sensitivity to the weight distribution of attributes. Each line plot (depicted using Seaborn) shows data subjects’ average payoffs, with error bars representing SDs, in eight scenarios. Each violin plot (depicted using Seaborn) combines boxplot and kernel density estimate for showing the distributions of data subjects’ average payoffs in several scenarios. Gaussian kernels are used with default parameter settings. TMRCA, time to most recent common ancestor; KNN, *k*-nearest neighbors.

To test the model’s sensitivity to eight parameters and three experimental settings, we compared effectiveness measures in eight scenarios across 11 sets of experiments. In each set of experiments, we changed one parameter or experimental setting and ran 20 times with different sample datasets. The results of the sensitivity analysis on eight parameters and three settings regarding the data subjects’ average payoff are shown in Fig. 4.

In general, regardless of how the targeted parameter or setting varies, the subjects’ average payoff in the masking game is much higher than their average payoff in other scenarios (except two variations of the masking game). An even higher average payoff is achieved in the one-stage masking game. In addition, almost the same payoff is achieved with a guarantee of full privacy protection in the no-attack masking game.

In addition, in the masking game, the subjects’ average payoff is not sensitive to most parameters except the maximal benefit of sharing all data. It is attributable to the robustness of the masking game that, for most parameters, there exists at least one scenario in which the range of payoff change is more than six times of the one in the masking game. However, the sensitivity of the payoff to the maximal benefit of sharing all data is unavoidable because the payoff and the benefit are linearly and positively correlated regardless of the scenario.

Moreover, in most scenarios, the subjects’ average payoff decreases when any one of three parameters (i.e., the number of genomic attributes, the number of records in the genetic genealogy dataset, and the loss from being re-identified) increases. By contrast, the subjects’ average payoff increases as any one of five parameters (i.e., the proportion of missing genomic data, the threshold for confidence score, the number of records in the identified dataset, the maximal benefit of sharing all data, and the cost of an attack) increases in most scenarios. These trends are reasonable because, for instance, as the proportion of missing genomic data increases, less information from the genetic genealogy dataset can be used, and thus, the attack becomes less successful. By contrast, as the number of records in the genetic genealogy dataset increases, the adversary is more likely to find someone sharing similar genomes with a targeted subject, and thus, the attack becomes more successful.

By changing each one of three important experimental settings (or assumptions) from what we set in default, we found that, in game scenarios, the subjects would earn higher average payoff if (i) the attributes’ weights are set further away from their information entropies, (ii) the subjects in a dataset are not required to adopt the same strategy, or (iii) an off-the-shelf machine learning approach is used for surname inference in the attack. These trends indicate that a game model’s effectiveness has a potential to be improved further if some experimental setting is changed from what we set in default. The sensitivity analysis on parameters and settings regarding payoff is described in further detail in note S9.

**Fig. 5.**
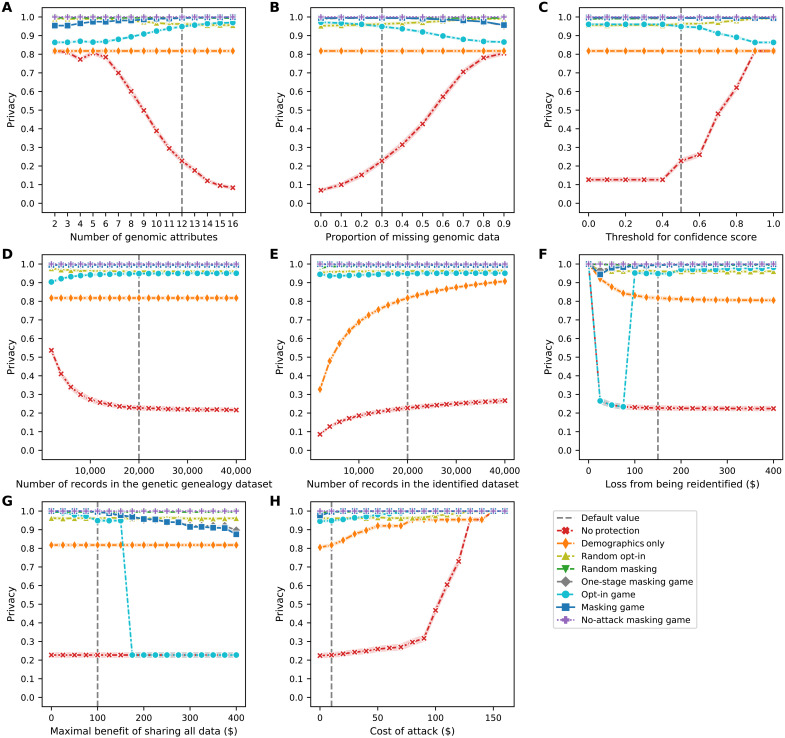
Sensitivity of the data subjects’ average privacy as a function of the parameters in the model. (**A**) Number of genomic attributes. (**B**) Proportion of missing genomic data. (**C**) Threshold for confidence score. (**D**) Number of records in the genetic genealogy dataset. (**E**) Number of records in the identified dataset. (**F**) Loss from being re-identified. (**G**) Maximal benefit of sharing all data. (**H**) Cost of an attack. Each line plot (depicted using Seaborn) shows the data subjects’ average payoffs, with error bars representing SDs, in eight scenarios.

**Fig. 6.**
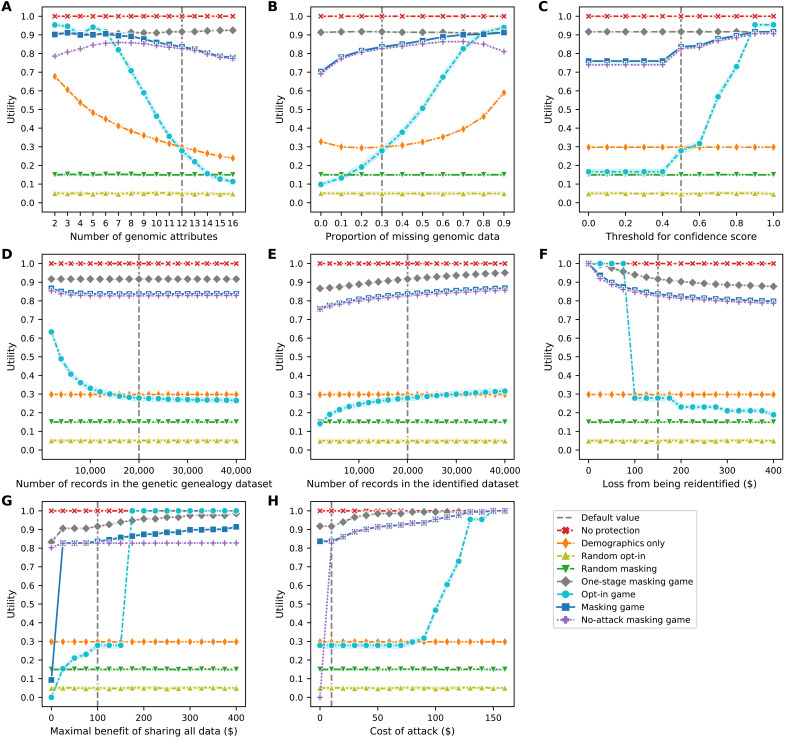
Sensitivity of the data subjects’ average data utility as a function of the parameters in the model. (**A**) Number of genomic attributes. (**B**) Proportion of missing genomic data. (**C**) Threshold for confidence score. (**D**) Number of records in the genetic genealogy dataset. (**E**) Number of records in the identified dataset. (**F**) Loss from being re-identified. (**G**) Maximal benefit of sharing all data. (**H**) Cost of an attack. Each line plot (depicted using Seaborn) shows the data subjects’ average payoffs, with error bars representing SDs, in eight scenarios.

The results of the sensitivity analysis on eight parameters regarding the data subjects’ average privacy and average data utility are shown in Figs. 5 and 6, respectively. In general, the subjects’ average privacy in the masking game is always much higher than their average privacy in baseline scenarios save the random opt-in and random masking scenarios. Likewise, the subjects’ average data utility in the masking game is only lower than their average data utility in the no-protection scenario or in the one-stage masking game. The sensitivity analysis on parameters regarding privacy and utility is described in further detail in note S9.

To investigate how the uncertainty in the data subject’s knowledge about the adversary’s capabilities affects the data subject’s expected payoff, we conducted a robustness analysis on uncertainty in three parameters: (i) the cost of an attack, (ii) the number of records in the genetic genealogy dataset, and (iii) the number of records in the identified dataset. The results are presented in fig. S3. In general, regardless of how the actual value of an examined parameter changes, in most cases, the error in the subjects’ average expected payoff in the masking game is bounded in a range that is much smaller than the difference between the subjects’ average payoff in the masking game and their average payoff in the opt-in game. In addition, the results indicate that the superiority of the masking game in terms of the data subjects’ average payoff is highly robust regardless of how a data subject is uncertain about each of these examined parameters and the corresponding probability of an attack’s success. The robustness analysis on uncertainty in parameters is described in further detail in note S10.

Last, we performed a sensitivity analysis on a special parameter, minority-support factor, which is an exponent in a benefit function considering both usefulness and fairness with respect to usefulness, indicating the extent to which the benefit function supports the minority groups in the shared dataset. In general, the minority groups receive exponentially higher benefits than the majority groups when the parameter is positive and vice versa when the parameter is negative (see note S2 for details). The results, as presented in fig. S4, show that the masking game outperforms other protection approaches in terms of usefulness when the parameter is in the [0, 4] range and that the masking game outperforms other protection approaches in terms of fairness with respect to usefulness when the parameter is in the [0.5, 1.5] range (see note S11 for details).

### Experiments based on Craig Venter’s data and the Ysearch dataset

In this set of experiments, we used a case study to illustrate how our model could be applied to real-world datasets. Specifically, we used Craig Venter’s demographic attributes (including year of birth, state of residence, and gender) and 50 Y-STRs profiled from his genome sequence as the targeted dataset *D*. In addition, we used the Ysearch dataset as the genetic genealogy dataset *D_G_*. After filtering out records with too few targeted Y-STRs, we got a dataset of 58,218 records and 50 Y-STRs with a missing proportion of approximately 26%. Last, we used Intelius.com and the 2010 U.S. Census as sources of the identified dataset *D_I_*.

A query of Craig Venter’s demographic attributes and surname on Intelius.com returned with two records, one of which is Craig Venter. According to the 2010 U.S. Census, 157,681 people were estimated to share the same values on the three demographic attributes with Craig Venter in the United States in 2018. This number indicates that, without the correctly inferred surname, the re-identification would be unlikely to be successful. For simplicity, we masked only genomic attributes instead of all attributes in this demonstrational case study. The parameters in both sets of experiments were set in the same way. For example, the data utility corresponding to each genomic attribute in this set of experiments was set according to its information entropy in the Ysearch dataset. Settings for all other parameters are reported in table S1. We ran the experiments using the greedy algorithm with pruning, the results of which are provided in Table 4. In addition to showing the effectiveness measures of the recommended strategies from eight scenarios, it shows effectiveness measures of all searched strategies in the masking game (with the best strategy being marked out).

**Table 4.** Results for the case study based on Craig Venter’s data and the Ysearch dataset. Scenarios include (1) no protection, (2) demographics only, (3) random opt-in, (4) random masking, (5) opt-in game, (6) masking game, (7) no-attack masking game, and (8) one-stage masking game. Only genomic attributes could be masked in this case study.

**Searched suboptimal strategy**	**Number of shared Y-STRs**	**Inferred surname**	**Confidence score**	**Utility**	**Privacy**	**Benefit**	**Loss**	**Payoff**	**Scenarios recommend**
0	50	Venter	0.6688	1	0.5	$100	$75	$25	1
1	49	Venter	0.6688	0.9967	0.5	$99.67	$75	$24.67	/
2	48	Venter	0.6688	0.9933	0.5	$99.33	$75	$24.33	/
3	47	Venter	0.6688	0.9897	0.5	$98.97	$75	$23.97	/
4	46	Venter	0.6688	0.9862	0.5	$98.62	$75	$23.62	/
5	45	Venter	0.6688	0.9825	0.5	$98.25	$75	$23.25	/
6	44	Venter	0.6688	0.9786	0.5	$97.86	$75	$22.86	/
7	43	Venter	0.6435	0.9746	0.5	$97.46	$75	$22.46	/
8	42	Venter	0.5484	0.9706	0.5	$97.06	$75	$22.06	/
9	41	Venter	0.5481	0.9661	0.5	$96.61	$75	$21.61	/
10*	40	Karlsson	0.4662	0.9593	1	$95.93	$0	$95.93	6, 7
11	39	Karlsson	0.4661	0.9544	1	$95.44	$0	$95.44	/
/	0	/	/	0.44	0.00	$44	$0.00	$44.00	2
/	0	/	/	0	1	$0	$0	$0	3, 5
/	7	Hinze	0.4046	0.5043	1	$50.43	$0	$50.43	4
/	50	/	/	1	0.00	$100.00	$0.00	$100.00	8

*The best strategy in the masking game, found using the greedy algorithm with pruning.

From Table 4, it can be seen that the best strategy in the masking game is superior to all other strategies searched in the game, as well as all strategies recommended from all baseline scenarios and the opt-in game in terms of the resulting payoff. The underlying reason is that the first strategy during the search process with a confidence score below the threshold of 0.5 has an incorrectly inferred surname, which leads to an unsuccessful re-identification and thus successful protection.

All strategies searched in the masking game using the greedy algorithm with pruning are plotted in fig. S5, showing the relationships between three evaluation metrics (i.e., utility, payoff, and inference correctness) and the confidence score for each strategy. The optimality of a strategy depends on the correctness of the surname inference (i.e., the success of stage I of the attack), which is correlated with the threshold for confidence score in the attack model (see note S12 for details).

## DISCUSSION

The methodology described in this study enables subjects to make informed data sharing decisions in the face of complex state-of-the-art re-identification models. It enables people to answer questions such as, “Should I share my de-identified data record to an open data repository?” and “Which portion of my data record should I share to an open data repository?” Moreover, the methodology is sufficiently flexible to enable subjects to make decisions in platforms where sharing partial or modified data is allowed.

Our illustration of this methodology in the context of a known multistage attack on genomic data led to several notable findings. First, although an additional stage can substantially increase the accuracy of the re-identification when there is no protection, it makes the attack more vulnerable to our game theoretic protection because the adversary could be tricked into inferring wrong intermediate information, thus mitigating the privacy risk. Second, most subjects (acting rationally) would choose not to share data to an open data repository (e.g., the Personal Genome Project) if partial data sharing is not permitted. By contrast, most people would share most of their data if sharing partial data is permitted. This finding is intriguing because it suggests that providing subjects with options could encourage a greater degree of data sharing while avoiding re-identification. Third, subjects can choose strategies that allow for sharing a substantial amount of data with a payoff almost as high as the optimal solution, while ensuring that it is never beneficial for the data recipient to attempt re-identification, thus predicting no attack and zero risk within the context of our modeling framework. Last, our extensive sensitivity analysis illustrates how parameters of our model influence a subject’s behavior differently, which can provide guidance to other stakeholders. For example, to effectively promote data sharing in general, policy-makers could increase penalties for detected privacy breaches, and data holders could increase rewards for data sharing. In addition, the analysis shows what weight a data subject should give to each parameter. Specifically, considering the sensitivity in the masking game, a subject should take extra care when the maximal benefit of sharing all data or the cost of an attack is low. The analysis demonstrates the robustness of the methodology that, while an adversary can push most parameters such as damages of attacks and sizes of datasets to a risky point for subjects in poorly protected scenarios, the highly effective and stable protection provided by the masking game is almost immune to these risks.

Limitations exist in our current model, which provide directions for our future work. First, we only considered one adversary and performed experiments with two-stage attacks. In the future, we will consider game theoretic models with multiple adversaries and conduct experiments with attacks that have more than two stages. For instance, in each stage, the adversary could infer a set of attributes using an external dataset or launch a linkage attack, as illustrated by attacks with far more than two stages (*20*, *47*). This is a challenging task because the chain of attack could expand over time. For example, a previously safe and trusted database may start to be targeted and attacked by adversaries if it loosens its access policies or if its vulnerabilities are found. Second, we used a simplified decision-making model in which either each subject makes the sharing decision independently, or all subjects pick the same protection strategy. In the future, we will consider interactions among related data subjects [e.g., family members (*42*)]. Third, the players modeled in our current game model do not fully reason about all the uncertainties. Both players might have incomplete and/or imperfect information. To be more realistic, more complex game theoretic models, such as the Bayesian game, can be used to model incomplete and/or imperfect information. Although our approach appears robust to uncertainty in critical parameters, further qualitative investigation [e.g., a behavioral empirical study (*48*)] would help ensure that these parameters are representative of human decision-making and that they are estimated accurately. Fourth, while we have reasoned from the perspective of a data holder (e.g., the controller of a database) to analyze and control two types of downstream effects of data sharing (i.e., usefulness and fairness), we have not investigated the data holder’s optimal strategy. One potential strategy would be to control the monetary amount paid to subjects for sharing data. However, paying subjects for the loss of privacy is a questionable practice that requires further investigations into the ethical foundations of data sharing and the corresponding ethical and societal implications. When this strategy may be permissible, future investigations could model the data holder as a player in the game, considering the interaction between a subject and a data holder and the interaction between a data holder and an adversary, to uncover the data holder’s optimal strategy. Fifth, similar to most suppression-based disclosure control methods [e.g., *k*-anonymity (*49*), *l*-diversity (*50*), and *t*-closeness (*51*)], our game theoretic model might induce values that are missing not at random (MNAR) in the released dataset. In recognition of the fact that MNAR may not be desirable in certain applications, it should be noted that data could be suppressed or masked in a completely random manner. However, as our experiments illustrate, this strategy leads to sharing substantially less data.

Our game theoretic model could be applied to other multistage privacy attacks such as membership inference and genome reconstruction attacks (*52*–*55*). Since several studies have shown correlations between SNP and STR markers (*56*, *57*) and the Gymrek attack infers surnames from datasets with only Y-STR markers, it is worth examining the effectiveness of our protection model against multistage attacks involving datasets with SNP markers. For those attacks that might require greater computational effort and efficiency, we believe that the performance of the search algorithms could be improved through the assistance of distributed computing architectures or graphical processing units as they can process multiple computations simultaneously. To handle the high computational cost of solving complex game models, especially in the face of multistage attacks, a type of game-as-a-service architecture has the potential to be deployed in cloud servers to provide distributed game modeling and solving as a service to data subjects. We believe that little training effort would be required for an individual to use our game solver. Alternatively, it is possible that our solution could be integrated as a service into existing data anonymization software, such as ARX (*58*), which has already been expanded to include a general game theoretic module for risk analysis (*59*). Even if some parameter required for the game theoretic module is not set accurately, the resulting strategy might still be effective, as our sensitivity and robustness analyses have demonstrated.

## Supplementary Materials

### This PDF file includes:

Notes S1 to S12 Figs. S1 to S10 Tables S1 to S11 Legend for data S1 References

## Notes

View/request a protocol for this paper from *Bio-protocol*.
