# Supplementary material for: Using game theory to thwart multistage privacy intrusions when sharing data
Source: Sci Adv. 2021 Dec 10;7(50):eabe9986. doi: 10.1126/sciadv.abe9986 (PMC8664254; doi:10.1126/sciadv.abe9986)
Supplement: Supplementary file 1 — Notes S1 to S12 Figs. S1 to S10 Tables S1 to S11 Legend for data S1 References [file sciadv.abe9986_sm.pdf]

Science Advances
AAAS

Supplementary Materials for

Using game theory to thwart multistage privacy intrusions when sharing data

Zhiyu Wan*, Yevgeniy Vorobeychik, Weiyi Xia, Yongtai Liu, Myrna Wooders, Jia Guo, Zhijun Yin, Ellen Wright Clayton, Murat Kantarcioglu, Bradley A. Malin

*Corresponding author. Email: zhiyu.wan@vanderbilt.edu

Published 10 December 2021, *Sci. Adv.* **7**, eabe9986 (2021)
DOI: 10.1126/sciadv.abe9986

The PDF file includes:

Notes S1 to S12
Figs. S1 to S10
Tables S1 to S11
Legend for data S1
References

Other Supplementary Material for this manuscript includes the following:

Data S1

Supplementary Note S1. A review of related literature.

Here, we reviewed related works, regarding genomic data sharing, from various aspects: (i) re-identification attacks, (ii) data masking protections, (iii) participant-centric access control, and (iv) risk analyses and economic models.

S1.1. Re-identification attacks targeting genomic data sharing

In a typical re-identification attack, the adversary re-identifies an anonymous record by linking it to an identified dataset upon a set of common attributes called quasi-identifiers (*60*, *61*). Malin and Sweeney first demonstrated re-identification attacks on genomic datasets (*15*, *16*). By contrast, in our adversarial model, the adversary re-identifies genomic records in a multistage manner.

Sweeney, Abu, and Winn re-identified participants of the Personal Genome Project (*44*) by linking these participants' data records to voter registration lists upon three demographic attributes (namely, gender, birth date, and ZIP code), and verified results by leaked names embedded in the filenames (*17*). Their attack demonstrates the privacy risk of an open-access database. We considered a similar open-access database that is targeted by the adversary in our adversarial model. However, our re-identification attack model's record linkage stage is based on only two demographic attributes (namely, year of birth and state of residence). They inferred the full name of a record from the filename and used it for verification. By contrast, we inferred the surname from the genomic attributes and used it as a quasi-identifier in the record linkage stage.

Gymrek, McGuire, Golan, Halperin, and Erlich re-identified nearly 50 participants of the 1000 Genomes Project by first inferring surnames from Y-STRs, and then linking genomic records to identified public resources upon demographics and those inferred surnames (*18*). We used the same attack in our adversarial model. However, some datasets they used for surname inference (e.g., the Sorenson Molecular Genealogy Foundation) are no longer available. In addition, their datasets all lack the ground truth. As a result, we used large-scale simulated datasets in addition to their datasets in our experiments. Additionally, we developed a risk assessment framework based on which we can evaluate different protection approaches.

Erlich, Shor, Pe'er, and Carmi estimated that, in a consumer genomic database (e.g., GEDmatch) of more than one million individuals, 60% of individuals of European descent would be linked to a family member as far as a third cousin (*62*), similar to the case of the Golden State Killer. Their estimation was based on theoretical models and simulations. Subsequently, Kim, Edge, Algee-Hewitt, Li, and Rosenberg demonstrated the possibility of performing these familial searches of short tandem repeat (STR) databases using single-nucleotide polymorphism (SNP) profiles or vice versa (*57*). We considered the same type of STR databases. However, the adversary in our model only links targeted individuals to themselves instead of their family members.

Ney, Ceze, and Kohno demonstrated that an entire consumer genomic database could be extracted by uploading artificial records (*63*). Their attack not only can re-identify anonymized

genomic records but also can convert a query-based database into an open-access database, demonstrating the vulnerability of a recreational genomic database. In the scenario where users left GEDmatch due to privacy concerns, Ney *et al.*'s attack showed that a potential adversary might have extracted the entire database beforehand even if the data of users who have left are deleted (*63*). Following their attack, our model can protect open-access research databases [e.g., OpenSNP (*11*)] and query-based consumer genomic databases (e.g., GEDmatch).

Lippert *et al.* re-identified records in a whole-genome sequencing dataset by first inferring visual traits from genotypes (*21*). Other works re-identified records by first inferring genotypes from phenotypes [e.g., quantitative traits (*19*), visual traits (*64*), 3D facial traits (*22*)] or summary statistics like linkage disequilibrium (*52*, *65*, *66*). Our protection model has the potential to deal with these multistage re-identification attacks, although the inferred attribute in our experiments is the surname instead of a phenotypic attribute.

S1.2. Masking approaches protecting genomic data sharing

Masking approaches belong to a family of approaches (namely, privacy-preserving data sharing approaches) that can protect anonymity and mitigate privacy risks by modifying data before sharing. Privacy-preserving data sharing approaches have been introduced to protect genomic data before sharing [e.g., generalization (*39*, *67*), masking (*7*, *68–71*), noise addition (*40*, *72*, *73*), and synthetic data generation (*74*, *75*)]. Note that, the noise addition approaches (*40*, *72*) based on differential privacy (*38*), that have been applied to sharing summary statistics about genome-wide association studies are not directly applicable in our scenario of sharing individual-level genomic data. However, privacy-preserving synthetic data generation approaches based on deep learning models [e.g., generative adversarial networks (*74-76*) and restricted Boltzmann machines (*75*)], either differentially private (*74*, *76*) or not (*75*), have been applied to sharing individual-level biomedical data and could be used to generate individual-level genomic data for sharing as well.

By applying the masking approaches to individual-level genomic data sharing, Humbert, Ayday, Hubaux, and Telenti maximized the utility while protecting genomic privacy in a family by optimizing the way of masking SNPs (*69*). Afterward, Kale, Ayday, and Tastan revisited the problem with an improved approach (*70*). Our protection model also quantified an individual's utility and privacy and optimized the way to mask genomic attributes. However, we considered STR instead of SNP markers and chose a different way to solve the multi-objective optimization problem. Besides, we did not consider other family members' privacy risks. Because of well-established genotype imputation techniques and the correlations between genomic attributes, masking approaches are more suitable for independent genomic attributes (*77*, *78*). The STR markers considered in our experiments do not have strong correlations, making the masking approach suitable for our problem.

S1.3. Participant-centric access control for genomic data sharing

Participant-centric access control allows the original participant to control access to their genomic data (*7*, *14*). Kaye *et al.* overviewed and analyzed various participant-centric biomedical research initiatives that protect the privacy and maintain public trust (*79*). Clayton,

Halverson, Sathe, and Malin systematically reviewed works that consider genomic data privacy from individuals' perspectives (*80*). We designed data sharing strategies from the participants' perspectives and conducted experiments accordingly.

Through a single-blinded randomized trial in a healthcare environment, McGuire *et al.* found out that 53.1% of participants are willing to publicly release their genomic data with no controls (*81*). We would argue that participants in the healthcare environment care more about the utility of health data. In addition, the number of high-profile privacy attacks targeting genomic data keeps increasing recently (*7*, *35*).

Through a randomized trial in a healthcare environment, Oliver *et al.* found out that privacy risks were largely outweighed by utility regarding genomic data sharing and urged policy makers to respect participants' diverse assessments on the privacy risks and on the utility and participants' privacy-utility determinations (*82*). We would argue that participants' intuitive assessments of their privacy risks are not accurate, so we used an analytical model and concrete experiments with various cost-and-benefit combinations to help participants and policy makers make more informed and strategic decisions.

Deuber *et al.* developed a system based on garbled circuits that enables a data subject to arbitrarily decide which third-party can compute over their encrypted genomic data stored in the cloud (*83*). Whereas in our model, the data subject decides to share data with an open-access database, so no encryption is required. Furthermore, our model aims to provide an optimal sharing strategy.

Roberts *et al.* concluded that paying individuals for genomic data sharing benefits both the company and consumers and they asked for the best ways to incentivize individuals to share data (*12*). For example, Nebula Genomics facilitates a blockchain-based genomic data marketplace where data subjects are incentivized to share their private data by selling data directly to third parties (e.g., researchers) using its cryptocurrency, while protecting the privacy of data subjects (*84*). Other companies, like EncrypGen and Luna DNA, have similar mechanisms (*85*). More recently, Blockchain has been utilized to enable privacy-preserving genomic-data analysis in a personally controlled (*14*) and transparent manner (*86*). We considered a similar economic model in our system where data subjects are incentivized to share data, and our model can reveal an optimal sharing strategy to each data subject.

S1.4. Risk analyses and economic models for privacy-preserving genomic data sharing

Accurately assessing utility and privacy risk is the first step before optimizing the utility while protecting privacy (*87*). Usually, privacy risk is quantified with a specific attack in consideration. Humbert, Ayday, Hubaux, and Telenti proposed a reconstruction attack in which the adversary can infer the target's genome sequence from his or her relatives' sequences (*88*). Additionally, they quantified the level of genomic privacy as a result of the proposed attack. More powerful reconstruction attacks are proposed to infer individuals' genotypes from their relatives' genotypes and phenotypes with the interdependent privacy risks being quantified (*54*, *89*). Craig *et al.* discussed approaches for assessing and quantifying the privacy risks to participants that result from the sharing of summary-level data with a membership inference

attack in consideration (*90*). Wan et al. quantified both utility and privacy and designed a measurement (*91*) that accelerates the search process for the best protection strategy against the Shringarpure and Bustamante's membership inference attack (*92*). Wagner systematically compared and evaluated 24 genomic privacy metrics in four possible attack scenarios, in all of which the adversary aims to infer a person's genomic markers (*93*). Compared to these quantification models, our model considers a multistage re-identification attack instead of a one-stage attack (e.g., reconstruction attack, genotype inference attack, membership inference attack). Moreover, our model not only quantifies both utility and privacy but also optimize them simultaneously. In addition, the adversarial behaviors in most of these adversarial models are pre-fixed, instead of strategically determined, which might be an assumption too simplified for a real-world scenario.

Game theory, as a branch of applied mathematics, studies the strategic interactions among rational decision makers (*45*). With its initial focus on economics (*94*), it has influenced many other fields including social science, biology, and computer science (*95*). Two-player game models have been recently applied in machine learning and statistics to optimally solve data-driven problems (*96*, *97*). Game theoretic approaches have been applied to various problems regarding data privacy (*98–101*). Wan *et al.* reviewed all game theoretic models applied to privacy-preserving genomic data sharing problems (*102*).

First, Wan *et al.* developed a re-identification game model that protects individual-level demographic data and health data against record linkage attacks based on a two-player Stackelberg game model (*41*). Moreover, the re-identification game has been implemented and integrated into an open-source toolkit: ARX (*59*). We used the same game theoretic model, which is ready to be integrated into an open-source programming package. However, the re-identification attack we considered here has multiple stages, strengthening the previous attack, potentially bringing more challenges to the protection model, and making the game solving process computationally more complex. Additionally, we masked individual-level demographic and genomic attributes instead of generalizing summary statistics of demographic attributes in the dataset.

Meanwhile, Humbert, Ayday, Hubaux, and Telenti used a game theoretic framework (*42*) to study how one individual's decision regarding sharing genomic data would impact his or her family members' privacy risks considering the reconstruction attack they proposed (*88*). Their game model only regards data sharers (or subjects) as players. By contrast, our game model regards both the adversary and each data subject as players.

More recently, Wan *et al.* developed a membership inference game that protects summary-level genomic data against a membership inference attack based on a two-player Stackelberg game model (*43*). We used the same game theoretic model. However, our attack has one more stage after the attribute inference stage (surname as the attribute in our attack). Additionally, we helped data subjects share individual-level STR data instead of helping data holders share summary-level SNP data.

Supplementary Note S2. Design of the benefit function considering usefulness and fairness with respect to usefulness of the shared dataset.

The data holder has the ability to control the benefit function of a data sharing strategy $b(\mathbf{s})$ for any data subject, and the data holder might be allowed to do so under certain circumstances. When a data holder cares about the usefulness (see Eq. S18 in note S7 for its definition) and the fairness with respect to usefulness (see Eq. S19 in note S8 for its definition) of the collected data, they can change a rational data subject's optimal sharing strategy by implementing a well-designed benefit function when they are allowed to do so.

The benefit of sharing should be designed to increase monotonically as the amount of shared data increases until it reaches the limit. The benefit of sharing for a data subject is a function of the data subject's sharing strategy **s**, given both the shared and the unprotected datasets, and it can be designed as a truncated linear function as shown in Eq. S1:

$$b(\mathbf{s}) = BU = B \times \min\left(\frac{1}{\sum_{j=1}^{m} w_j} \sum_{j=1}^{m} w_j \beta_j^{\alpha} s_j \,, 1\right), \tag{S1}$$

where B is the maximal benefit of sharing all data for each subject, U is the subject's data utility, m is the number of attributes, w_j is the jth attribute's weight set as the information entropy in either the genetic genealogy dataset D_G or the identified dataset D_I, s_j is the data subject's sharing decision for the jth attribute, and $\alpha \in (-\infty, \infty)$ is the minority-support factor. In addition, $\beta_j \in (0, \infty)$ is the minority level which indicates the proportion of the data subject's group (as shown in Fig. S6) in terms of the jth attribute in the population. It is defined as shown in Eq. S2:

$$\beta_j = \log_2\left(\frac{1}{n_{g,j} r_{g,j}} + 1\right), \forall j \tag{S2}$$

in which $n_{g,j}$ is the number of groups in the jth attribute, and $r_{g,j}$ is the proportion (or relative frequency) of the data subject's group in the underlying population in terms of the jth attribute. When the minority level β_j is larger than 1, the data subject tends to be in a minority group in terms of the jth attribute, and when the minority level β_j is smaller than 1, the data subject tends to be in a majority group in terms of the jth attribute.

To illustrate the calculation process of the minority level, we consider an example in which there are three groups for the second attribute, state of residence, in a dataset (namely, California, Tennessee, and Maine) and the size of each group is 50, 30, and 10, respectively. Thus, if the data subject is from California, his or her corresponding minority level is: $\beta_2 = \log_2(90/3/50 + 1) \approx 0.68$. If the data subject is from Tennessee, his or her corresponding minority level is: $\beta_2 = \log_2(90/3/30 + 1) = 1$. If the data subject is from Maine, his or her corresponding minority level is: $\beta_2 = \log_2(90/3/10 + 1) = 2$.

When the minority-support factor α is positive, the benefit function is in favor of the minority group, and when the minority-support factor is negative, the benefit function is in favor of the majority group for all attributes and all data subjects. When the minority-support factor is zero, Eq. S1 becomes a linear function as shown in Eq. S3:

$$b(\mathbf{s}) = BU = \frac{B}{\sum_{j=1}^{m} w_j} \sum_{j=1}^{m} w_j s_j, \tag{S3}$$

in which the entropy-based utility metric U is the same as the weighted precision metric (*103*) with a generalization height of 2 with the entropy-based weight function (*41*, *104*).

To facilitate this benefit function in the game model, we need to emphasize the assumption that the data subject will always share the actual value if they share an attribute in a record because, otherwise, they might gain extra benefit by lying about the shared value.

Supplementary Note S3. Derivation of game models and parameter settings.

S3.1. System model

As shown in Fig. 1 (in the main text), we investigate the problem of privacy-preserving data sharing from the perspective of a system that includes a data subject, three databases, and an adversary. Our model's goal is to help a data subject, given a multistage attack model, decide whether and how to share person-specific data with the targeted database (i.e., the database in the middle). In our model, the data subject wants to share data for various reasons but has privacy concerns. In addition, the targeted database only releases de-identified (i.e., without personal identifiers) datasets to the public or a third party. The dataset recipient could be a malicious adversary who wants to re-identify the targeted data subject's record from the dataset and has limited incentives, abilities, and resources.

In this system, data flow from the data subject to the adversary through three channels. Databases in the first channel are public identified databases such as a voter registration list (*61*), a public record search engine [e.g., Intelius (www.intelius.com) and PeopleFinders (www.peoplefinders.com)] or a social media service (*105*) [e.g., Facebook (www.facebook.com) and LinkedIn (www.linkedin.com)]. These data are usually associated with real identities. Let us assume that some personal identifiers and demographic attributes of the data subject are accessible to the adversary through one of these public databases.

Databases in the second channel are not likely to be targeted by the adversary because of their well-established data sharing policies. For example, they only share anonymous granular data with a limited set of trusted third parties, and they only share summarized or anonymized individual-level data (i.e., with no demographic information or with rigorous risk mitigation) to the public. Databases in this channel are usually controlled by large non-profit organizations [e.g., the Global Alliance for Genomics and Health (ga4gh.org)], government-sponsored research programs [e.g., All of Us Research Program (*1*) (allofus.nih.gov) and UK Biobank (*106*) (www.ukbiobank.ac.uk)] and large direct-to-consumer genetic testing (DTC-GT) companies [e.g., 23andMe (23andme.com) and AncestryDNA (www.ancestry.com/dna)]. Although data subjects associated with data in this channel are not necessarily targeted by the adversary, the adversary can use their data to infer additional attributes of targeted data subjects, as illustrated in our attack model.

The adversary targets databases in the third channel because either their data sharing policies are vulnerable to privacy attacks, or their datasets are openly accessible, making them less trustworthy. Databases in this channel include recreational genomic databases [e.g., GEDmatch (www.gedmatch.com)] and open-access databases [e.g., OpenSNP (*11*) (opensnp.org) and the Personal Genome Project (*44*) (www.personalgenomes.org)]. Compared with the second channel, the third channel is more likely to allow a data subject to choose which part of a record can be shared. By contrast to the first channel, the third channel allows a data subject to share de-identified genomic and phenotypic data. Genomic data shared in this channel are vulnerable to various attacks such as re-identification (*17*, *18*) and data extraction attacks (*63*). Because the re-identification attack is more typical than other attacks, we specifically

investigate the problem of optimal data sharing in this channel against a multistage re-identification attack.

S3.2. Attack model

The attack model considered in this system has multiple stages. Let us use a two-stage attack as an example. Before an attack, the adversary receives a piece of information (x) about a target (i.e., a targeted data subject) from a dataset (D). In stage I, the adversary infers a piece of information (x_G) about the target according to another dataset (D_G) and information x. In stage II, a piece of information (x_I) about the target is learned by querying another dataset (D_I) with x and x_G. Alternatively, the adversary may query dataset D_I with x only (without inferring x_G), which reduces the number of attack stages to one. Still, if additional information gets inferred in stage I, the prediction in the two-stage attack tends to be more accurate than the one in the one-stage attack. In general, the attack can have more than two stages, in which each stage infers a piece of new information, based on an additional dataset, which can be used in subsequent stages. In general, the prediction in a multistage attack tends to be more accurate than predictions in the attack's variations with fewer stages.

Fig. S7A illustrates the two-stage attack executed based on Gymrek *et al.*'s work (*18*), which we refer to as the Gymrek attack. This attack blends surname inference with record linkage. More formally, in this attack, D is a dataset with genomic and demographic attributes as information x; D_G is a genetic genealogy dataset with genomic attributes as the first part of information x and with surnames as information x_G; and D_I is an identified dataset with demographic attributes as the second part of information x_G, with surnames as information x_G, and with first names as information x_I. More specifically, in their experiments, Gymrek *et al.* used extracted short tandem repeats on the Y chromosome (Y-STRs) from Ybase (www.ybase.org, archived at https://web.archive.org/web/20101223130218/http://ybase.org in 2010), the 1000 Genomes Project, and the National Center for Biotechnology Information archives as genomic dataset D, with demographic attributes (namely, year of birth and state of residence) (*18*). They also used records from Ysearch and Sorenson Molecular Genealogy Foundation as genetic genealogy dataset D_G, with attributes including Y-STRs and surnames. Last, they used the record search engine, PeopleFinders, based on mined records from voter and driver registries, as identified dataset D_I, with attributes including year of birth, state of residence, first name, and surname. We use the same surname inference and record linkage methods as Gymrek *et al.* used and further assume that an adversary randomly attacks one matched identified record in the final re-identification stage.

As in the Gymrek attack model, we assume that the database holding dataset D releases the entire dataset on an individual level and that databases holding datasets D_G and D_I provide query services. Note that the attack can be executed either before or after data are shared through the first two channels. The inferred surname in stage I will generally be correct if a record corresponding to the data subject is in dataset D_G when the attack happens. The attack will likely fail if the data subject's record is not in the dataset D_I when the attack happens. Thus, for simplicity, in our experiments, we assume that only dataset D_I (instead of dataset D_G) includes a record corresponding to the data subject when the attack happens.

S3.3. Protection model

To mitigate the re-identification risk, Gymrek *et al.* recommended protecting the data through data masking (i.e., hiding some data) and access control (*18*). However, these strategies' performances have not been empirically evaluated or strategically analyzed in the context of a multistage attack. Without a proper protection strategy, responses to these attacks in practice could be shrinkage of the user base or closure of the entire database, which could substantially harm the data utility and hamper scientific progress.

In our framework, we determine the optimal protection strategy for a data subject to share personal data, assuming they are rational and driven by incentives. To do this, we consider three typical scenarios. These scenarios are slightly different in complexity, with the first the simplest and the last the most complex. We further assume that all databases' sharing strategies are fixed and known to all parties in the system in each scenario.

A. Always-attack scenario

In the always-attack scenario, we consider the worst case in which the adversary acts as if he or she has unlimited resources and incentives (i.e., always attacks), and the data subject makes a binary decision on whether to share personal data with the targeted database. Given the benefit of sharing data (B) (i.e., sharing information x), the loss from being re-identified (L) by the adversary, and the probability of an attack's success (p), the data subject's payoff equals the benefit minus the expected cost:

$$v_d = B - Lp. \tag{S4}$$

For a data subject, the sharing decision (s) can be represented as 1 (i.e., to share) or 0 (i.e., not to share). For a rational data subject, the optimal sharing decision (s^*) will be 0 if and only if the expected payoff is smaller than 0, as shown in Eq. S5:

$$s^* = \begin{cases} 1, & B \geq Lp, \\ 0, & B < Lp. \end{cases} \tag{S5}$$

The parameters in the model can be set according to specific cases. Parameter B's setting depends on the monetary incentive for sharing data or the value of a complimentary service that requires person-specific data. For example, in the case of sharing data with a free relative finder service, it could be set to the price that 23andMe, a DTC-GT company, asked for its relative finder service, valued at \$99. In other cases, it could also be set according to the price of shared data in an online marketplace [e.g., blockchain-based platforms (*107*)], the "data dividends" paid by companies that use shared data [e.g., the EU's TRUSTS project (*108*)], or compensation for participation in a research program [e.g., the All of Us Research Program (*1*)].

Parameter p's calculation depends on the simulation of an attack, which requires knowledge of the attack model and datasets used in the attack. If the attack model is published in a high-impact journal and datasets used in the attack are public, the attack's simulation is possible. For the Gymrek attack, we assume that an adversary randomly attacks one matched identified record

when there are more than one matched identified records in the final re-identification stage. Thus, p is calculated as the reciprocal of the number of matched identified records if the inferred surname is correct. Note that $p = 0$ if an incorrect surname is inferred. As a result, in a two-stage attack, the probability of an attack's success can be represented and calculated as:

$$p = p_1 p_2 = r/k, \tag{S6}$$

in which p_1 is the probability of stage I's success, and p_2 is the probability of stage II's success given that stage I succeeds. Additionally, we have $p_1 = r$ in which r is the correctness of the inferred surname, and we have $p_2 = 1 / k$ in which k is the number of matched identified records in the linkage upon quasi-identifiers (i.e., common attributes in two linkable datasets) and the inferred surname. In the situation that no surname is inferred, we have:

$$p = p' = 1/k', \tag{S7}$$

in which p' is the probability of the stage II's success, given stage I is omitted. In addition, k' is the number of matched identified records available for linkage solely upon their quasi-identifiers. This situation happens when the confidence score is below a threshold (i.e., no surname is inferred) or the number of matched identified records is zero (i.e., the adversary realizes that the inferred surname is incorrect). Note that parameters like p_2 and p' are defined according to the Marketer re-identification risk model (*109*). Because it is assumed that the record corresponding to each targeted data subject is in the identified dataset when the attack happens, we always have $k' \geq k \geq 1$ and $p' \leq p_2 \leq 1$, if the inferred surname is correct.

Parameter L, the loss that an attack brings to a data subject, could be set according to the outcome of a successful attack or the data subject's valuation on privacy. For example, it could be set to the increment of insurance payment (e.g., $150 per year) if the adversary is a life insurance company, and a pathological gene is identified in the target's genome. While the settings of parameters like L may be uncertain, we did extensive sensitivity analysis on these parameters and depicted the results in Figs. 4 (in the main text), S3, and S4.

B. Opt-in game scenario

In the opt-in game scenario, we assume that both the adversary and the data subject make rational decisions. In other words, we assume that the adversary will attack if and only if the benefit of the attack outweighs the cost of the attack (although it is still arguable whether the adversary will attack if the benefit equals the cost). In this scenario, game theory can be naturally harnessed to determine the data subject's best strategy. A typical two-player game model is illustrated in Fig. S7B. Specifically, a two-player Stackelberg (i.e., leader-follower) model is applied to this scenario. In this model, the data subject is the leader, and the adversary is the follower. The data subject's action is to share or not, represented by a binary variable (s). The adversary's action is to attack or not, represented by a binary variable (a). Assuming the expected gain of the adversary is the same as the expected loss of the data subject (Lp), given the expected benefit of a successful attack to the adversary ($L\hat{p}$) and the cost of an attack (C), the payoff to the adversary is:

$$v_a = Lp - C, \tag{S8}$$

and the adversary's estimated payoff is:

$$\hat{v}_a = L\hat{p} - C, \tag{S9}$$

in which $\hat{p}$ is the adversary's estimated probability of an attack's success. Since the adversary does not know the ground truth of the inferred information, the adversary must compute the probability of an attack's success by estimating the correctness of the inference in stage I. For an adversary, given the shared data, the attack decision (a) can be represented by 1 (i.e., to attack) or 0 (i.e., not to attack). For a rational adversary, given the shared data, the optimal attack decision (a^*) will be one if and only if the expected payoff is larger than 0, as shown in Eq. S10:

$$a^* = \begin{cases} 1, & L\hat{p} > C, \\ 0, & L\hat{p} \leq C. \end{cases} \tag{S10}$$

In the case of the Gymrek attack, $\hat{p}$ can be represented and calculated as:

$$\hat{p} = \hat{p}_1 p_2 = \hat{r}/k, \tag{S11}$$

in which $\hat{p}_1$ is the adversary's estimation of the probability of stage I's success, and p_2 is the probability of stage II's success given that stage I succeeds. Additionally, we have $\hat{p}_1 = \hat{r}$ in which $\hat{r}$ is the estimated correctness of the inferred surname, which could be calculated as the confidence score as defined in the Gymrek attack model. In the situation that no surname is inferred, no estimation is required:

$$\hat{p} = p = p' = 1/k'. \tag{S12}$$

This situation happens and only happens when $1/k' \geq \hat{r}/k$ (i.e., $p' \geq \hat{p}_1 p_2$). In other words, we assume that the adversary will only choose to infer a surname if and only if the inference brings the adversary a higher estimated probability of an attack's success. The adversary's optimal decision on whether to infer surname in stage I (a') can be represented by 1 (i.e., to infer) or 0 (i.e., not to infer) and can be calculated as in Eq. S13:

$$a' = \begin{cases} 1, & \hat{p}_1 p_2 > p', \\ 0, & \hat{p}_1 p_2 \leq p'. \end{cases} \tag{S13}$$

The cost of an attack (C) can combine the cost for accessing data, the computing cost, and the cost of expected liquidated damage penalty. For simplicity, we assume that the penalty cost and the computing cost are both negligible. Thus, the parameter C could be set to \$10, which is the cost for accessing a subject's report from PeopleFinders or Intelius. The report contains the

subject's personal information such as phone numbers, home addresses, family members, and property details. Given C = \$10 and L = \$150, a data subject will be attacked if $k \leq 1/\hat{p} < L/C = 15$ (according to Eqs. S10 and S11) or $k' = 1/\hat{p} < L/C = 15$ (according to Eqs. S10 and S12).

The Stackelberg game model in the opt-in game scenario can be represented in the extensive form (shaded in gray), as shown in Fig. 1B (in the main text). The solution to the Stackelberg game (i.e., both player's optimal decisions) can be represented as an optimization problem, as shown in Eq. S14:

$$(s^*, a^*) = \underset{s, a \in \hat{\phi}(s)}{\operatorname{argmax}}(Bs - Lpsa), \quad \hat{\phi}(s) = \left\{a \middle| \underset{a}{\operatorname{argmax}}\left(L\hat{p}sa - Ca\right)\right\}, \tag{S14}$$

in which $\hat{\phi}(s)$ is the set of the adversary's estimated best responses to the data subject's strategy s. Given certain conditions, the optimal solution can be represented in the explicit form, as shown in Eq. S15:

$$\begin{cases} s^* = 1, a^* = 1, & L\hat{p} \geq C, LP \leq B, \\ s^* = 0, a^* = 0, & L\hat{p} \geq C, LP > B, \\ s^* = 1, a^* = 0, & L\hat{p} < C. \end{cases} \tag{S15}$$

According to Eq. S15, the data subject should share data if there is no attack. This situation happens when the cost of an attack is high enough, the loss from being re-identified is small enough, or the estimated probability of an attack's success is small enough (i.e., the number of matched identified records is large enough). Even if there is an attack, a rational data subject will still choose to share data if the benefit of sharing is large enough. Given B = \$100 and L = \$150, a data subject should share no data if $k \leq 1/p < L/B = 1.5$ (according to Eqs. S6 and S15) and $k' = 1/p < L/B = 1.5$ (according to Eqs. S7 and S15) (i.e., if the record that needs to be shared can be uniquely identified).

Note that, when an adversary does not incur any cost (i.e., C = 0), the opt-in game scenario becomes the always-attack scenario. For this reason, the always-attack scenario, a special case of the opt-in game, is not considered as an independent scenario in our experiments.

C. Masking game scenario

The masking game is almost the same as the opt-in game except that, we consider a data subject with more control over data sharing in the masking game scenario. Instead of a binary decision on whether or not to share the whole data record, the data subject is afforded a larger action space. When uploading data to GEDmatch, OpenSNP, or the Personal Genome Project, the choices a data subject can invoke to modify data include i) uploading only a portion of it or ii) uploading fake data. However, both data volume and data quality affect service quality.

Thus, data subjects are incentivized to share more, as well as authentic, data. Here, we assume that the data subject will upload only authentic data. For simplicity, we further assume that the only action the data subject can take is masking. In other words, the data subject's action determines whether to mask or share each attribute in his or her record. It is represented by an m-dimensional vector of binary elements: $\mathbf{s} = <s_1, \cdots, s_j, \cdots, s_m> \in \mathbb{B}^m$, in which m is the number of attributes in the record, with $s_j = 0$ if j^{th} attribute is masked or $s_j = 1$ if j^{th} attribute is shared (i.e., not masked). Each strategy of the data subject corresponds to one action. As a result, the size of the strategy space for the data subject increases exponentially as a function of the number of attributes. For illustration, let us assume that only fixed strategies are considered. For a record with m attributes, the number of sharing strategies is 2^m, the same as the number of the adversary's decision nodes. Given a sharing strategy (or shared data), the adversary's action (a) is either 1 (i.e., to attack) or 0 (i.e., not to attack). A strategy of the adversary specifies the adversary's action at each of his or her decision nodes.

The benefit of sharing $b(\mathbf{s})$, as a function of the data subject's sharing strategy, increases monotonically as the amount of shared data increases until it reaches the limit (see note S2 for details about a well-designed benefit function). Note that other benefit functions can be used here as well.

The other variables in this scenario that need to be considered include the probability of an attack's success, $p(\mathbf{s})$, and adversary's estimated probability of an attack's success, $\hat{p}(\mathbf{s})$, which can be regarded as functions of the sharing strategy. It is expected that the attack will become less successful as the amount of shared data becomes smaller.

The Stackelberg game model in the masking game scenario can be represented in the extensive form, as shown in Fig. 1B (in the main text). The payoff functions for both players, given a fixed sharing strategy s, are calculated as follows. Given the shared data, if an adversary attacks a data subject, the data subject's payoff is $b(\mathbf{s}) - Lp(\mathbf{s})$, and the adversary's estimated payoff is $L\hat{p}(\mathbf{s}) - C$. Alternatively, if the adversary does not attack, the data subject's payoff is $b(\mathbf{s})$, and the adversary's payoff is zero.

The data subject's best sharing strategy ($\mathbf{s}^*$) and the adversary's corresponding best response (a^*), as the solution to the Stackelberg game, is shown in Eq. S16:

$$\begin{gathered}(\mathbf{s}^*, a^*) = \underset{\mathbf{s}, a \in \hat{\phi}(\mathbf{s})}{\operatorname{argmax}}(b(\mathbf{s}) - Lp(\mathbf{s})a), \\ \hat{\phi}(\mathbf{s}) = \left\{a \middle| \underset{a}{\operatorname{argmax}}\left(L\hat{p}(\mathbf{s})a - Ca\right)\right\},\end{gathered} \tag{S16}$$

in which $\hat{\phi}(\mathbf{s})$ is the set of estimated best responses of the adversary to the data subject's strategy $\mathbf{s}$. We refer to this type of Stackelberg games with a multistage re-identification attack as the MultiStage Re-Identification Game (MSRIG). The solution to the MSRIG (either the opt-in game or the masking game) can be found by calculating the payoffs of all data subject's strategies, as shown in Fig. S7B using a backward induction algorithm, which exhaustively

searches the space. To break a tie, we assume that the data subject prefers a strategy that shares more data and that the adversary prefers not to attack.

The critical component of the calculation is to obtain an adversary's estimated probability of an attack's success, $\hat{p}(\mathbf{s})$, by simulating the process of a multistage attack, whose running time is dependent upon the specific attack in consideration. This process may be time-consuming because a state-of-the-art attack model is typically very complex.

Note that the data subject's strategy space will become even larger if strategies such as generalization (i.e., replacing a value with the name of a group it is in) (*41*) are allowed. For example, if the value for age can be generalized to age groups according to a hierarchy of four levels: 35 → [30, 39] → [18, 39] → * (represents an age group that includes all ages), the strategy space will double in size. However, investigating these scenarios is out of the scope of this work.

D. Other scenarios

To demonstrate our game theoretic protection models' advantages, we designed two additional variations of the masking game and four baseline scenarios for comparison. In each variation of the masking game, an additional constraint is added to the scenario. In the first variation, we constrain the data subject to choose a strategy so that a rational adversary will never attempt an attack. Whereas in the second variation, we assume that the genetic genealogy dataset does not exist; thus the attack in the masking game has only one stage. In each baseline scenario, a data subject chooses a sharing strategy according to a different model. In the first two of them, the data subject always chooses a fixed sharing strategy. Whereas in the next two of them, the data subject randomly selects a sharing strategy. These scenarios are explained below in further detail with the introduction of their purposes.

In the no-attack masking game, we assume that the data subject is not allowed to choose any strategy that will make a rational adversary (driven by economic incentives) attack. To implement this game variation, we recorded the optimal strategy within the set of searched strategies which lead to no attack. The purpose of this scenario is to examine the maximal benefit that the data subject can get while ensuring full protection of his or her privacy.

In the one-stage masking game, we assume the genetic genealogy dataset does not exist in the game model. In other words, the re-identification attack only has one stage (i.e., direct linkage without inference). To implement this game variation, we forced the adversary in the game use no surname in the linkage stage. The purpose of this scenario is to test whether and how stage I (i.e., the inference stage) of the two-stage re-identification attack is essential for the attack.

In the no-protection scenario, the data subject always shares the entire data record. The purpose of this scenario is to test the maximal power of the two-stage re-identification attack.

In the demographics-only scenario, the data subject always shares only demographic attributes in the data record. The purpose of this scenario is to show the maximal power of the one-stage re-identification attack (i.e., without the inference stage).

In the random opt-in scenario, the data subject randomly decides to opt-in to share the entire record with a probability of 0.05. This probability is set according to the participation rate of GEDmatch, an online DNA comparison service provider, from customers of two major DTC-GT companies (i.e., 23andMe and AncestryDNA). GEDmatch has about 1.2 million users, and 23andMe and AncestryDNA has about 25 million customers in total by the end of 2019. This scenario aims to simulate how people are sharing targeted datasets in the real world.

In the random masking scenario, the data subject randomly decides to share each attribute in the record with a probability of 0.15. The expected privacy for each data subject in the last baseline scenario is almost the same as the optimal one in the masking game scenario. The last baseline scenario's purpose is to show an approximate lower bound on the expected utility for a data subject who shares partial data.

Supplementary Note S4. Search approaches to solving the masking game.

Here, we introduce approaches to searching for the optimal strategy in our game theoretic models. It is computationally challenging to search for the optimal strategy in the masking game because the strategy space expands exponentially as the number of attributes *m* increases. The backward induction algorithm, a brute force algorithm, could work well when *m* is relatively small. However, when *m* is relatively large, pruning techniques should be used to reduce the search space. Furthermore, heuristics could be used to find a locally optimal strategy quickly.

The strategy space for a data subject in a masking game could be represented in a lattice structure, as shown in Fig. S8. There are 2^m strategies to share a record with *m* attributes. That is to say, the lattice that represents the strategy space of sharing a record with 20 attributes has more than one million nodes. In the lattice, each strategy node represents a simulation of a complete two-stage attack. In the Gymrek attack, the adversary queries the genetic genealogy database of hundreds of thousands of records in the inference stage before querying the identified database of millions of records in the linkage stage. According to Gymrek *et al.*, hours of manual investigation is required for one successful attack in practice (*18*). Even performing a demonstration attack or a simulated attack is time-consuming.

To accelerate the computation, we can search only part of the strategy space to accelerate the search process instead of search exhaustively. Furthermore, we equipped our program with several algorithms and heuristics and saved intermediate results in memory to prevent repeated calculations.

The game-solving system was implemented in Python 3.8.5 using several machine learning libraries (e.g., Numpy 1.19.1, Scikit-learn 0.23.2, Pandas 1.1.3, Matplotlib 3.3.1, Seaborn 0.11.0, and SciPy 1.5.2) managed by Anaconda3. All source code and all datasets in our experiments are accessible from https://github.com/zhywan/msrigs (archived at https://doi.org/10.5281/zenodo.5543369).

In our experiments based on large-scale simulated datasets, by using a brute-force algorithm, searching for the best strategy for each data subject in each run of masking game took, on average, 11.5607 seconds on a machine with a six-core 64-bit central processing unit (CPU) clocked at 4.19 GHz, and a 32-gigabyte random-access memory (RAM) clocked at 2400 MHz.

S4.1. Greedy algorithm

As illustrated by Fig. S8, the "child" of a sharing strategy *s* is defined as a strategy that shares one fewer attribute than the strategy *s* shares. As a result, the strategy at the bottom – sharing nothing – has no "children". A greedy algorithm searches the strategy space from top to bottom. It keeps searching the "children" of the current best strategy, calculating their payoffs, and continues with a "child" that has the highest payoff. When several "children" have the same highest payoff, a "child" with the highest privacy measure will be selected to continue the search. The search stops at the bottom of the lattice. This algorithm can only find a locally optimal strategy.

In our experiments, by using the greedy algorithm, we reduced the average running time for each data subject in each run of the masking game by 98.33% to 0.1932 seconds. The globally optimal strategies for most data subjects are found. The data subjects' average payoff using the greedy algorithm was almost the same as the one using the brute-force algorithm, with a negligible difference of 2.63%.

S4.2. Pruning technique

Note that both the benefit of sharing $b(\mathbf{s})$ and the adversary's estimated probability of successful attack $\hat{p}(\mathbf{s})$ are functions of the sharing strategy (s) and increase monotonically (or remain the same) along with the increasing amount of shared data. In other words, if the strategy $\mathbf{s}'$ is a "child" of the strategy $\mathbf{s}$, then $b(\mathbf{s}') \leq b(\mathbf{s})$ and $\hat{p}(\mathbf{s}') \leq \hat{p}(\mathbf{s})$. Thus, if the payoff of the strategy $\mathbf{s}$ is lower than the current highest payoff, and if the adversary's corresponding best action is $\hat{\phi}(\mathbf{s}) = \{0\}$ (i.e., the adversary decides not to attack), according to Fig. S8, we can infer that payoffs of all "offspring" of the strategy s are also lower than the current highest payoff. To explain our pruning process in more detail, without loss of generality, let us make the following assumptions:

1) The sharing strategy $\mathbf{s}$ is sharing the first 12 attributes from a total of 16 attributes.

2) One of its "children" $\mathbf{s}'$ is sharing the first 11 attributes.

3) One of its "grandchildren" $\mathbf{s}''$ is sharing the first 10 attributes.

4) The current highest payoff is $v^* = \$80$.

5) $b(\mathbf{s}) = \$75$.

6) The benefit of sharing each attribute is \$6.25.

7) $\hat{p}(\mathbf{s}) = 1$ (i.e., there is only one matched identified record for the targeted data subject).

To apply the pruning, with the condition that $\hat{\phi}(\mathbf{s}) = \{0\}$ and $v_d\left(\mathbf{s}, \hat{\phi}(\mathbf{s})\right) < v^*$, we need to show that $v_d\left(\mathbf{s}', \hat{\phi}(\mathbf{s}')\right) < v^*$ and $v_d\left(\mathbf{s}'', \hat{\phi}(\mathbf{s}'')\right) < v^*$. The demonstration process is as follows:

1) Because the "child" $\mathbf{s}'$ shares one fewer attribute than the strategy $\mathbf{s}$ shares, there can be more than one matched identified record for the targeted data subject, and thus $\hat{p}(\mathbf{s}') \leq \hat{p}(\mathbf{s})$.

2) Because the "grandchild" $\mathbf{s}''$ shares two fewer attributes, there can be even more matched identified records for the targeted data subject, and thus $\hat{p}(\mathbf{s}'') \leq \hat{p}(\mathbf{s})$.

3) Because $\hat{\phi}(\mathbf{s}) = \{0\}$, according to the definition of $\hat{\phi}(\mathbf{s}) = \left\{a | \underset{a}{\operatorname{argmax}} \left(L\hat{p}(\mathbf{s})a - Ca\right)\right\}$, we can infer $L\hat{p}(\mathbf{s}) - C \leq 0$.

4) Because $\hat{p}(\mathbf{s}'') \leq \hat{p}(\mathbf{s}') \leq \hat{p}(\mathbf{s})$, we have $L\hat{p}(\mathbf{s}'') - C \leq L\hat{p}(\mathbf{s}') - C \leq L\hat{p}(\mathbf{s}) - C \leq 0$, thus $\hat{\phi}(\mathbf{s}'') = \hat{\phi}(\mathbf{s}') = \hat{\phi}(\mathbf{s}) = \{0\}$.

5) Because $\mathbf{s}'$ shares one fewer attribute, we have $b(\mathbf{s}') = \$68.75$.

6) Because $\mathbf{s}''$ shares two fewer attributes, we have $b(\mathbf{s}'') = \$62.5$.

7) Because $\hat{\phi}(\mathbf{s}'') = \hat{\phi}(\mathbf{s}') = \hat{\phi}(\mathbf{s}) = \{0\}$, we have $v_d\left(\mathbf{s}'', \hat{\phi}(\mathbf{s}'')\right) = v_d(\mathbf{s}'', 0) = b(\mathbf{s}'')$, $v_d\left(\mathbf{s}', \hat{\phi}(\mathbf{s}')\right) = v_d(\mathbf{s}', 0) = b(\mathbf{s}')$, and $v_d\left(\mathbf{s}, \hat{\phi}(\mathbf{s})\right) = v_d(\mathbf{s}, 0) = b(\mathbf{s})$.

8) Because $b(\mathbf{s}'') \leq b(\mathbf{s}') \leq b(\mathbf{s})$, we can safely conclude that: $v_d\left(\mathbf{s}'', \hat{\phi}(\mathbf{s}'')\right) \leq v_d\left(\mathbf{s}', \hat{\phi}(\mathbf{s}')\right) \leq v_d\left(\mathbf{s}, \hat{\phi}(\mathbf{s})\right) < v^*$.

Thus, these "offspring" of the strategy *s* can be pruned from the search space. This pruning technique could be applied to both the brute force algorithm and the greedy algorithm to accelerate the search. When applied to the brute force algorithm, the globally optimal strategy could be found more quickly.

In our experiments, by applying this pruning technique, we reduced the average running time of the greedy algorithm by 51.55% to 0.0936 seconds, and we reduced the average running time of the brute force algorithm by 31.36% to 7.9353 seconds. We solved the masking games in the sensitivity and robustness analyses using the greedy algorithm with pruning to accelerate the computations.

Supplementary Note S5. Data sanitization process for Craig Venter's data and the Ysearch dataset.

To protect the privacy of the corresponding subjects and enable replications of our investigation, we sanitized (i.e., modified for privacy protection) the original Venter and Ysearch datasets before conducting the experiment, but without affecting the data usefulness for the demonstration purpose. Specifically, each non-missing value was substituted by a unique random number (within an attribute-dependent range) for all records regarding each genomic attribute. Additionally, we masked each genomic attribute's name and replaced each surname in the dataset (except the surname Venter) with a uniquely random artificial surname (again, except the surname Venter). At last, we shuffled rows and columns in these datasets.

Now we explain how the value substitution worked in more detail. For example, let us say that the value of Y-STR y_1 is within the set of {2, 4, 5, 6} for all records in the experiments, then a random substitution mapping for Y-STR y_1 could be {2→3, 4→2, 5→5, 6→4} for all records. In addition, let us say that the value of Y-STR y_2 is within the set of {7, 8, 9, 10} for all records, then a random substitution mapping for Y-STR y_2 could be {7→3, 8→6, 9→4, 10→5} for all records. Note that, we did not allow two original values to be substituted by the same value.

The name replacements worked in a similar way. For instance, let us say that the set of surnames in the dataset is {Smith, Johnson, Williams, Venter, Leonard}, then a random replacement mapping could be {Smith→Fisher, Johnson→Barnes, Williams→Butler, Venter→Venter, Leonard→Swanson}. Still, we did not allow two original surnames to be substituted by the same artificial surname.

In these ways, in addition to not releasing all attributes' names, we made it almost impossible for a data recipient to recover the original data and kept all the data usefulness to demonstrate the attack and to demonstrate our game theoretic protection. All the effectiveness measures of the experiment conducted on the original dataset are the same as those of the experiment conducted on the modified dataset.

Supplementary Note S6. Data preparation process in the experiments based on a large-scale simulated population.

We simulated a genetic genealogical population using an individual-based forward-time population genetics simulation tool, simuPOP 1.1.8.3 (v2017) (*110*, *111*) (simupop.sourceforge.net), from which we derived three datasets. The data preparation pipeline for datasets used in the experiments is shown in Fig. S9. The inputs of the population simulation engine include probability distributions and statistics of corresponding attributes. The distributions of surnames (*112*) and demographics such as states (*113*) are based upon the 2010 U.S. census data. From the census data, the top 1000 surnames were selected to generate the population. 16 Y-STR markers were selected according to a study on Y-STR mutations (*114*). The Y-STR statistics, including the distribution of tandem repeats and mutation rates, were queried in 2018 from the Y-Chromosome Haplotype Reference Database (YHRD) (www.yhrd.org) and summarized in Table S2.

The process of population generation using simuPOP was as follows. The population data were initialized with the following attributes: ID, father's ID, mother's ID, sex, subpopulation, year of birth, state of residence, surname, income level, socioeconomic level, and 16 Y-STRs. IDs are integers starting from 0. We set the Y-STRs, state of residence, surname, and income level for everyone in the first generation according to corresponding distributions, respectively. The distribution of income level (every $2500 per level) is based upon a survey (*115*) conducted in 2015 by the U.S. Census Bureau. The level of socioeconomic status (i.e., upper-class, middle-class, or lower-class) was set according to an individual's income level. The middle-class range was set to be [$40,500, $99,999] according to numbers (*116*) from the Pew Research Center and the U.S. Census Bureau. Everyone's year of birth was set uniformly at random from the 30-year range of [1670, 1699]. Each sex group has the same size, and each subpopulation has the same size as well.

Afterward, we set the mating scheme in the simulation engine that chooses parents from a prenatal generation and generates offspring from chosen parents. Parents were chosen from their respective sex groups. Instead of using a random chooser or any pre-defined chooser, we customized the chooser according to the following procedure. First, we selected an individual from the pool of candidates and record the socioeconomic level, and then we selected a pair of parents from this socioeconomic level uniformly at random. For those parents who do not meet a particular set of conditions, a new pair of parents will be re-selected if a random number generated from the range of (0, 1) is larger than 0.2. The selection loop ends if those conditions are met, or if the random number generated in the current loop is smaller than 0.2. The set of conditions include that the father is not two years younger than the mother, and that the father is not 12 years older than the mother, and that the father's income level is not lower than the mother's income level. A selected individual has a probability of 80% to be moved out of the candidate pool unless everyone else in the candidate pool has either different sex or different socioeconomic level from the selected individual.

For each pair of parents, the number of children was determined by a zero-truncated Poisson distribution with the parameter $\lambda = 1.6$, which has an expected value of $\lambda/\left(1 - e^{-\lambda}\right) = 2.0$ and a standard deviation of 1.4, according to the average number of own children in families

in the United States in 1977. Children's attributes were set as follows: The sex of each child was assigned randomly with equal probabilities of male and female. The genotypes of each child were transmitted from parents following Mendel's laws. And the surname of each child was inherited from the father. To simulate each mutation event, we used a stepwise mutation model (*117*) to increase or decrease the number of repeats for each locus of a new genomic sequence.

To simulate each birth event, we set the child's year of birth as the sum of the mother's year of birth and the mother's age during the birth event, the latter of which was generated based on a distribution of ages that women become mothers. According to a report from the U.S. National Center for Health Statistics (*118*), the average numbers of births for women ages 15–19, 20–24, 25–29, 30–34 are 0.18 (5%), 0.73 (19%), 1.10 (29%), 1.09 (29%) millions in 2018, respectively. However, if the father's resulting age is smaller than 16, the child's year of birth would be set as the father's year of birth plus 16. To simulate the change of socioeconomic level across generations, we assumed that the expected income level of an individual (in middle age) is equal to the rounded average income level of his or her parents plus a number selected from the set of [−1, 1] uniformly at random unless the resulting income level of the individual is out of bounds. To simulate the change of state of residence, we assumed a child's state of residence has a probability of 33.33% to be different from their parents' state of residence. The new state of residence was selected according to the corresponding distribution of state. Otherwise, the child would have a probability of 50% sharing the same state of residence with their father and the same probability sharing the same state of residence with their mother.

The simulation for the mating process for a prenatal generation in a subpopulation stopped when enough people are generated for the next generation. We assumed that every subpopulation has the same number of people in each generation before migration events. To simulate migration events among subpopulations, we used a migration-by-proportion model to migrate a fixed 10% proportion of each subpopulation to each other populations every five generations. The entire population generation process stopped when enough people are generated for the last generation. The family tree of a randomly selected family (the Leonard family) across the last three generations, with the corresponding primary attributes, is shown in Fig. S10. Note that the surname before marriage (i.e., maiden name) is shown for each female member in the family.

The generated population has a total of 600,000 records with 26 attributes and the family structures (i.e., pedigrees), from which we outputted a male population of 90,064 records (last three generations) with 20 primary attributes, including ID, surname, two demographic attributes (i.e., year of birth and state of residence) and 16 genomic attributes (i.e., 16 Y-STRs).

In each experiment, we randomly selected three datasets from the population. We first selected 20,000 records uniformly at random and kept a set of attributes (namely, ID, first name (randomly generated), surname, year of birth, and state of residence) as the identified dataset D_I. Then we selected 1,000 records uniformly at random from these 20,000 records in D_I and kept another set of attributes (namely, ID, surname, year of birth, state of residence, and Y-STRs) as the targeted genomic dataset D. Note that the surname attribute in this simulated dataset will not be released because it rarely exists in a real-world public dataset. Last, we selected 20,000 records uniformly at random from the population, excluding these 20,000 records in D_I, and kept

a set of attributes (namely, ID, surname, Y-STRs) as the genetic genealogy dataset D_G. We noticed that a real-world genetic genealogy dataset D_G (e.g., Ysearch) contains many missing values, so we let a specific portion (e.g., 30%) of genomic values in this dataset be missing values.

Supplementary Note S7. Usefulness of the dataset shared by a pool of data subjects using simulated datasets.

In both sets of experiments, as well as in the sensitivity analysis and the robustness analysis, we defined a data subject's utility as the benefit of sharing divided by the maximal benefit of sharing all data. In the experiments, we used the average utility of a pool of data subjects as an effectiveness measure which mainly measures a data sharing strategy's ability to help data subjects gain benefit. The utility for a dataset consisted of the shared records of a pool of data subjects has never been explicitly defined. However, inherently, it is defined as the average utility of the pool of data subjects contributing data to the dataset. This definition focuses on the data volume instead of the data quality and has three characteristics: (1) It does not depend upon the usage of the shared dataset; (2) It is additive across data records; and (3) It can treat each attribute differently.

Yet, due to the potential for missing values in the shared data, the usefulness (or the data quality) of the dataset shared by a pool of data subjects should be measured as well. Although the utility metric we applied (Eq. S3 in note S2) to measure effectiveness of a sharing strategy has been used to measure the data quality of anonymized datasets in prior literature (*41*, *103*, *104*), the distance between the distribution of the original data and the distribution of released data is recommended to measure the data quality of synthetic datasets (*119*). Alternatively, the usefulness of a released dataset can be defined as: (i) the difference between summary statistics (e.g., mean, standard deviation, and correlation matrix) or (ii) the difference between test (e.g., regression, classification, and clustering) results based on unprotected and released data. However, because we assume that no dependent attributes exist in the large-scale simulated datasets and we assume no specific use cases, we define the usefulness metric based on a general distribution-based distance measure – variational distance (VD) (*51*). Compared to KL-divergence (another distance measure) (*51*), VD is a metric bounded in the range of [0, 1]. The VD between two distributions is defined as in Eq. S17:

$$d(\mathbf{H}',\mathbf{H}) = \frac{1}{2}\sum_{i=1}^{m_v}|h_i' - h_i| \tag{S17}$$

where $\mathbf{H}'$ and $\mathbf{H}$ are two sets of data points for a same attribute, in which h_i' and h_i are the relative frequencies corresponding to the ith distinct value ($i = 1,\cdots,m_v$). In cases where $\mathbf{H}'$ or $\mathbf{H}$ is an empty set, the VD can be set to its maximal value of one. Last, we define the usefulness of a released targeted dataset D', given the original targeted dataset D, as the weighted sum of one minus the VD for each attribute, as shown in Eq. S18:

$$W(D',D) = \frac{1}{\sum_{j=1}^{m} w_j}\sum_{j=1}^{m} w_j\left(1 - d\left(\mathbf{D}_j',\mathbf{D}_j\right)\right) \tag{S18}$$

in which m is the number of attributes, w_j is the weight for the jth attribute of dataset D, $\mathbf{D}_j'$ is the set of data points in the jth attribute of dataset D', and $\mathbf{D}_j$ is the set of data points in the jth

attribute of dataset *D*. Weight w_j is set according to the jth attribute's corresponding information entropy in either the genetic genealogy dataset D_G or the identified dataset D_I.

We compare the game scenarios with two baseline scenarios: (i) random masking and (ii) k-anonymity. Note that k-anonymity has been utilized to protect individual-level genomic records in the literature (*67*). The setting for the random masking scenario is introduced in note S3. In the k-anonymity scenario, a data sharer for a pool of data subjects chooses a sharing strategy to k-anonymize the shared data by using the data anonymization tool – ARX (*58*), instead of trying to maximize each data subject's payoff. We set parameter k in the k-anonymity model (*37*) to 2, set the utility measure to precision model (*103*, *49*), set the transformation model to local transformation, set the height for all generalization hierarchies to 2, and use the default settings for all other parameters. The source code for implementing this scenario, with the help of the application programming interface (API) provided in ARX (version 3.9.0), is accessible from https://github.com/zhywan/msrigs (archived at https://doi.org/10.5281/zenodo.5543369). All settings for the opt-in game and the masking game are the same as those in the main experiment, except that a new setup for the masking game with a minority-support factor of one is added to the experiment. Table S3 reports on the usefulness measures for each scenario averaged across 100 runs, as well as the last step of the calculation (one minus the VD for each attribute). From Table S3, it can be seen that masking game with a minority-support factor of one performs better than other scenarios in terms of the usefulness measure. The masking game with a minority-support factor of zero, although masked heavily on an attribute with relative high weight, still outperforms all the baseline scenarios and the opt-in game.

Fig. S1. depicts a scatterplot of the data subjects' average privacy and the shared dataset's usefulness in each of the five scenarios averaged across 100 runs. From Fig. S1, it can be seen that masking game with a minority-support factor of one not only achieves the highest usefulness but also the highest privacy. The masking game with a minority-support factor of zero performs better than all the baseline scenarios and the opt-in game in terms of both privacy and usefulness. The random masking scenario, which achieves the same level of privacy with the masking game with a minority-support factor of zero, performs worse than all the other scenarios in comparison in terms of usefulness mainly because the very low amount of shared data. We note that the data sharing rate is 15% in the random masking scenario.

We also compare these three scenarios based on some important summary statistics to show how representative the selected usefulness measure is. These summary statistics include: (i) data sharing rate, (ii) number of distinct values, (iii) information entropy, (iv) Spearman correlation coefficients, (v) arithmetic mean, (vi) standard deviation, and (vii) skewness. The relative differences between released and unprotected data in terms of each of the first four summary statistics in five scenarios (with the specific summary statistics in the no-protection scenario presented), averaged across 100 runs, are shown in Tables S4 to S7, respectively. Specifically, the relative differences between a statistical value in the released data, denoted A', and its counterpart in the unprotected data, denoted A, is defined as $|A' - A|/A$. The absolute differences between for numerical attributes in released and unprotected data in terms of each of the last three summary statistics in five scenarios (with the specific summary statistics in the no-protection scenario presented), averaged across 100 runs, are shown in Tables S8 to S10, respectively.

On the basis of the results shown in Tables S4 to S10, there are several notable findings. First, in terms of each of these eight summary statistics, the masking game with a minority-support factor of one performs better than both baselines and the opt-in game. Second, the masking game with a minority-support factor of zero performs better than both baselines and the opt-in game in most of these summary statistics save the entropy. And, third, the rank orders of these scenarios according to these summary statistics are in accordance with their rank order according to the usefulness metric, which support the representativeness of the usefulness metric.

Supplementary Note S8. Fairness of data sharing using simulated datasets.

Subjects will be more or less likely to mask their data based on demographic attributes, which can lead to underrepresentation bias for those groups. For example, if African Americans face higher re-identification risks, they will mask data at higher rates. This could lead to negative downstream effects for such underrepresented groups.

It is important to measure the fairness of a data sharing policy that quantifies the similarity of all subgroups in a dataset in terms of a specific function (e.g., data usefulness, data utility, privacy, payoff, or data sharing rate). Given that our data do not explicitly include the race or ethnicity of the corresponding individuals, let us consider an example with respect to U.S. state of residence. For example, the fairness measure in terms of sharing rate would be quite low if residents of Maine share only 20% of their data, on average, while residents of California share 80% of their data, on average.

Here, we only consider the fairness between demographic groups. Note that there are only two demographic attributes (namely, year of birth and state of residence) in the simulated datasets. Before we introduce the definitions for the set of fairness measures, we point out that defining the fairness with respect to usefulness and defining other types of fairness are slightly different. Basically, when measuring the fairness with respect to usefulness, we only consider those data subjects who share the value of the demographic attribute, whereas when measuring other types of fairness (e.g., the fairness with respect to privacy), we consider all data subjects. We define the fairness for a demographic attribute as one minus Gini coefficient of a measure based on the grouping hierarchy (as shown in Fig. S6) of the attribute, and we further define the fairness of a released dataset, given the original dataset, as the average of fairness for each demographic attribute in this dataset. Compared to a standard deviation, a Gini coefficient is always bounded in the range between zero and one. For example, the fairness with respect to usefulness of a released dataset D', given the original dataset D and the grouping hierarchies can be defined as shown in Eq. S19:

$$F_W(D',D) = \frac{1}{m_d}\sum_{j=1}^{m_d}\left(1 - G\left(\left\{W\left(D'_{ij,*}, D_{ij,*}\right) | \forall i \in \left[1, n_{d,j}\right]\right\}\right)\right)$$

$$= \frac{1}{m_d}\sum_{j=1}^{m_d}\left(1 - G\left(\left\{\frac{1}{\sum_{k=1}^{m} w_k}\sum_{k=1}^{m} w_k\left(1 - d\left(\mathbf{D}'_{ijk}, \mathbf{D}_{ijk}\right)\right) | \forall i \in \left[1, n_{d,j}\right]\right\}\right)\right) \quad \text{(S19)}$$

in which m_d is the number of demographic attributes in dataset D, $G(\cdot)$ is the function for calculating Gini coefficient on a set of numbers, $W(D',D)$ is the function for calculating usefulness measure given two datasets, $n_{d,j}$ is the number of groups in the jth demographic attribute in dataset D, $D'_{ij,*}$ is the subset of dataset D' consists of all data subjects in the ith group of the jth demographic attribute in dataset D', $D_{ij,*}$ is the subset of dataset D consists of all data subjects in the ith group of the jth demographic attribute in dataset D, $\mathbf{D}'_{ijk}$ is the set of data points

of the kth attribute in the ith group of the jth demographic attribute in dataset D', and $\mathbf{D}_{ijk}$ is the set of data points of the kth attribute in the ith group of the jth demographic attribute in dataset D. In addition, as in Eq. S18, m is the number of attributes, w_k is the weight for the kth attribute, which is set according to the kth attribute's corresponding information entropy in either the genetic genealogy dataset D_G or the identified dataset D_I.

For each measure (e.g., payoff, utility, payoff, sharing rate) that is defined in an additive manner across records in a released dataset D, the corresponding fairness is also defined in an additive manner, as shown in Eq. S20:

$$F_f(D', D) = \frac{1}{m_d} \sum_{j=1}^{m_d} \left(1 - G\left(\left\{ \frac{1}{n_{g,ij}} \sum_{k=1}^{n_{g,ij}} f_{kij} \,|\forall i \in [1, n_{d,j}] \right\} \right) \right) \tag{S20}$$

in which $n_{g,ij}$ is the size of the ith group of the jth attribute in dataset D, and f_{kij} is the measure for the kth data subject in the ith group of the jth attribute in dataset D'. Given a data subject's sharing/masking strategy $\mathbf{s} = <s_1, \cdots, s_j, \cdots, s_m> \in \mathbb{B}^m$, the corresponding sharing rate is defined as in Eq. S21:

$$\rho(\mathbf{s}) = \frac{1}{m} \sum_{j=1}^{m} s_j \tag{S21}$$

The function for calculating Gini coefficient on a set of numbers $\mathbf{x} = <x_1, \cdots, x_i, \cdots, x_n>$, that are placed in ascending order, can be represented as Eq. S22:

$$G(\mathbf{x}) = \frac{\sum_{i=1}^{n} (2i - n - 1) x_i}{n \sum_{i=1}^{n} x_i} \tag{S22}$$

We compare the masking game scenario with two baseline scenarios: (i) random masking and (ii) k-anonymity, as we did in the analysis on the usefulness of the dataset in note S7. The settings for these two scenarios are introduced in note S7. All settings for the opt-in game and the two masking games are the same as those in the experiment of note S7. Table S11 reports on the fairness measures averaged across 100 runs in terms of usefulness, privacy, utility, payoff, or sharing rate, for six scenarios. From Table S11, it can be seen that the masking game with a minority-support factor of one performs better than the baseline scenarios and the opt-in game in term of all examined fairness measures. The masking game with a minority-support factor of zero outperforms all other scenarios in terms of almost all examined fairness measures except the fairness with respect to usefulness.

Fig. S2. depicts a scatterplot of the fairness with respect to privacy and fairness with respect to usefulness of the shared dataset in each of the five scenarios averaged across 100 runs. From Fig. S2, it can be seen that masking game with a minority-support factor of one performs better than all other scenarios save the no-protection scenario in terms of the fairness with respect to

usefulness and that it performs better than all other scenarios in terms of the fairness with respect to privacy. The masking game with a minority-support factor of zero performs better than the baseline scenarios and the opt-in game in terms of the fairness with respect to privacy, and it performs better than the *k*-anonymity scenario in terms of the fairness with respect to usefulness.

Supplementary Note S9. Sensitivity analysis on parameters and settings using simulated datasets.

To test the model's sensitivity to eight parameters and three experimental settings, we compared effectiveness measures in eight scenarios with one parameter or setting changed using a targeted dataset of 1000 data subjects in 20 runs of experiments. Sensitivity analysis of more parameters and settings could be potentially conducted in the same way. The default values for parameters for the experiments are provided in Table S1.

S9.1. Sensitivity analysis on parameters using simulated datasets regarding payoff

The sensitivity analysis results on eight parameters regarding the data subjects' average payoff are shown in the upper body of Fig. 4 (in the main text). In general, regardless of how the targeted parameter varies, the data subjects' average payoff in the masking game is much higher than their average payoff in other scenarios (except two variations of the masking game). Specifically, the one-stage variation brings more average payoff, and the no-attack variation guarantees full privacy protection.

A. Number of genomic attributes

Fig. 4A shows how the data subjects' average payoff changes as the number of genomic attributes on the Y chromosome varies from 2 to 16. To ensure comparability, we fixed the maximal benefit of sharing all data B in this experiment. First, we can observe in Fig. 4A that there is little change in the data subjects' average payoff in the masking game (less than 5% of the maximal change as in the no-protection scenario), which indicates that the data subjects' average payoff is not sensitive to the number of genomic attributes in the masking game. Second, the data subjects' average payoff decreases substantially as the number of genomic attributes increases in the no-protection scenario, the demographics-only scenario, and the opt-in game. This trend occurs because the accuracy of the surname-inference attack increases as more genomic data are shared (for all scenarios except the demographics-only scenario and the one-stage masking game). For the demographics-only scenario, the underlying reason is that the utility of the shared demographic data decreases as more genomic data are shared. Third, the difference between the opt-in game and the no-protection scenario in terms of the data subjects' average payoff decreases as the number of genomic attributes increases. This difference exists because the data subject has a greater chance to opt-out when more genomic attributes are available in the opt-in game.

B. Proportion of missing genomic data

The genetic genealogy dataset D_G may have missing values (or missing data), especially in real-world cases. For example, 26% values for genomic attributes are missing values in the Ysearch dataset (after filtering out records with too few Y-STR markers). Note that all data subjects' surnames are included in the dataset. We denoted by γ the proportion of missing genomic data in dataset D_G. Fig. 4B shows how the data subjects' average payoff changes as the proportion of missing genomic data in dataset D_G varies from 0 to 0.9 (in increments of 0.1). It can be seen that there is little change in the data subjects' average payoff in the masking game

(less than 14% of the maximal change as in the no-protection scenario). Moreover, the data subjects' average payoff increases as the missing proportion increases in all scenarios save the random masking scenario and the one-stage masking game. This trend occurs because data missing from dataset D_G can be considered as the result of a randomized masking strategy, which reduces the privacy risk but does not affect data utility.

C. Threshold for confidence score

In the Gymrek attack, a confidence score is used to measure the adversary's confidence that an attack's surname-inference stage will be successful. A threshold for confidence score is required for an adversary to make the attack decision. Fig. 4C shows how the data subjects' average payoff changes when the confidence score threshold varies from 0 to 1 (in increments of 0.1). It can be seen that there is little change in the data subjects' average payoff in the masking game (less than 15% of the maximal change as in the no-protection scenario). In addition, the data subjects' average payoff increases as the threshold increases in all scenarios save the demographics-only scenario, the random masking scenario, and the one-stage masking game. This trend occurs because the adversary is unlikely to infer a surname with a high threshold for confidence score, making the attack less successful.

D. Number of records in the genetic genealogy dataset

Fig. 4D shows how the data subjects' average payoff changes as the number of records in the genetic genealogy dataset D_G is varied from 2,000 to 40,000 (in increments of 2,000). It can be seen that there is little change in the data subjects' average payoff in the masking game (less than 6% of the maximal change as in the no-protection scenario). It should also be recognized that the data subjects' average payoff decreases as the number of records in the genetic genealogy dataset increases in the no-protection scenario, the opt-in game, the masking game and the no-attack masking game. This trend occurs because the adversary is more likely to find someone sharing similar genomes with the targeted data subject in a larger genetic genealogy dataset, making the attack more likely to be successful.

E. Number of records in the identified dataset

We assumed the adversary in the re-identification attack links the targeted data subject to only one matched identified record, following the marketer re-identification risk model (*109*). As a result, a larger identified dataset increases the expected number of matched identified records and, thus, reduces the probability of the attack's success for each targeted data subject. Fig. 4E shows how the data subjects' average payoff changes as the number of records in identified dataset D_I is varied from 2,000 to 40,000 (in increments of 2,000). It can be seen that there is little change in the data subjects' average payoff in the masking game (less than 12% of the maximal change as in the demographics-only scenario). In addition, the data subjects' average payoff increases as the number of records in the identified dataset increases in all scenarios save the random opt-in scenario and the random masking scenario. This trend occurs because a larger identified dataset reduces the likelihood of the attack's success. Thus, when a data subject can choose to share data, they will be more likely to do so if the identified dataset has more records than not.

F. Loss from being re-identified

Fig. 4F shows how the data subjects' average payoff changes when the financial loss associated with being re-identified varies from 0 to $400 (in increments of $25). Note that we assumed that the adversary's gain from re-identification is always equal to the data subject's loss from being re-identified. It can be seen that, in all baseline scenarios, the data subjects' average payoff decreases linearly as the loss from re-identification increases. Further, in the opt-in game, the data subjects' average payoff keeps decreasing linearly until the loss reaches $100, which is equal to the maximal benefit of sharing all data. This trend manifests for two reasons. First, in this game, the data subject always chooses to share data until their loss surpasses the benefit of sharing data. Second, an adversary is not able to attack the data subjects whose data is not shared. Note that, in the masking game (and two of its variations), the data subjects' average payoff decreases as the loss from being re-identified increases. This trend occurs because the loss indicates that the impact of a successful attack on a data subject is negative. However, the curve is relatively flat (the range of change in the masking game is less than 7% of the maximal change as in the no-protection scenario), which implies that the protection strategy works well.

G. Maximal benefit of sharing all data

Fig. 4G shows how the data subjects' average payoff changes when the maximal benefit of sharing all data varies from 0 to $400 (in increments of $25). It can be seen that in all scenarios (except the opt-in game), the data subjects' average payoff linearly increases as the maximal benefit of sharing all data increases. This trend occurs because the data subjects' average payoff is a positively correlated linear function of the maximal benefit of sharing all data, which is unavoidable. In addition, in the opt-in game, the data subjects' average payoff starts to linearly increase after the maximal benefit of sharing all data surpasses $150, which is equal to the loss from being re-identified. This trend occurs because a data subject tends to share data if their benefit surpasses the loss from sharing data.

H. Cost of an attack

Fig. 4H shows how the data subjects' average payoff changes when the adversary's cost to execute an attack varies from 0 to $160 (in increments of $10). It can be seen that, in all scenarios (except the random masking scenario), the data subjects' average payoff increases as the cost of an attack increases. This trend implies that, in addition to data masking, raising an adversary's cost (e.g., penalizing the adversary for privacy breach) can effectively deter adversaries' attacks. However, the curve is relatively flat (the range of change in the masking game is less than 17% of the maximal change as in the no-protection scenario), which implies that the protection strategy works well.

<u>S9.2. Sensitivity analysis on settings using simulated datasets regarding payoff</u>

The sensitivity analysis results on the three experimental settings (assumptions) regarding the data subjects' average payoff are shown at the bottom of Fig. 4 (in the main text). In general, regardless of how the targeted experimental setting (assumption) varies, the data subjects'

average payoff in the masking game is much higher than their average payoff in other scenarios (except the no-attack masking game).

I. Weight distribution of attributes

In the masking game, each attribute in the targeted dataset has a certain weight in calculating the data utility. In the current setting, an attribute's weight is proportional to its information entropy in either the genetic genealogy dataset or the identified dataset. In this experiment, we examined two alternative settings of weight distribution to investigate the degree to which the weight distribution affects a data subject's optimal strategy and payoff. In the more balanced setting, every attribute has the same weight, while in the more biased setting, the weight of the first two genomic attributes is ten times the weight of all other attributes (without loss of generality). Fig. 4I shows a violin plot that shows how the weight distribution of attributes changes the data subjects' average payoff in the masking game. It can be seen that, in the masking game (and its no-attack variation), the data subjects' average payoff will change if the weights of attributes are changed. Specifically, the further the attributes' weights are set away from their information entropies, the higher the average payoff the data subjects can obtain. This trend occurs because the attributes in a dataset that contain more information than others can be used to uniquely identify a record more easily. In other words, if any of these attributes are assigned with a weight that is smaller than its entropy, it is more likely to be masked without reducing too much utility but reducing the privacy risk substantially, and thus raises the data subjects' average payoff.

J. Homogeneity constraint for adopted strategies

In certain situations, a group of data subjects is required to adopt the same strategy. These situations happen when a data sharer chooses the same sharing strategy for a group of data subjects, or when those data subjects in a group make decisions interdependently. Fig. 4J shows a violin plot that shows how the homogeneity of adopted strategies influences the data subjects' average payoff in three games. It can be seen that, in all games, the data subjects' average payoff will decrease if those data subjects in a dataset are required to adopt the same strategy. In other words, the data subjects need to trade their average payoffs for the homogeneity of adopted strategies. By comparing the masking game with the opt-in game, it can be seen that the data subjects' average payoff with the homogeneity constraint is still relatively high in the masking game, but it is always as low as zero in the opt-in game. This difference may be caused by the different flexibilities of these two games.

K. Surname inference approach

The adversary's approach to inferring surnames affects the accuracy of the inference and, thus, affects the data subjects' payoffs. In the Gymrek attack, a surname is inferred by finding the nearest neighbor with the time to most recent common ancestor (TMRCA) as the distance measure. However, the surname associated with a genomic record could be inferred using off-the-shelf machine learning approaches as well, and the accuracy of the inference might be acceptable. In the context of machine learning, a genealogy dataset can be treated as a training set in which surnames are labels. After applying two machine learning approaches (namely, *k*-

nearest neighbors (KNN) and linear regression) to the surname inference, we tested our protection methods against the two-stage attack in which a surname is inferred using one of these approaches. Fig. 4K shows a violin plot that shows how the data subjects' average payoff changes according to the surname inference approach. First, we calibrated the parameters in these two machine learning models. Afterward, we set the confidence score to 1 for all compared approaches to ensure comparability. It can be seen that, for each game, attacks based on machine learning approaches result in higher average payoff for data subjects compared with the original surname inference approach, in which the KNN approach works better for the adversary (i.e., brings lower average payoff to the data subjects). This is because these off-the-shelf machine learning approaches are not customized to the surname inference problem, and the KNN approach has fewer parameters and thus is easier to be calibrated.

S9.3. Sensitivity analysis on parameters using simulated datasets regarding privacy and utility

The sensitivity analysis results on eight parameters regarding the data subjects' average privacy and average utility are shown in Figs. 5 and 6, respectively. In general, in all experiments, the data subjects' average privacy in the masking game is almost always higher than their average privacy in other scenarios except two variations of the masking game. In addition, the data subjects' average utility in the masking game is only lower than their average utility in the no-protection scenario and the one-stage masking game.

A. Number of genomic attributes

From Figs. 5A and 6A, it can be seen that changes in data subjects' average privacy and average utility in the masking game are mild. Notably, privacy increases (and utility decreases) monotonically as the number of genomic attributes in the opt-in game increases. In addition, as the number of genomic attributes increases, privacy increases (and utility decreases) in the demographics-only scenario. Moreover, as the number of genomic attributes increases, the utility remains unchanged in the random opt-in and random masking scenarios. Last, in the no-protection scenario, the utility does not change (and privacy decreases drastically) as the number of genomic attributes increases. It is because the accuracy of a surname-inference attack increases with more genomic data being shared.

B. Proportion of missing genomic data

From Fig. 5B, it can be seen that the data subjects' average privacy in the masking game remains almost unchanged when the proportion of missing data changes. By contrast, in the no-protection scenario, as the proportion of missing data increases, the data subjects' average privacy increases monotonically because the surname inference becomes less accurate in this situation. However, in the opt-in game, the data subjects' average privacy decreases as the proportion of missing data increases. At the same time, from Fig. 6B, it can be seen that the data subjects' average utility increases monotonically as the proportion of missing data increases in both opt-in and masking games. This trend implies that more data should be shared if the adversary has access to less external data.

C. Threshold for confidence score

From Fig. 5C, it can be seen that the data subjects' average privacy in the masking game remains unchanged when the threshold for confidence score changes. By contrast, in the no-protection scenario, as the threshold for confidence score increases, the data subjects' average privacy increases monotonically. It is because fewer surnames can be inferred in this situation. However, in the opt-in game, the data subjects' average privacy decreases as the threshold for confidence score increases. At the same time, from Fig. 6C, it can be seen that the data subjects' average utility increases monotonically as the threshold for confidence score increases in both opt-in and masking games. This trend implies that more data should be shared if the adversary is more conservative on attacks.

D. Number of records in the genetic genealogy dataset

From Fig. 5D, it can be seen that the data subjects' average privacy in the masking game remains unchanged when the number of records in the genetic genealogy dataset changes. By contrast, in the no-protection scenario, as the number of records in the genetic genealogy dataset increases, the data subjects' average privacy decreases monotonically. It is because more surnames can be inferred correctly. However, in the opt-in game, the data subjects' average privacy increases as the number of records in the genetic genealogy dataset increases. At the same time, from Fig. 6D, it can be seen that the data subjects' average utility decreases monotonically as the number of records in the genetic genealogy dataset increases in both opt-in and masking games. This trend implies that less data should be shared if the adversary has access to a larger genetic genealogy dataset.

E. Number of records in the identified dataset

From Fig. 5E, it can be seen that the data subjects' average privacy in all games remains almost unchanged when the number of records in the identified dataset changes. By contrast, in the no-protection scenario and the demographics-only scenario, as the number of records in the identified dataset increases, the data subjects' average privacy increases monotonically because more identities can be linked to each target. At the same time, from Fig. 6E, it can be seen that the data subjects' average utility increases monotonically as the number of records in the identified dataset increases in all games. This trend implies that more data should be shared if the adversary has access to a larger identified dataset.

F. Loss from being re-identified

From Figs. 5F and 6F, it can be seen that the data subjects' average privacy, in all baseline scenarios instead of four game scenarios, remains almost unchanged when the loss is greater than \$25. It is because the cost of an attack is too low (\$10) to let the loss from being re-identified affect the effectiveness of the attack if the loss is greater than the cost. By contrast, in the opt-in game, although utility does not increase (and privacy does not decrease) as the loss increases when the loss is greater than \$25, both utility and privacy change dramatically when the loss reaches \$100, which is equal to the maximal benefit of sharing all data. Similarly, in the masking game (and its no-attack variation), privacy increases (and utility decreases) substantially until the loss is greater than \$100.

G. Maximal benefit of sharing all data

From Figs. 5G and 6G, it can be seen that the data subjects' average privacy and utility, in all baseline scenarios instead of four game scenarios, almost remain the same because the effectiveness of attack is not related to the maximal benefit of sharing all data. By contrast, in the opt-in game, although privacy does not increase (and utility does not decrease) as the benefit increases, utility and privacy drastically change when benefit surpasses \$150, which is equal to the loss from being re-identified. However, in the masking game (and its one-stage variation), privacy slowly decreases (and utility slowly increases) when the maximal benefit of sharing all data is greater than \$25.

H. Cost of an attack

From Figs. 5H and 6H, it can be seen that, in all scenarios, privacy increases (and utility does not decrease) as the cost of an attack increases. In addition, they reach the maximal value when the cost of an attack reaches \$150, which is equal to the maximal gain of the adversary from re-identification (i.e., the data subject's loss from being re-identified). This is because the increase in the cost of an attack disincentivizes an adversary to attack and does not negatively impact the data utility. This suggests that alternative deterrents (e.g., penalties in data use agreements), if invoked, can increase the cost of an attack and so mitigate the privacy risk as well.

Supplementary Note S10. Robustness analysis on uncertainty in parameters using simulated datasets.

In both sets of experiments, including the sensitivity analysis, we assumed that the data subject always correctly estimates the expected payoff and the probability of an attack's success for any strategy. However, in practice, the data subject is likely to be uncertain about the value of some parameter in the game model. As a result, it is possible that they could overestimate the payoff and/or underestimate the probability of an attack's success. In this section, we conduct a robustness analysis on (i) the adversary's cost of an attack, (ii) the number of records in the genetic genealogy dataset, and (iii) the number of records in the identified dataset, respectively, to see how the uncertainty in the data subject's knowledge about the adversary affects the data subject's expected payoff and the probability of an attack's success.

<u>S10.1. Cost of an attack</u>

First, we investigated how bias in the data subject's knowledge on the adversary's cost of an attack affects the data subject's expected payoff and the probability of an attack's success. Figs. S3A, S3B and S3C depicts the average probability of an attack's success, the average privacy, and the average payoff, respectively, in a pool of data subjects as the actual cost of an attack varies from \$0 to \$160 (in increments of \$10) in ten scenarios. We compared two game scenarios (i.e., the opt-in game and the masking game) with two baseline scenarios (i.e., the no-protection scenario and the random masking scenario). For each game scenario, we considered four sub-scenarios in each of which the data subject believes the cost of an attack is fixed at \$10, \$20, or \$30, or knows the actual cost. All of the other experimental settings are the same as those in the sensitivity analysis on parameters using simulated dataset (see note S9 for details).

In Figs. S3A, S3B, and S3C, it can be seen that the average probability of an attack's success, the average privacy, and average payoff does not decrease as the actual cost of an attack increases in all scenarios. In Fig. S3C, it can be seen that, although the masking game scenario is sub-optimal in terms of the data subjects' average payoff when all data subjects are uncertain about the actual cost of an attack, it still substantially outperforms the opt-in game scenarios and baseline scenarios in most cases in terms of the payoff, especially when the estimated value of the parameter is close to the actual one.

In Figs. S3A, S3B, and S3C, it can be seen that, for each estimated value of the cost of an attack, the greater its overestimation, the greater the overestimation of the payoff. For each actual value of the cost of an attack, the closer the estimated value is, the closer the estimated payoff will be to the optimal payoff. Furthermore, the data subject's uncertain knowledge about the cost of an attack will not affect the estimate on the probability of an attack's success. When the bias in the number of records in the identified dataset is bounded within the range of 100% (e.g., \$10 in \$10), the estimation error of the expected payoff is bounded within the range of around \$3 (e.g., 4% of \$83). Regardless of how the actual cost of an attack change, the estimation error of the expected payoff will be bounded within the range of around \$3 (i.e., 4% of the optimal payoff), which is still substantially smaller than \$63 (i.e., the difference between the results of the masking game and the opt-in game).

S10.2. Number of records in the genetic genealogy dataset

Next, we investigated how bias in the data subject's knowledge about the number of records in the genetic genealogy dataset affects the data subject's expected payoff and the probability of an attack's success. Figs. S3D, S3E, and S3F depict the average probability of an attack's success, the average privacy, and the average payoff, respectively, in a pool of data subjects as the actual number of records in the genetic genealogy dataset varies from 2,000 to 40,000 (in increments of 2,000) in ten scenarios. We compared two game scenarios (i.e., the opt-in game and the masking game) with two baseline scenarios (i.e., the no-protection scenario and the random masking scenario). For each game scenario, we considered four sub-scenarios, in each of which the data subject believes the number of records in the genetic genealogy dataset is fixed at 10,000, 20,000, or 30,000, as well as when they know the actual number. All other experimental settings are the same as those in the sensitivity analysis on parameters using simulated dataset (see note S9 for details).

In Fig. S3F, it can be seen that, although the masking game scenario is sub-optimal, in terms of the data subjects' average payoff when all data subjects are uncertain about the actual number of records in the genetic genealogy dataset, it still substantially outperforms the opt-in game scenarios and baseline scenarios in terms of both the payoff and the probability of an attack's success. This is particularly the case when the estimated value of the parameter is close to the actual one. In Figs. S3D, S3E, and S3F, it can be seen that, for each estimated value of the number of records in the genetic genealogy dataset, the greater the over(under)-estimation, the greater the under(over)-estimation of the probability of an attack's success, the greater the over(under)-estimation of privacy, and the greater the over(under)-estimation of the payoff. For each actual value of the number of records in the genetic genealogy dataset, the closer the estimated value is, the further the estimated probability of an attack's success will be to the optimal probability and the closer the estimated payoff will be to the optimal payoff. When the bias in the number of records in the genetic genealogy dataset is bounded within the range of 10% (i.e., 2,000 in 20,000, which is the default value), the estimation error of the expected probability of an attack's success will be bounded within the range of around 0.05 and the estimation error of the expected payoff will be bounded within a range of around \$7 (i.e., 8% of \$83 which is the optimal payoff). Regardless of how the actual number of records in the genetic genealogy dataset and the resulting probability of an attack's success change, the estimation error of the expected payoff will be bounded within a range of around \$35 (i.e., 42% of the optimal payoff), which is still smaller than \$63 (the difference between the results of the masking game and the opt-in game).

S10.3. Number of records in the identified dataset

Next, we investigated how bias in the data subject's knowledge about the number of records in the identified dataset affects the data subject's expected payoff and the probability of an attack's success. Figs. S3G, S3H, and S3I depicts the average probability of an attack's success, the average privacy, and the average payoff, respectively, in a pool of data subjects as the actual number of records in the identified dataset varies from 2,000 to 40,000 (in increments of 2,000) in ten scenarios. We compared two game scenarios with two baseline scenarios – the no-protection scenario and the random masking scenario. For each game scenario, we considered

four sub-scenarios in each of which the data subject believes the number of records in the identified dataset is fixed at 10,000, 20,000, or 30,000, as well as when they know the actual number. All of the other experimental settings are the same as those in the sensitivity analysis on parameters using simulated dataset (see note S9 for details).

In Figs. S3G, S3H, and S3I, it can be seen that the average probability of an attack's success does not increase, the average privacy does not decrease, and average payoff does not decrease as the actual number of records in the identified dataset increases in all scenarios. In Fig. S3I, it can be seen that although the masking game scenario is sub-optimal in terms of the data subjects' average payoff when all data subjects are uncertain about the actual number of records in the identified dataset, it still substantially outperforms the opt-in game scenarios and baseline scenarios in most cases in terms of both the payoff and the probability of an attack's success, especially when the estimated value of the parameter is close to the actual one. In Figs. S3G, S3H, and S3I, it can be seen that, for each estimated value of the number of records in the identified dataset, the greater its over-estimation, the greater the under-estimation of the probability of an attack's success, the greater the over-estimation of the privacy, and the greater the over-estimation of the payoff. For each actual value of the number of records in the identified dataset, the closer the estimated value is, the further the estimated probability of an attack's success will be to the optimal probability and the closer the estimated payoff will be to the optimal payoff. When the bias in the number of records in the identified dataset is bounded within the range of 10% (i.e., 2,000 in 20,000 which is the default value), the estimation error of the expected probability of an attack's success will be bounded within the range of around 0.0025 and the estimation error of the expected payoff will be bounded within the range of around $1 (e.g., 1% of $83 which is the optimal payoff). When the actual number of records in the identified dataset changes within the range of 90%, the estimation error of the expected payoff will be bounded within the range of around $28 (i.e., 34% of the optimal payoff), which is still much smaller than $63 (the difference between the results of the masking game and the opt-in game).

The results of the robustness analysis on these three parameters indicate that the superiority of the masking game in terms of the data subjects' average payoff is highly robust regardless of how a data subject is uncertain about these examined parameters and the corresponding probability of an attack's success. The parameters that can bring a higher level of uncertainty to the probability of an attack's success (e.g., the number of records in the genetic genealogy dataset) also lead to larger error to the estimated payoff of the data subject.

Supplementary Note S11. Relationships between evaluation metrics and the minority-support factor using simulated datasets.

Here, we analyze the relationships between eight evaluation metrics – namely, usefulness, privacy, utility, payoff, fairness with respect to usefulness, fairness with respect to privacy, fairness with respect to utility, and fairness with respect to payoff – and the minority-support factor (see Eq. S1 in note S2 for its definition) using simulated datasets. All experimental settings and examined scenarios are the same as those in the experiment described in note S8. Fig. S4 depicts how each evaluation metric changes as the minority-support factor varies from −1 to 4 (in increments of 0.5). There are several notable findings.

First, the masking game outperforms all other approaches in terms of the usefulness when the minority-support factor is in the [0, 4] range. Moreover, it outperforms other protection approaches in terms of fairness with respect to usefulness when the minority-support factor is in the [0.5, 1.5] range.

Second, from Fig. S4A, it can be seen that, in the masking game, the usefulness increases monotonically as the minority-support factor increases into the range of [0.5, 1.5] after which the usefulness decreases monotonically as the minority-support factor increases. This trend occurs for several reasons. Firstly, when the incentive for sharing a rare value and the incentive for sharing a frequent value are the same (i.e., the minority-support factor equals to zero), the rare values in an attribute are less likely to be shared in a protection scenario such as the masking game due to re-identification risks. As a consequence, the corresponding usefulness (i.e., the distance between distributions of the shared data and unprotected data, averaged across attributes) is not optimal. Secondly, as the minority-support factor increases from zero, the incentive for sharing a rare value increases and the incentive for sharing a frequent value decreases. As a result, a greater number of rare values (and thus fewer frequent values) will be shared in each attribute. This leads to the maximal usefulness for the shared dataset when the minority-support factor reaches a certain point (i.e., a value in the range of [0.5, 1.5]). Thirdly, after the minority-support factor reaches the certain point, if it further increases, more rare values (and fewer frequent values) will be shared in each attribute. Thus, the usefulness of the shared dataset will start to decrease monotonically. Fourthly, as the minority-support factor decreases from zero, the incentive for sharing a rare value in an attribute decreases and the incentive for sharing a frequent value in an attribute increases. As a result, more frequent values (and fewer rare values) will be shared in each attribute. This leads to a local minimal usefulness for the shared dataset when the minority-support factor reaches a certain point (i.e., a value smaller than −0.5) after which the sharing strategy, as well as the usefulness, will no longer change.

Third, it can be seen in Figs. S4A to S4D that, in the masking game, there is a tradeoff between the privacy, as well as the utility and the payoff, and the usefulness. Specifically, as the usefulness increases, the privacy tends to decrease. This trend occurs for several reasons. First, sharing more rare values in an attribute can exacerbate re-identification risk. As a result, as the minority-support factor decreases from a certain point (i.e., a value in the range of [0.5, 1.5]), fewer rare values will be shared, which leads to higher average privacy. Secondly, masking more frequent values in an attribute can mitigate re-identification risk. Thus, as the minority-support factor increases from the certain point, although more rare values will be shared, much

fewer frequent values will be shared as well. This leads to higher average privacy. Similarly, as the usefulness increases (decreases), the utility has a trend to decrease (increase). This trend also has several reasons behind it. Firstly, according to Eq. S1 (in note S2), the benefit function in the experiment is a truncated function of the sharing strategies. This implies that maximal benefit and maximal utility can be achieved even if only partial data are shared. Secondly, as the minority-support factor decreases from the certain point (i.e., a value in the range of [0.5, 1.5]), the incentive for sharing frequent values increases monotonically until it reaches the limit. As a result, each data subject will share attributes that have frequent values instead of sharing attributes that have rare values, which leads to higher average utility. Thirdly, as the minority-support factor increases from the certain point, the incentive for sharing rare values increases monotonically until it reaches the limit. Thus, each data subject will share attributes that have rare values instead of sharing attributes that have frequent values. This leads to higher average utility as well. It should be noted that, even if the sharing strategy is fixed, as in the random masking scenario and the *k*-anonymity scenario, this trend still holds true because of the design of the benefit function in Eq. S1. Fourthly, as the usefulness increases (decreases), the payoff has a trend to decreases (increase). This is because the payoff is calculated as a constant plus a weighted sum of the privacy and the utility, and thus trends in the same manner.

Fourth, from Fig. S4E, it can be seen that, in the masking game, the fairness with respect to usefulness increases monotonically as the minority-support factor increases until the minority-support factor reaches a value in the range of [0.5, 1.5] after which the fairness with respect to usefulness decreases monotonically as the minority-support factor increases. This trend occurs for several reasons. Firstly, when the incentive for sharing a rare value and the incentive for sharing a frequent value are the same (i.e., the minority-support factor equals to zero), rare values in an attribute are less likely to be shared in a protection scenario such as the masking game due to re-identification risks. As a result, the subset of the dataset corresponding to a minority group (e.g., a group of data subjects born in the 2000s) tends to have lower usefulness than the subset corresponding to a majority group (e.g., a group of data subjects born in the 1980s). This implies that the corresponding fairness with respect to usefulness is not optimal. Secondly, as the minority-support factor increases from zero, the incentive for sharing a rare value in an attribute increases and the incentive for sharing a frequent value in an attribute decreases. Thus, more rare values (and fewer frequent values) will be shared in each attribute in the masking game. Consequently, the subset of the dataset corresponding to a minority group tends to have similar level of usefulness with the subset corresponding to a majority group. This leads to the maximal fairness with respect to usefulness for the sharing process when the minority-support factor reaches a certain point (i.e., a value in the range of [0.5, 1.5]). Thirdly, after the minority-support factor reaches the certain point, if it further increases, more rare values (and fewer frequent values) will be shared in each attribute in the masking game. As a result, the subset of the dataset corresponding to a minority group tends to have higher usefulness than the subset corresponding to a majority group. And, consequentially, the usefulness of the shared dataset will start to decrease monotonically. Fourthly, as the minority-support factor decreases from zero, the incentive for sharing a rare value in an attribute decreases and the incentive for sharing a frequent value in an attribute increases. Thus, more frequent values (and fewer rare values) will be shared in each attribute in the masking game. Therefore, the subset of the dataset corresponding to a minority group tends to have much lower level of usefulness than the subset corresponding to a majority group. This leads to a local minimal fairness with respect to

usefulness for the shared dataset when the minority-support factor reaches a certain point (i.e., a value smaller than −0.5) after which the sharing strategy, as well as the fairness with respect to usefulness, will no longer change.

Fifth, from Figs. S4E to S4H, it can be seen that, in the masking game, there is a tradeoff between the fairness with respect to privacy, as well as the fairness with respect to utility and the fairness with respect to payoff, and the fairness with respect to usefulness. Specifically, as the fairness with respect to usefulness increases, the fairness with respect to privacy has a trend to decrease. This trend occurs for several reasons. Firstly, sharing more rare values in an attribute can exacerbate re-identification risk. As a result, as the minority-support factor decreases from the certain point, fewer rare values will be shared. Thus, a minority group tends to have higher average privacy as well as a majority group, which leads to higher fairness with respect to privacy. Secondly, masking more frequent values in an attribute can mitigate re-identification risk. So, as the minority-support factor increases from the certain point, more rare values and fewer frequent values will be shared. Thus, a minority group tends to have higher average privacy as well as a majority group. And this leads to higher fairness with respect to privacy as well. Similarly, as the fairness with respect to usefulness increases (decreases), the fairness with respect to utility has a trend to decrease (increase). This trend is realized for several reasons. Firstly, according to Eq. S1, the benefit function in the experiment is a truncated function of the sharing strategies. This implies that maximal benefit and maximal utility can be achieved even if only partial data are shared. Secondly, as the minority-support factor decreases from the certain point (i.e., a value in the range of [0.5, 1.5]), the incentive for sharing frequent values in an attribute increases monotonically until it reaches the limit. Thus, the average utility for each group increases – except for the majority groups that have already reached the limit in terms of the average utility. This leads to higher fairness with respect to utility. Thirdly, as the minority-support factor increases from the certain point, the incentive for sharing rare values in an attribute increases monotonically until it reaches the limit. As a consequence, the average utility for each group increases except those minority groups that have already reached the limit in terms of the average utility. This leads to higher fairness with respect to utility. Fourthly, as the fairness with respect to usefulness increases (decreases), the fairness with respect to payoff has a trend to decreases (increase). This is because the payoff is calculated as a constant plus a weighted sum of the privacy and the utility, and thus the corresponding fairness with respect to payoff shares the same trend with the fairness with respect to privacy and the fairness with respect to utility.

Last, based on the empirical evaluation for the masking game, 0.5 might be a better choice (compared to 0 and 1) for a data holder to set as the minority-support factor for several reasons. Firstly, in comparison to a minority-support factor of 1, a minority-support factor of 0.5 leads to notably higher fairness with respect to payoff, notably higher fairness with respect to utility, and almost the same values in terms of usefulness, fairness with respect to usefulness, and fairness with respect to privacy. Secondly, in comparison to a minority-support factor of 0, a minority-support factor of 0.5 leads to notably higher usefulness, substantially higher fairness with respect to usefulness, and almost the same values of fairness with respect to privacy, fairness with respect to utility, and fairness with respect to payoff. It should be noted that a data holder only cares about the usefulness and fairness measures.

Supplementary Note S12. Relationships between evaluation metrics and the confidence score.

Here, we analyze the relationships between three evaluation metrics (namely, utility, payoff, and inference correctness) and the confidence score in the case study based on Craig Venter's data and the Ysearch dataset. Fig. S5 shows two scatterplots of those measures associated with all searched strategies using the greedy algorithm with pruning in the case study. In each subplot, the horizontal axis indicates the adversary's confidence score (ranging from 0 to 1) for the inferred surname resulting from one of the data subject's strategies. The vertical axis in Fig. S5A indicates the data subject's data utility resulting from a strategy. At the same time, the vertical axis in Fig. S5B indicates the data subject's payoff resulting from a strategy. From Fig. S5A, we can find that the best strategy must be in the region (shaded in blue) enclosed by two sets of parallel lines, which can be represented by the following equations:

$$U \geq 1 - \left(\frac{1}{k} - \frac{1}{k'}\right)\frac{L}{B} = 1 - \left(\frac{1}{2} - \frac{1}{157{,}681}\right)\frac{\$150}{\$100} = 0.25, \quad \text{(S23)}$$

$$0 \leq U \leq 1, \quad \text{(S24)}$$

$$\hat{r} \leq \begin{cases} \max\left(\frac{kC}{L}, \theta\right) = \max\left(\frac{2 \times \$10}{\$150}, 0.5\right) = 0.5, & r = 1, \\ \max\left(\frac{k'C}{L}, \theta\right) = \max\left(\frac{157{,}681 \times \$10}{\$150}, 0.5\right) = 10{,}512.07, & r = 0, \end{cases} \quad \text{(S25)}$$

$$0 \leq \hat{r} \leq 1. \quad \text{(S26)}$$

Here, U is the utility of the shared data. k is the number of the query results of Craig Venter's demographic attributes with the surname. k' is the number of the query results of Craig Venter's demographic attributes without the surname. L is the loss from being re-identified. B is the maximal benefit of sharing all data. C is the cost of an attack. $\hat{r}$ is the confidence score as the estimated correctness of the inferred surname. r is the correctness of the inferred surname. θ is the threshold for confidence score.

The strategy in this region with the highest utility is the optimal strategy. The strategy on the bottom line of this region has the same payoff as the no-protection strategy. If no strategies are in this region, then the no-protection strategy is optimal. A more straightforward way to find the best strategy is through Fig. S5B, in which the best strategy is the strategy with the highest payoff.

From Fig. S5B, we can find that the success of the surname inference plays a considerable role in choosing the optimal strategy. However, this is not always the case. For a common surname, the surname inference tends to be always successful. However, the difference between a successful and unsuccessful attack regarding the data subject's loss is relatively small (i.e., close to zero) for a common surname. For a rare surname, the difference between a successful

and unsuccessful attack in terms of the loss to the data subject is relatively large (i.e., close to one). However, the surname inference will hardly be successful in this situation because it is highly likely that the surname does not exist in the referenced dataset. For the surname, Venter, which is a relatively rare surname (shared by 653 people in the United States according to the 2010 U.S. Census), the surname inference will be successful for some targets, and the difference between a successful attack and an unsuccessful attack in terms of the loss to the data subject substantially affects the payoff, which makes this two-stage re-identification attack quite successful. In the surname inference stage, only the success rate matters, whereas in the record linkage stage, only the number of matched identified records matters. Thus, in the two-stage attack, which integrates the surname inference and the record linkage, both success rate and group size matter, and the rareness of a surname will simultaneously affect these two variables.

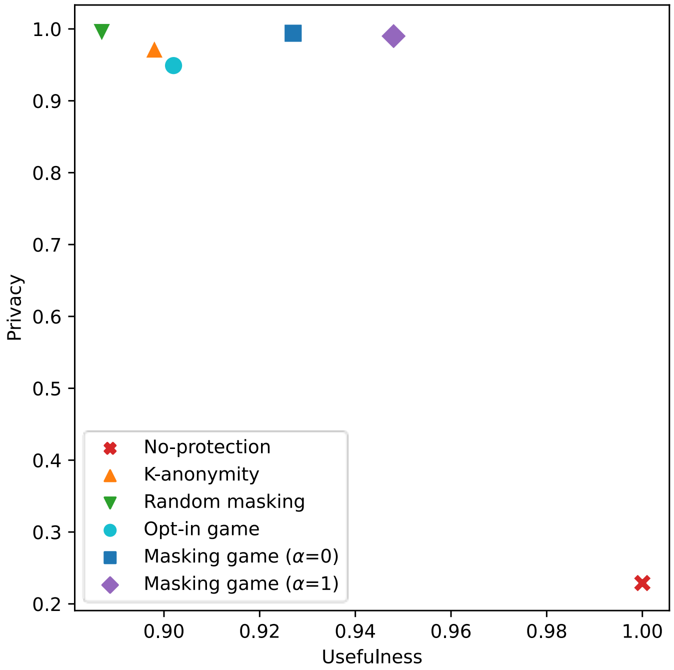

Fig. S1. Scatterplot of privacy and usefulness metrics in six scenarios against a multistage re-identification attack targeting a dataset of 1000 subjects.

Each mark corresponds to one scenario averaged across 100 runs. α represents the minority-support factor.

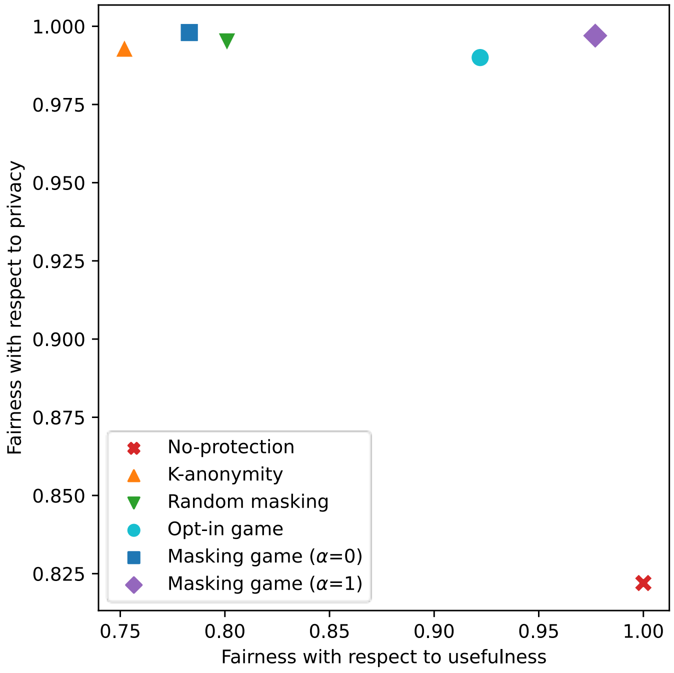

Fig. S2. Scatterplot of fairness with respect to privacy and fairness with respect to usefulness metrics in six scenarios against a multistage re-identification attack targeting a dataset of 1000 subjects.

Each mark corresponds to one scenario averaged across 100 runs. α represents the minority-support factor.

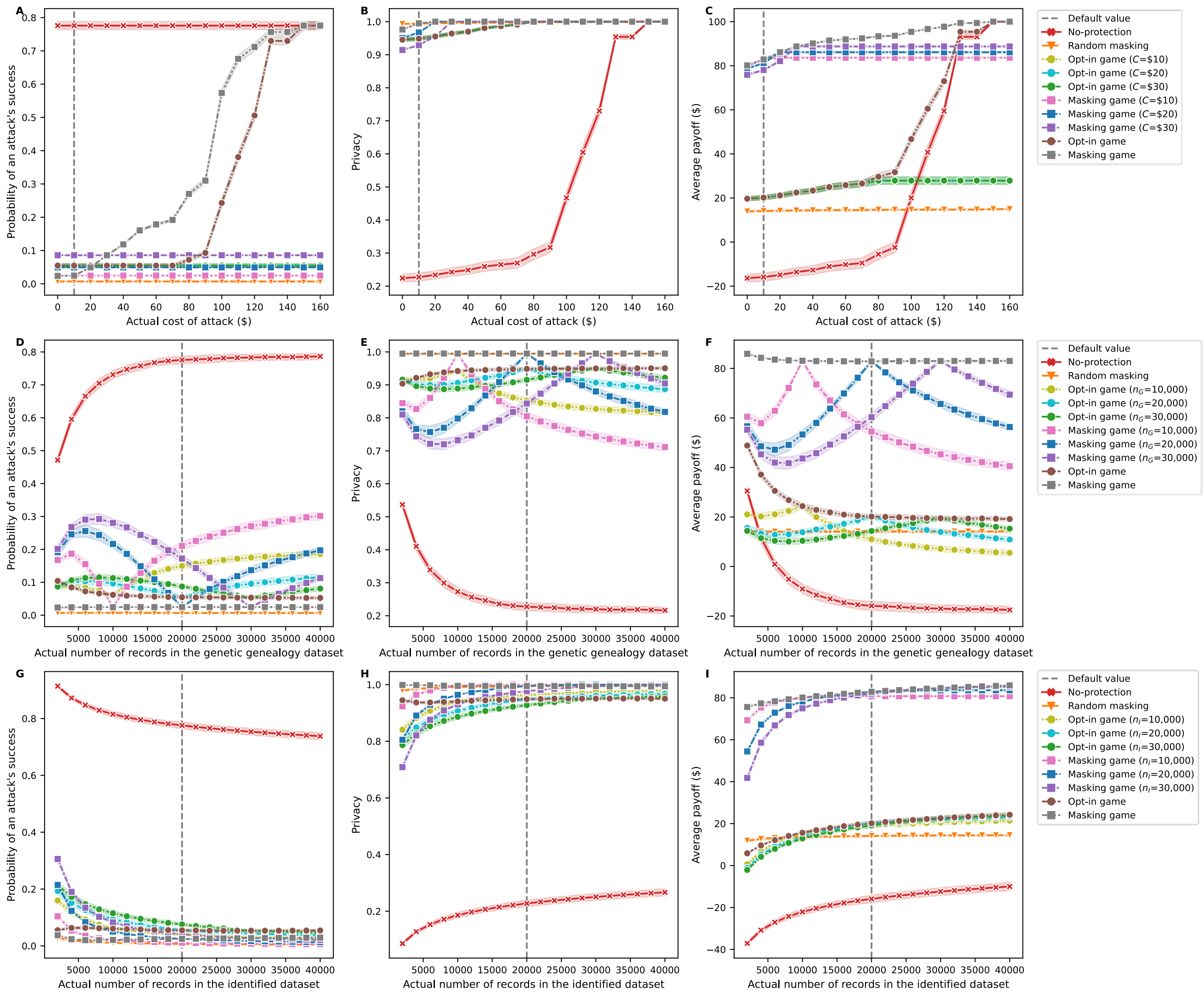

Fig. S3. Robustness of the data subjects' average payoff, privacy, and probability of an attack's success as a function of the actual values of three parameters in the form of line plots.

(**A**) Probability of success versus the cost of an attack. (**B**) Privacy versus the cost of an attack. (**C**) Payoff versus the cost of an attack. (**D**) Probability of success versus the number of records in the genetic genealogy dataset. (**E**) Privacy versus the number of records in the genetic genealogy dataset. (**F**) Payoff versus the number of records in the genetic genealogy dataset. (**G**) Probability of success versus the number of records in the identified dataset. (**H**) Privacy versus the number of records in the identified dataset. (**I**) Payoff versus the number of records in the identified dataset. Each line plot (depicted using Seaborn) shows the data subjects' average payoffs, privacy metrics, or probabilities of success, with error bars representing standard deviations, in ten scenarios. C represents the adversary's cost of an attack (estimated in the game model). n_I represents the number of records in the identified dataset (D_I) (estimated in the game

model). n_G represents the number of records in the genetic genealogy dataset (D_G) (estimated in the game model).

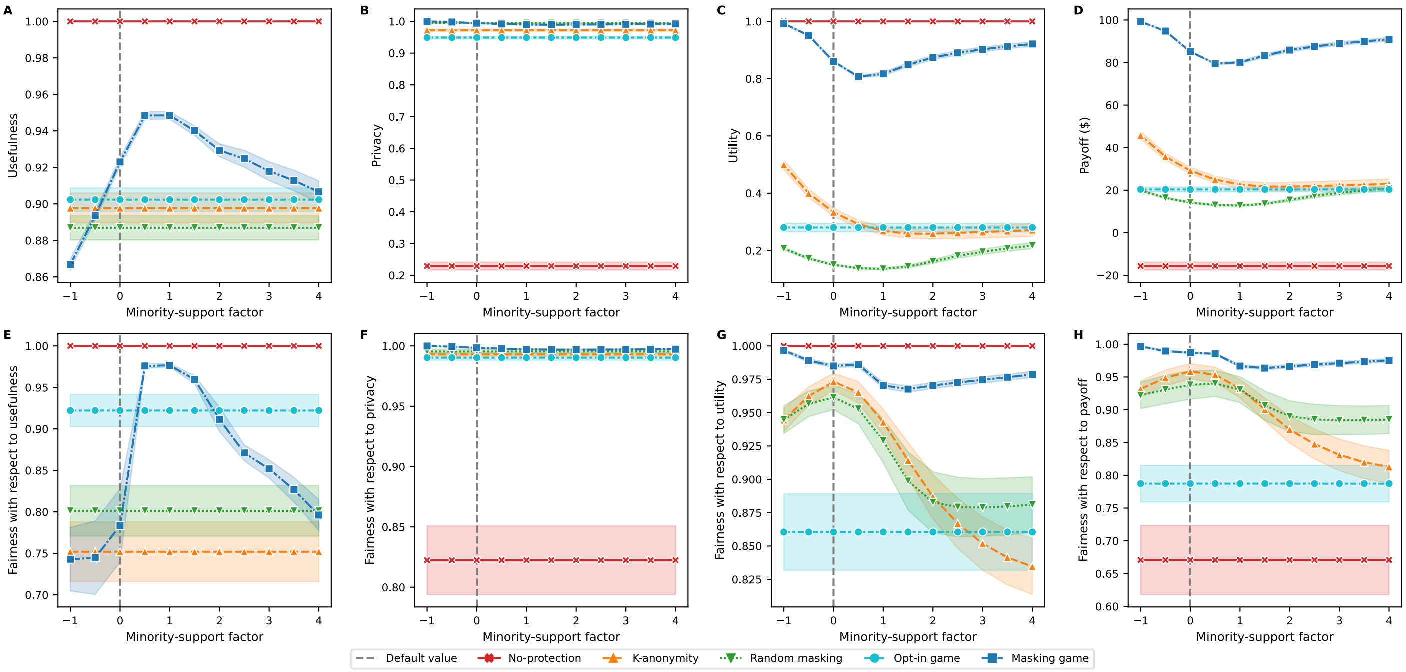

Fig. S4. Sensitivity of evaluation metrics as a function of the minority-support factor against a two-stage re-identification attack targeting 1000 data subjects in the form of line plots.

(**A**) Usefulness of the shared dataset. (**B**) Data subjects' average privacy. (**C**) Data subjects' average utility. (**D**) Data subjects' average payoff. (**E**) Fairness with respect to usefulness of the data sharing process. (**F**) Fairness with respect to privacy of the data sharing process. (**G**) Fairness with respect to utility of the data sharing process. (**H**) Fairness with respect to payoff of the data sharing process. Each line plot (depicted using Seaborn) shows a specific evaluation metric, with error bars representing standard deviations, in five scenarios.

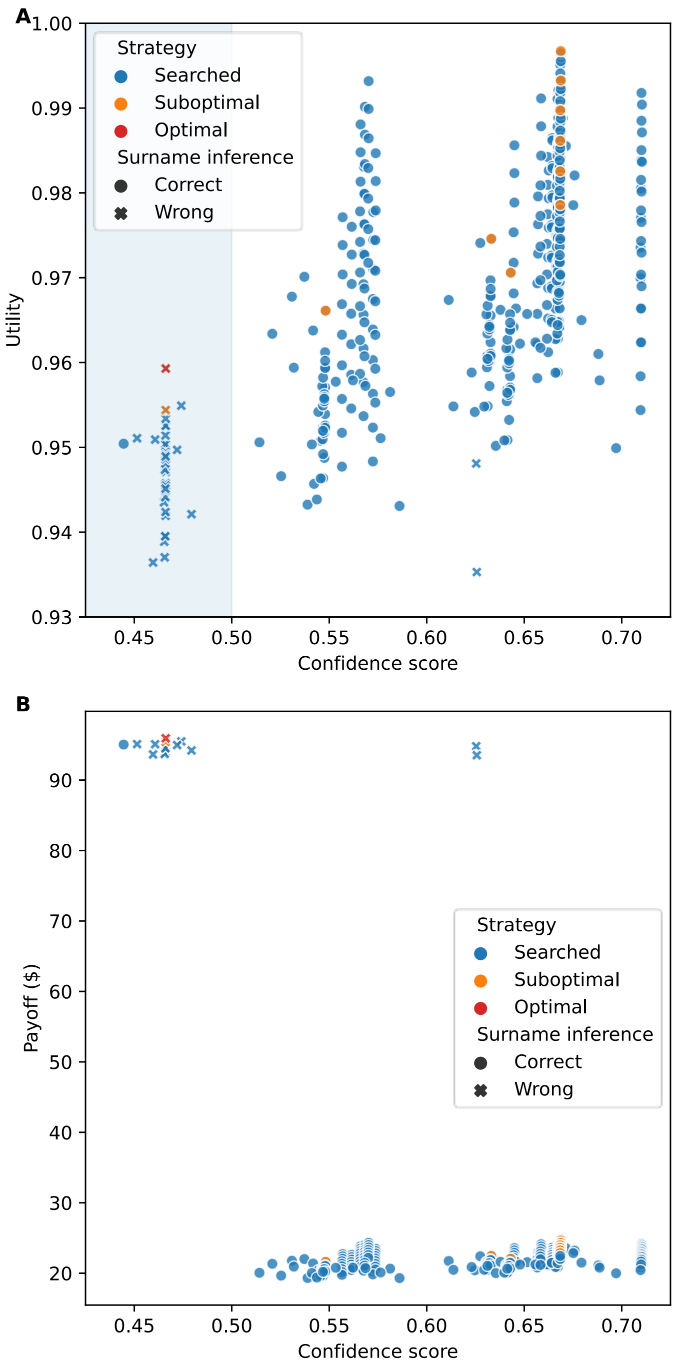

Fig. S5. Scatterplots of measures associated with all searched strategies in the masking game in the case study based on Craig Venter's data and the Ysearch dataset.

(**A**) Craig Venter's data utility and confidence score associated with each strategy. (**B**) Craig Venter's payoff and confidence score associated with each strategy. The strategy with the highest utility in the region shaded in blue is the optimal strategy. If there is no strategy in that region, the optimal strategy is the strategy that shares all data (i.e., the no-protection strategy).

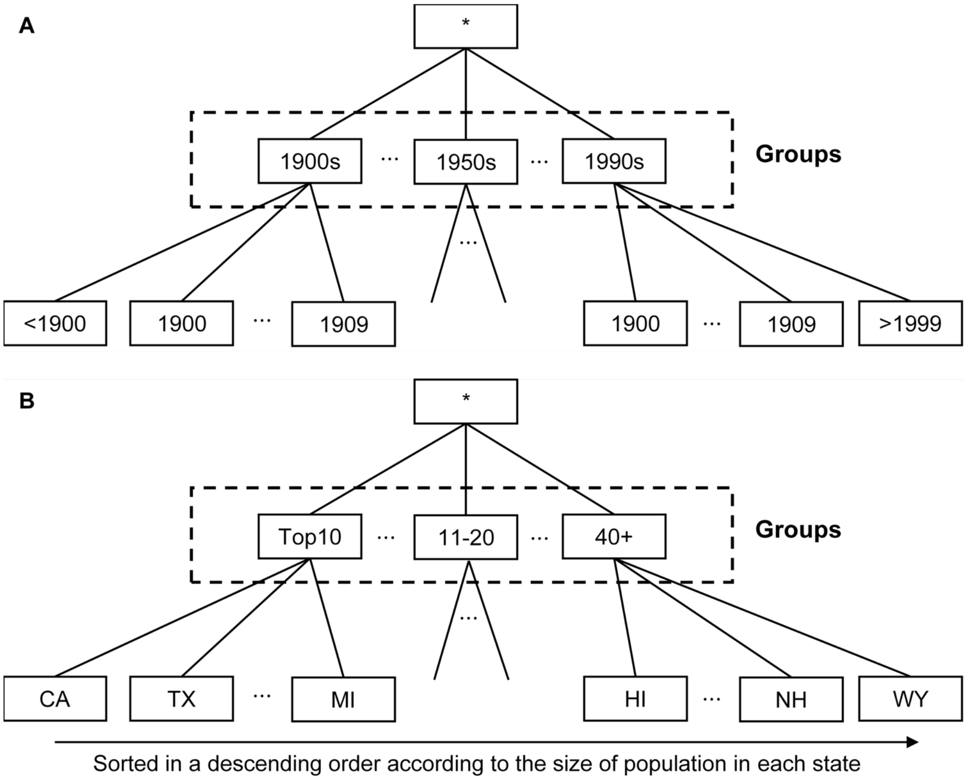

Fig. S6. Domain hierarchies for the demographic attributes used in the experiments. (**A**) Year of birth. (**B**) U.S. state of residence.

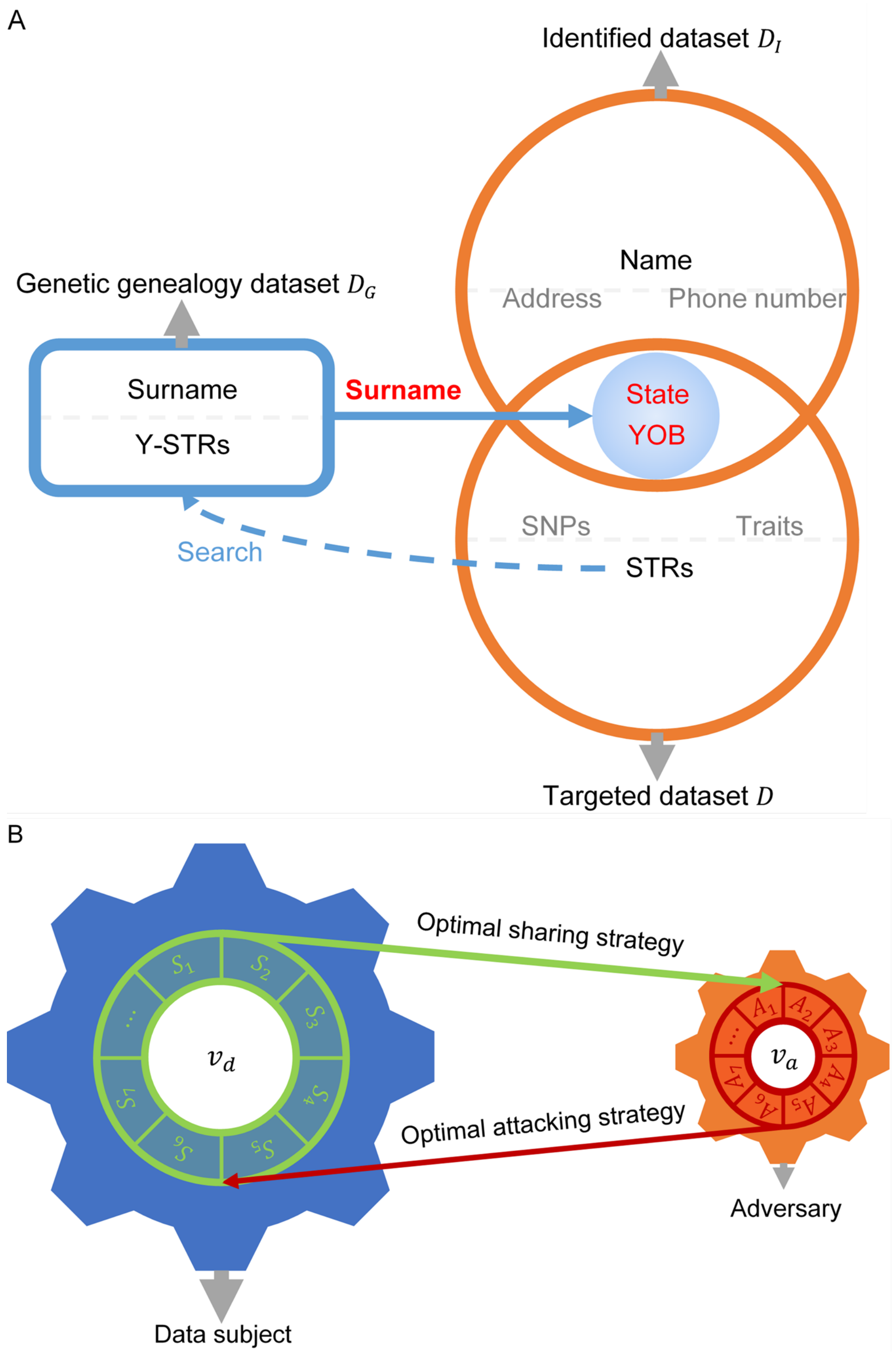

Fig. S7. An illustration of the Gymrek attack model and the two-player game model. (**A**) The Gymrek attack. In this attack model, attributes exist in both the targeted genomic dataset and the identified dataset include state of residence and year of birth. Genomic attributes such as SNP and Y-STR markers and phenotypic attributes such as eye colors and hair colors only exist in the targeted genomic dataset. Full name and contact information only exist in the identified dataset. Surnames could be inferred from the Y chromosome of a targeted DNA

sequence by searching a reference dataset, which enhances the record linkage attack. SNPs, single-nucleotide polymorphisms; STRs, short tandem repeats; Y-STRs, Y-chromosome short tandem repeats; YOB, year of birth. (**B**) The two-player game model. Given the adversary's attacking strategy, the data subject chooses the optimal sharing strategy that maximizes the data subject's payoff. Given the data subject's strategy, the adversary chooses the optimal attacking strategy that maximizes the adversary's payoff. The game is solved when the whole system is in a Nash equilibrium, which means that each player can find no better strategy if the other player does not change the chosen strategy. In the figure, v_d and v_a are payoffs (or utilities) for the data subject and the adversary, respectively. Notably, $\mathbf{S}_t$ represents the data subject's t^{th} sharing strategy, and the A_l represents the adversary's l^{th} attacking strategy.

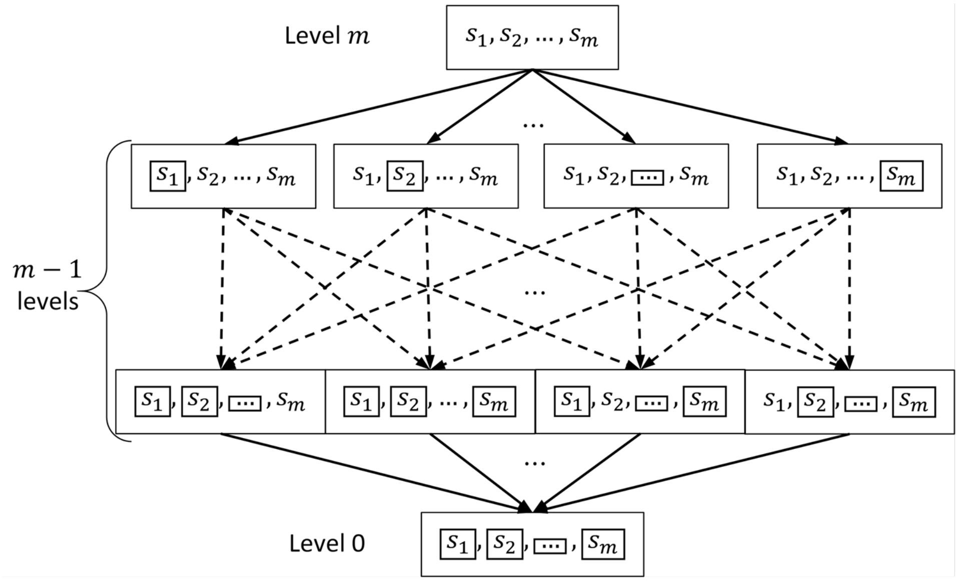

Fig. S8. The lattice representation of the data subject's strategy space for the masking game.

The node at the top represents the strategy of sharing all attributes, while the node at the bottom represents the strategy of sharing nothing. The level in which a node is located represents the number of shared attributes in the strategy that the node represents, and it equals the number of "children" the node has. Each arrow points from a "parent" to a "child". Notably, m is the number of attributes in the data subject's record, and s_j represents the data subject's sharing decision for the jth attribute.

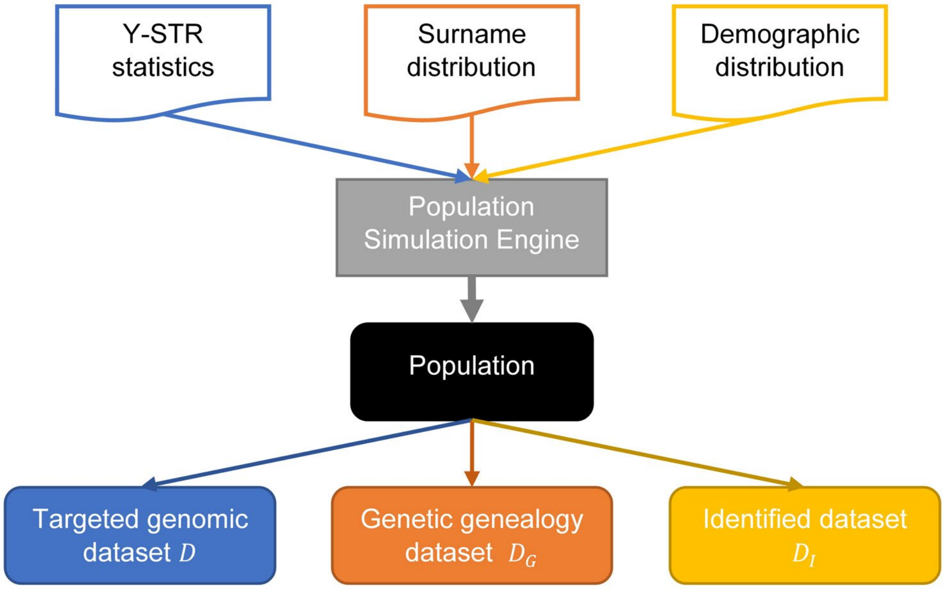

Fig. S9. The preparation pipeline for datasets used in the simulation-based experiments. With inputs of probability distributions and statistics of corresponding attributes, the population simulation engine generates simulated population, from which three datasets are derived. Y-STR, Y-chromosome short tandem repeat.

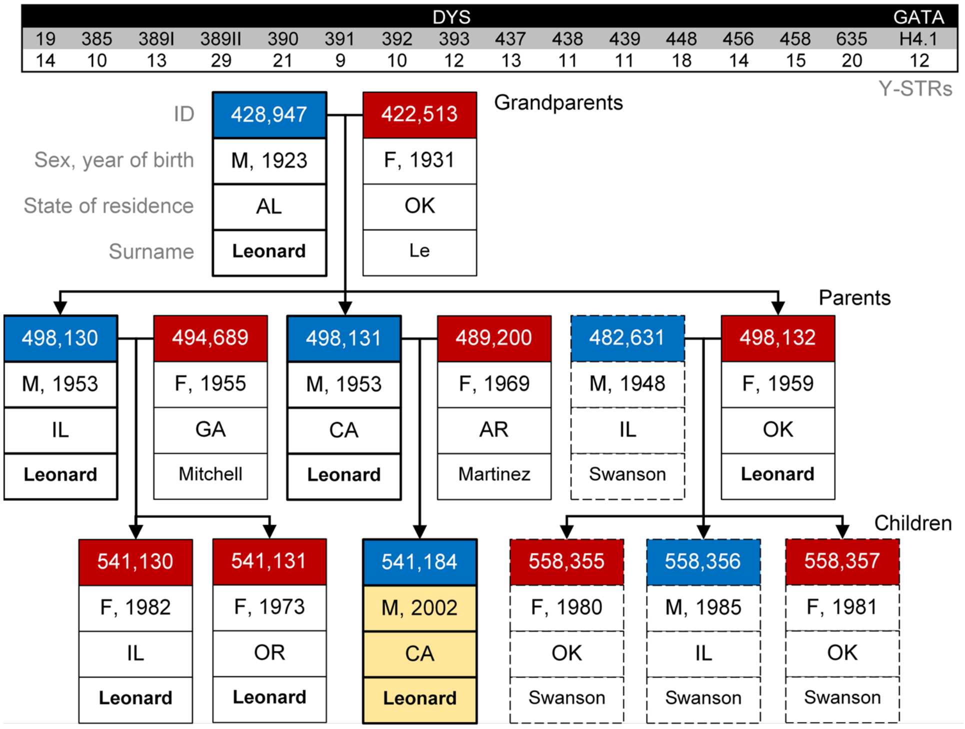

Fig. S10. The family tree of a simulated family across the last three generations with 20 primary attributes.

The 20 primary attributes include ID, sex, year of birth, state of residence, surname, and 16 Y-STRs. The Y-STRs of the data subject with ID 541,184 are shown at the top. Maiden names instead of surnames are shown for female members. For example, the maiden name of the person with ID 422,513 (i.e., grandmother of the data subject with ID 541,184) is Le instead of Leonard (which is not a typo). Y-STRs, Y-chromosome short tandem repeats; DYS, DNA Y-chromosome segment.

Table S1. Notation for parameters and variables, with corresponding descriptions, ranges, and settings, used throughout this work.

Notation	Description	Range	Setting
	Parameters in the experiment based on simulated datasets		
D	The targeted genomic database (or dataset)	/	/
D_G	The genetic genealogy database (or dataset) used for surname inference	/	/
D_I	The public identified database (or dataset) used for record linkage	/	/
m_d	The number of demographic attributes in dataset D	$\mathbb{N}$	2
m_g	The number of genomic attributes in dataset D	$\mathbb{N}$	12
m	The number of attributes in dataset D	$\mathbb{N}$	14
γ	The proportion of missing genomic data in dataset D_G	[0,1]	0.3
θ	The threshold for confidence score	[0,1]	0.5
n	The number of records in the targeted genomic dataset D	$\mathbb{N}$	1,000
n_G	The number of records in the genetic genealogy dataset D_G	$\mathbb{N}$	20,000
n_I	The number of records in the identified dataset D_I	$\mathbb{N}$	20,000
L	A data subject's loss from being re-identified	$\mathbb{R}^+$	\$150
B	The maximal benefit of sharing all data in a data record	$\mathbb{R}^+$	\$100
C	The adversary's cost of an attack (cost to execute an attack)	$\mathbb{R}^+$	\$10
N_i	The number of repeated runs for each experiment	$\mathbb{N}$	100
α	The minority-support factor	$\mathbb{R}$	0
	Parameters in the Gymrek attack		
N_e	The effective male population size	$\mathbb{N}$	10,000
T	The number of generations for the patrilineal surname system	$\mathbb{N}$	200
N_c	The number of pre-selected candidates	$\mathbb{N}$	10
P_t	Proportion of tolerable unmatched markers to maximal matched ones	[0,1]	0.2
θ_s	Jaro-Winkler string distance threshold	[0,1]	1
θ_m	Lower bound on the number of available markers	$\mathbb{N}$	17
	Parameters in the population simulation environment		
N_s	The number of surnames	$\mathbb{N}$	1,000
S_{mp}	The size of the simulated male population	$\mathbb{N}$	90,064
N_{sp}	The number of subpopulations	$\mathbb{N}$	3
P_m	The migration proportion	[0,1]	0.1
T_g	The number of generations	$\mathbb{N}$	10
S_{sp}	The size of each subpopulation in the first generation	$\mathbb{N}$	20,000
λ	The parameter for the zero-truncated Poisson distribution determining the number of children for each pair of parents	$\mathbb{R}^+$	1.6
	Variables ordered by their chronological introduction		
$\mathbf{s}$	A vector of binary elements representing an action of the data subject	$\mathbb{B}^m$	/
s_j	An element in $\mathbf{s}$ indicating whether j^{th} attribute is shared ($j \in [1, m]$)	$\mathbb{B}$	/
a	A binary variable representing an action of the adversary	$\mathbb{B}$	/
$b(\mathbf{s})$	The benefit of sharing data given the data subject's strategy	$\mathbb{R}^+$	/
$p(\mathbf{s})$	The probability of an attack's success given the subject's strategy	[0,1]	/
$v_d(\mathbf{s}, a)$	The data subject's payoff given both players' actions	$\mathbb{R}$	/
$v_a(\mathbf{s}, a)$	The adversary's payoff given both players' actions	$\mathbb{R}$	/
$\phi(\mathbf{s})$	The set of the adversary's best actions given the subject's strategy $\mathbf{s}$	$\{\mathbb{B}\}$	/
$\mathbf{s}^*$	The data subject's best strategy	$\mathbb{B}^m$	/

$\hat{p}(\mathbf{s})$	The adversary's estimated probability of an attack's success given **s**	[0,1]	/
$\hat{\phi}(\mathbf{s})$	The set of the adversary's estimated best responses to the subject's strategy **s**	$\{\mathbb{B}\}$	/
$p_1(\mathbf{s})$	The probability of stage I's success	[0,1]	/
$p_2(\mathbf{s})$	The probability of stage II's success given that stage I succeeds	[0,1]	/
$\hat{p}_1(\mathbf{s})$	The adversary's estimation on the probability of stage I's success	[0,1]	/
$p'(\mathbf{s})$	The probability of stage II's success given that stage I is omitted	[0,1]	/
$a'(\mathbf{s})$	A binary variable representing whether stage I is not omitted	$\mathbb{B}$	/
$\overline{V}$	The average payoff of n data subjects whose records are in dataset D	$\mathbb{R}$	/
V_i	The ith data subject's optimal payoff in dataset D	$\mathbb{R}$	/
$\overline{U}$	The average data utility of n subjects whose records are in dataset D	[0,1]	/
U_i	The ith subject's data utility in dataset D given the best sharing strategy	[0,1]	/
$\overline{P}$	The average privacy of n data subjects whose records are in dataset D	[0,1]	/
P_i	The ith subject's privacy in D given players' best actions and the record	[0,1]	/
$\mathbf{s}_i^*$	The ith data subject's best strategy in dataset D	$\mathbb{B}^m$	/
a_i^*	The adversary's best response for the ith data subject's best strategy	$\mathbb{B}$	/
σ_V	The standard deviation of n data subjects' payoffs	$\mathbb{R}$	/
σ_U	The standard deviation of n data subjects' data utility metrics	[0,1]	/
σ_P	The standard deviation of n data subjects' privacy metrics	[0,1]	/
U	The data subject's data utility given the best sharing strategy	[0,1]	/
w_j	The weight for the jth attribute of dataset D	$\mathbb{R}^+$	/
β_j	The minority level, indicates the proportion of the data subject's group in terms of the jth attribute in the population	$\mathbb{R}^+$	/
$n_{g,j}$	The number of groups in the jth attribute	$\mathbb{N}$	/
$r_{g,j}$	The proportion of the data subject's group in the underlying population in terms of the jth attribute	[0,1]	/
x	Demographic and genomic attributes in dataset D	/	/
x_G	The surname attribute in dataset D_G	/	/
x_I	Identity attributes (e.g., name) in dataset D_I	/	/
v_d	The subject's payoff if all data are shared and the adversary attacks	$\mathbb{R}$	/
p	The probability of an attack's success with all data being shared	[0,1]	/
s	The data subject's strategy in the opt-in game	$\mathbb{B}$	/
s^*	The data subject's best strategy in the opt-in game	$\mathbb{B}$	/
p_1	The probability of stage I's success in the opt-in game	[0,1]	/
p_2	The probability of stage II's success given stage I succeeds in the opt-in game	[0,1]	/
r	The correctness of the inferred surname	[0,1]	/
k	The number of matched identified records in the linkage upon quasi-identifiers and the inferred surname	$\mathbb{N}$	/
p'	The probability of stage II's success given stage I is omitted in the opt-in game	[0,1]	/
k'	Number of matched identified records in linkage upon quasi-identifiers	$\mathbb{N}$	/
v_a	The adversary's payoff if all data are shared and the adversary attacks	$\mathbb{R}$	/
$\hat{v}_a$	The adversary's estimated payoff with all data shared and the attack	$\mathbb{R}$	/
a^*	The adversary's best action in the opt-in game given the shared data	$\mathbb{B}$	/
$\hat{p}$	The adversary's estimated probability of an attack's success with all data being shared	[0,1]	/

$\hat{p}_1$	The adversary's estimation on the probability of stage I's success	[0,1]	/
$\hat{r}$	The adversary's estimated correctness of the inferred surname	[0,1]	/
a'	The adversary's optimal decision on whether to execute stage I	$\mathbb{B}$	/
$\hat{\phi}(s)$	The set of the adversary's estimated best responses to the data subject's strategy s	$\{\mathbb{B}\}$	/
$\mathbf{s}'$	A "child" of the strategy **s** in a lattice representation	$\mathbb{B}^m$	/
$\mathbf{s}''$	A "grandchild" of the strategy **s** in a lattice representation	$\mathbb{B}^m$	/
$\mathbf{H}$	A set of data points in a same attribute	$\{\mathbb{R}\}$	/
$\mathbf{H}'$	A set of data points (a subset of **H**) in a same attribute	$\{\mathbb{R}\}$	/
m_v	The number of distinct values in **H**	$\mathbb{N}$	/
h_i'	The relative frequency corresponding to i^{th} distinct value in $\mathbf{H}'$	[0,1]	/
h_i	The relative frequency corresponding to i^{th} distinct value in **H**	[0,1]	/
$d(\mathbf{H}',\mathbf{H})$	The variational distance between two sets of data points or distributions	[0,1]	/
D'	A released targeted dataset corresponding to D	/	/
$\mathbf{D}_j$	The set of data points in the jth attribute of dataset D	$\{\mathbb{R}\}$	/
$\mathbf{D}_j'$	The set of data points in the jth attribute of dataset D'	$\{\mathbb{R}\}$	/
$W(D',D)$	The usefulness measure given two datasets, D' and D	[0,1]	/
$D'_{ij,*}$	The subset of dataset D' consists of all data subjects in the ith group of the jth demographic attribute in dataset D'	/	/
$D_{ij,*}$	The subset of dataset D consists of all data subjects in the ith group of the jth demographic attribute in dataset D	/	/
$n_{d,j}$	The number of groups in the jth demographic attribute in dataset D	$\mathbb{N}$	/
$G(\cdot)$	The Gini coefficient of a set of numbers	[0,1]	/
m_d	The number of demographic attributes in dataset D	$\mathbb{N}$	/
$\mathbf{D}'_{ijk}$	The set of data points of the kth attribute in the ith group of the jth demographic attribute in dataset D'	$\{\mathbb{R}\}$	/
$\mathbf{D}_{ijk}$	The set of data points of the kth attribute in the ith group of the jth demographic attribute in dataset D	$\{\mathbb{R}\}$	/
$F_W(D',D)$	The fairness with respect to usefulness of a released dataset D', given dataset D	[0,1]	/
$n_{g,ij}$	The size of the ith group of the jth attribute in dataset D	$\mathbb{N}$	/
f_{kij}	An additive measure for the kth data subject in the ith group of the jth attribute in dataset D'	$\mathbb{R}$	/
$F_f(D',D)$	The fairness with respect to an additive measure (f) of a released dataset D', given D	[0,1]	/
$\rho(\mathbf{s})$	The sharing rate given a sharing strategy **s**	[0,1]	/
$\mathbf{S}_t$	The data subject's t^{th} sharing strategy ($t \in [1,2^m]$)	$\mathbb{B}^m$	/
$\mathbf{A}_l$	The adversary's l^{th} attacking strategy ($l \in [1,2^{2^m}]$)	$\mathbb{B}^{2^m}$	/

Table S2. Statistics for 16 Y-STR genomic attributes used in the population simulation. Y-STR, Y-chromosome short tandem repeat; DYS, DNA Y-chromosome segment.

Y-STR	Range of repeats	Mutation rate (*10^{-3}) (2018)	Mutation number (2018)	Mutation rate (*10^{-3}) (2008)	Mutation number (2008)
DYS19	[6, 20]	2.202	37 (in 16,801)	2.381	23 (in 9,658)
DYS385	[6, 28]	2.508	70 (in 27,911)	2.081	31 (in 14,896)
DYS389I	[9, 17]	2.724	41 (in 15050)	1.781	14 (in 7,862)
DYS389II	[23, 36]	4.327	65 (in 15,021)	2.803	22 (in 7,849)
DYS390	[16, 30]	2.083	34 (in 16323)	2.298	21 (in 9,140)
DYS391	[5, 16]	2.531	41 (in 16197)	3.081	28 (in 9,089)
DYS392	[5, 20]	0.496	8 (in 16129)	0.552	5 (in 9,053)
DYS393	[7, 18]	1.068	16 (in 14975)	0.893	7 (in 7,842)
DYS437	[9, 22]	1.320	15 (in 11363)	1.498	7 (in 4,672)
DYS438	[5, 19]	0.351	4 (in 11384)	0.425	2 (in 4,709)
DYS439	[5, 19]	5.459	62 (in 11358)	5.762	27 (in 4,686)
DYS448	[13, 25]	1.385	11 (in 7940)	1.590	2 (in 1,258)
DYS456	[9, 24]	4.408	35 (in 7940)	4.769	6 (in 1,258)
DYS458	[10, 24]	6.172	49 (in 7939)	6.359	8 (in 1,258)
DYS635	[12, 30]	4.211	37 (in 8787)	3.754	8 (in 2,131)
GATA H4.1	[6, 20]	3.010	27 (in 8971)	2.180	5 (in 2,294)

Table S3. Usefulness of data shared in five scenarios. DYS, DNA Y-chromosome segment.

**Attribute**	**Scenario**					**Weight**
	**Random masking**	***k*-anonymity**	**Opt-in game**	**Masking game ($\alpha = 0$)**	**Masking game ($\alpha = 1$)**	
Year of birth	0.715	0.898	0.804	0.991	0.900	4.487
State	0.810	0.813	0.863	0.576	0.822	3.475
DYS19	0.943	0.913	0.931	0.984	0.986	1.624
DYS385	0.908	0.930	0.927	0.989	0.984	2.185
DYS389I	0.954	0.906	0.945	0.972	0.985	1.376
DYS389II	0.937	0.913	0.939	0.988	0.986	1.692
DYS390	0.939	0.908	0.937	0.986	0.984	1.665
DYS391	0.957	0.901	0.943	0.957	0.985	1.231
DYS392	0.943	0.912	0.930	0.981	0.980	1.615
DYS393	0.951	0.912	0.928	0.967	0.982	1.405
DYS437	0.958	0.918	0.936	0.967	0.985	1.303
DYS438	0.952	0.912	0.920	0.974	0.990	1.531
DYS439	0.944	0.914	0.930	0.970	0.976	1.573
DYS448	0.942	0.910	0.937	0.977	0.979	1.608
**Usefulness**	**0.887**	**0.898**	**0.902**	**0.927**	**0.948**	

Table S4. Relative difference between the shared and the unprotected datasets in terms of the sharing rate in five scenarios. DYS, DNA Y-chromosome segment.

**Attribute**	**Scenario**					**Weight**
	**Random masking**	***k*-anonymity**	**Opt-in game**	**Masking game ($\alpha = 0$)**	**Masking game ($\alpha = 1$)**	
Year of birth	0.848	0.314	0.720	0.011	0.321	4.487
State	0.850	0.509	0.720	0.592	0.352	3.475
DYS19	0.849	0.842	0.720	0.052	0.076	1.624
DYS385	0.850	0.414	0.720	0.039	0.050	2.185
DYS389I	0.848	0.901	0.720	0.118	0.111	1.376
DYS389II	0.853	0.788	0.720	0.038	0.083	1.692
DYS390	0.854	0.812	0.720	0.045	0.084	1.665
DYS391	0.852	0.916	0.720	0.163	0.147	1.231
DYS392	0.849	0.721	0.720	0.056	0.092	1.615
DYS393	0.849	0.875	0.720	0.105	0.095	1.405
DYS437	0.849	0.890	0.720	0.139	0.128	1.303
DYS438	0.852	0.861	0.720	0.094	0.057	1.531
DYS439	0.849	0.763	0.720	0.102	0.150	1.573
DYS448	0.851	0.787	0.720	0.099	0.106	1.608
**Weighted average**	**0.850**	**0.666**	**0.720**	**0.137**	**0.166**	

Table S5. Relative difference between the shared and the unprotected datasets in terms of the number of distinct values in five scenarios. DYS, DNA Y-chromosome segment.

Attribute	Scenario					Original # of distinct values	Weight
	Random masking	*k*-anonymity	Opt-in game	Masking game ($\alpha = 0$)	Masking game ($\alpha = 1$)		
Year of birth	0.279	0.150	0.165	0.004	0.016	99.46	4.487
State	0.223	0.288	0.185	0.495	0.001	50.01	3.475
DYS19	0.145	0.181	0.143	0.011	0.000	6.23	1.624
DYS385	0.047	0.039	0.016	0.001	0.000	12.34	2.185
DYS389I	0.129	0.296	0.106	0.033	0.000	4.79	1.376
DYS389II	0.106	0.151	0.087	0.010	0.000	6.89	1.692
DYS390	0.048	0.122	0.044	0.009	0.000	6.44	1.665
DYS391	0.071	0.329	0.069	0.030	0.000	4.41	1.231
DYS392	0.037	0.156	0.032	0.001	0.000	7.10	1.615
DYS393	0.069	0.207	0.034	0.002	0.000	5.01	1.405
DYS437	0.085	0.236	0.067	0.022	0.000	4.39	1.303
DYS438	0.006	0.072	0.008	0.000	0.000	5.01	1.531
DYS439	0.177	0.266	0.186	0.069	0.000	6.72	1.573
DYS448	0.064	0.162	0.044	0.006	0.000	6.41	1.608
**Weighted average**	**0.133**	**0.185**	**0.100**	**0.076**	**0.003**		

Table S6. Relative difference between the shared and the unprotected datasets in terms of the entropy in five scenarios. DYS, DNA Y-chromosome segment.

**Attribute**	**Scenario**					**Original entropy**	**Weight**
	**Random masking**	***k*-anonymity**	**Opt-in game**	**Masking game ($\alpha = 0$)**	**Masking game ($\alpha = 1$)**		
Year of birth	0.070	0.030	0.040	0.001	0.004	4.436	4.487
State	0.042	0.140	0.059	0.352	0.021	3.457	3.475
DYS19	0.025	0.072	0.066	0.015	0.012	1.435	1.624
DYS385	0.022	0.054	0.027	0.004	0.007	2.235	2.185
DYS389I	0.037	0.099	0.061	0.036	0.015	1.086	1.376
DYS389II	0.028	0.079	0.054	0.012	0.011	1.542	1.692
DYS390	0.031	0.098	0.059	0.015	0.015	1.499	1.665
DYS391	0.054	0.216	0.129	0.103	0.034	0.903	1.231
DYS392	0.035	0.131	0.086	0.027	0.022	1.432	1.615
DYS393	0.043	0.110	0.100	0.052	0.022	1.127	1.405
DYS437	0.041	0.126	0.098	0.066	0.025	0.977	1.303
DYS438	0.025	0.086	0.080	0.033	0.011	1.297	1.531
DYS439	0.029	0.098	0.077	0.038	0.023	1.370	1.573
DYS448	0.031	0.098	0.067	0.030	0.019	1.423	1.608
**Weighted average**	**0.040**	**0.095**	**0.065**	**0.069**	**0.015**		

Table S7. Relative difference between the shared and the unprotected datasets in terms of the Spearman correlation coefficient in five scenarios. DYS, DNA Y-chromosome segment.

**Attribute**	**Scenario**					**Weight**
	**Random masking**	***k*-anonymity**	**Opt-in game**	**Masking game ($\alpha = 0$)**	**Masking game ($\alpha = 1$)**	
Year of birth	2.128	3.193	0.517	0.174	0.262	4.487
State	2.088	2.233	0.517	0.512	0.219	3.475
DYS19	2.086	1.674	0.544	0.194	0.151	1.624
DYS385	2.098	1.168	0.535	0.176	0.140	2.185
DYS389I	2.072	2.100	0.526	0.233	0.167	1.376
DYS389II	2.228	1.562	0.542	0.191	0.153	1.692
DYS390	2.131	1.700	0.552	0.195	0.149	1.665
DYS391	2.163	2.248	0.548	0.258	0.181	1.231
DYS392	2.145	1.342	0.550	0.198	0.164	1.615
DYS393	2.112	1.788	0.546	0.235	0.157	1.405
DYS437	2.104	1.900	0.526	0.240	0.172	1.303
DYS438	2.121	1.675	0.560	0.212	0.145	1.531
DYS439	2.091	1.435	0.534	0.228	0.187	1.573
DYS448	2.121	1.565	0.549	0.203	0.163	1.608
**Weighted average**	**2.119**	**1.980**	**0.535**	**0.244**	**0.184**	

Table S8. Absolute difference between numerical attributes in the shared and the unprotected datasets in terms of arithmetic mean in five scenarios. DYS, DNA Y-chromosome segment.

**Attribute**	**Scenario**					**Original mean**
	**Random masking**	***k*-anonymity**	**Opt-in game**	**Masking game ($\alpha = 0$)**	**Masking game ($\alpha = 1$)**	
Year of birth	1.531	0.509	1.514	0.074	0.440	1948.92
DYS19	0.067	0.091	0.062	0.014	0.010	13.72
DYS385	0.143	0.253	0.154	0.028	0.047	13.16
DYS389I	0.041	0.085	0.037	0.019	0.007	11.96
DYS389II	0.071	0.077	0.054	0.011	0.011	28.38
DYS390	0.067	0.075	0.055	0.011	0.011	22.59
DYS391	0.038	0.079	0.032	0.021	0.006	9.31
DYS392	0.078	0.140	0.122	0.030	0.037	11.31
DYS393	0.051	0.073	0.038	0.025	0.006	11.95
DYS437	0.042	0.085	0.076	0.043	0.017	13.51
DYS438	0.057	0.092	0.087	0.019	0.008	9.61
DYS439	0.056	0.058	0.044	0.019	0.011	10.58
DYS448	0.067	0.062	0.039	0.013	0.008	18.42
**Average**	**0.178**	**0.129**	**0.178**	**0.025**	**0.048**	

Table S9. Absolute difference between numerical attributes in the shared and the unprotected datasets in terms of standard deviation in five scenarios standard. DYS, DNA Y-chromosome segment.

**Attribute**	**Scenario**					**Original standard deviation**
	**Random masking**	***k*-anonymity**	**Opt-in game**	**Masking game $(\alpha = 0)$**	**Masking game $(\alpha = 1)$**	
Year of birth	0.773	1.080	0.772	0.048	0.857	23.78
DYS19	0.038	0.089	0.091	0.024	0.018	1.04
DYS385	0.098	0.227	0.095	0.018	0.028	2.57
DYS389I	0.031	0.073	0.049	0.030	0.014	0.73
DYS389II	0.046	0.115	0.087	0.022	0.020	1.14
DYS390	0.050	0.138	0.085	0.024	0.025	1.10
DYS391	0.029	0.103	0.064	0.053	0.019	0.60
DYS392	0.047	0.079	0.057	0.021	0.012	1.33
DYS393	0.037	0.081	0.076	0.045	0.020	0.76
DYS437	0.025	0.069	0.053	0.040	0.017	0.68
DYS438	0.035	0.075	0.066	0.032	0.012	0.94
DYS439	0.036	0.112	0.094	0.050	0.033	0.96
DYS448	0.042	0.116	0.086	0.042	0.029	1.01
**Average**	**0.099**	**0.181**	**0.129**	**0.035**	**0.085**	

Table S10. Absolute difference between numerical attributes in the shared and the unprotected datasets in terms of skewness in five scenarios. DYS, DNA Y-chromosome segment.

**Attribute**	**Scenario**					**Original skewness**
	**Random masking**	***k*-anonymity**	**Opt-in game**	**Masking game ($\alpha = 0$)**	**Masking game ($\alpha = 1$)**	
Year of birth	0.096	0.034	0.075	0.004	0.028	0.03
DYS19	0.103	0.165	0.110	0.016	0.019	0.34
DYS385	0.115	0.065	0.104	0.014	0.026	0.57
DYS389I	0.097	0.234	0.089	0.041	0.013	0.25
DYS389II	0.113	0.151	0.097	0.014	0.014	0.19
DYS390	0.107	0.178	0.089	0.018	0.017	−0.25
DYS391	0.159	0.482	0.233	0.140	0.039	0.44
DYS392	0.116	0.124	0.101	0.020	0.028	0.37
DYS393	0.120	0.211	0.114	0.043	0.010	0.39
DYS437	0.147	0.276	0.243	0.126	0.047	0.69
DYS438	0.101	0.192	0.245	0.062	0.023	0.46
DYS439	0.127	0.188	0.113	0.042	0.018	0.18
DYS448	0.121	0.171	0.120	0.031	0.014	0.07
**Average**	**0.117**	**0.190**	**0.133**	**0.044**	**0.023**	

Table S11. Fairness with respect to usefulness, privacy, payoff, utility, and sharing rate in six scenarios. Scenarios include (1) no protection, (2) random masking, (3) k-anonymity, (4) opt-in game, (5) masking game with a minority-support factor of zero, and (6) masking game with a minority-support factor of one.

**Metric**	**Scenario**	**Year of Birth Group**										**State of Residence Group**					**Fairness**
		**1**	**2**	**3**	**4**	**5**	**6**	**7**	**8**	**9**	**10**	**1**	**2**	**3**	**4**	**5**	
**Usefulness**	**2**	0.204	0.474	0.517	0.445	0.521	0.494	0.469	0.517	0.401	0.183	0.689	0.549	0.478	0.332	0.189	**0.801**
	**3**	0.213	0.456	0.481	0.446	0.522	0.482	0.459	0.517	0.395	0.164	0.798	0.595	0.458	0.306	0.109	**0.752**
	**4**	0.636	0.843	0.857	0.831	0.855	0.843	0.827	0.851	0.797	0.583	0.899	0.840	0.805	0.720	0.513	**0.922**
	**5**	0.817	0.906	0.913	0.896	0.914	0.904	0.903	0.916	0.889	0.789	0.923	0.683	0.446	0.174	0.052	**0.783**
	**6**	0.826	0.887	0.891	0.886	0.892	0.886	0.886	0.892	0.878	0.809	0.922	0.844	0.830	0.807	0.791	**0.977**
**Privacy**	**1**	0.159	0.246	0.258	0.237	0.241	0.230	0.215	0.231	0.197	0.124	0.264	0.213	0.188	0.134	0.075	**0.822**
	**2**	0.984	0.995	0.996	0.996	0.995	0.995	0.994	0.996	0.994	0.982	0.997	0.994	0.991	0.986	0.983	**0.995**
	**3**	0.970	0.972	0.975	0.972	0.971	0.971	0.972	0.973	0.972	0.971	0.977	0.962	0.966	0.969	0.981	**0.993**
	**4**	0.939	0.946	0.950	0.945	0.951	0.949	0.951	0.953	0.949	0.942	0.961	0.939	0.927	0.924	0.943	**0.990**
	**5**	0.998	0.994	0.994	0.995	0.993	0.995	0.994	0.993	0.995	0.999	0.991	0.998	1.000	1.000	1.000	**0.998**
	**6**	0.992	0.989	0.989	0.989	0.989	0.990	0.990	0.990	0.991	0.993	0.990	0.983	0.991	0.999	1.000	**0.997**
**Payoff**	**1**	−26.2	−13.1	−11.3	−14.4	−13.9	−15.5	−17.7	−15.3	−20.4	−31.4	−10.4	−18.0	−21.8	−30.0	−38.7	**0.671**
	**2**	12.72	14.28	14.35	14.28	14.28	14.33	14.04	14.49	14.06	12.52	14.59	14.15	13.70	12.92	12.27	**0.938**
	**3**	28.61	28.88	29.50	29.00	29.21	29.18	29.18	29.36	29.22	29.51	30.22	27.47	27.84	28.47	29.30	**0.959**
	**4**	12.86	21.89	23.33	20.99	21.60	20.46	19.06	20.79	17.17	9.51	24.46	18.27	15.19	9.54	4.71	**0.787**
	**5**	81.76	85.30	85.98	84.73	85.78	85.31	84.78	85.99	83.83	81.19	88.02	81.52	81.34	81.20	81.22	**0.987**
	**6**	88.60	80.09	79.78	79.94	79.25	79.39	79.21	79.07	80.65	91.20	80.59	76.48	78.41	84.85	94.77	**0.967**
**Utility**	**2**	0.152	0.150	0.150	0.149	0.150	0.150	0.149	0.150	0.149	0.152	0.150	0.150	0.151	0.150	0.148	**0.961**
	**3**	0.332	0.331	0.333	0.332	0.335	0.335	0.334	0.334	0.334	0.339	0.336	0.332	0.329	0.332	0.321	**0.973**
	**4**	0.220	0.300	0.308	0.293	0.289	0.282	0.265	0.278	0.248	0.183	0.303	0.274	0.261	0.210	0.132	**0.860**
	**5**	0.820	0.862	0.868	0.854	0.868	0.861	0.857	0.870	0.846	0.813	0.894	0.819	0.814	0.812	0.812	**0.985**
	**6**	0.897	0.817	0.814	0.815	0.809	0.809	0.808	0.806	0.820	0.923	0.820	0.790	0.797	0.850	0.948	**0.970**
**Sharing rate**	**2**	0.151	0.150	0.150	0.150	0.149	0.149	0.149	0.150	0.150	0.153	0.150	0.150	0.151	0.150	0.147	**0.964**
	**3**	0.294	0.253	0.252	0.260	0.252	0.257	0.259	0.250	0.268	0.314	0.245	0.270	0.273	0.285	0.278	**0.944**
	**4**	0.220	0.300	0.308	0.293	0.289	0.282	0.265	0.278	0.248	0.183	0.303	0.274	0.261	0.210	0.132	**0.860**
	**5**	0.866	0.885	0.887	0.879	0.885	0.882	0.880	0.886	0.873	0.863	0.901	0.859	0.856	0.855	0.856	**0.992**
	**6**	0.805	0.874	0.878	0.868	0.876	0.870	0.869	0.876	0.858	0.775	0.886	0.855	0.844	0.830	0.795	**0.980**

Data S1. (separate file)

Computer code and relevant datasets.

REFERENCES AND NOTES

1. All of Us research program investigators, The "All of Us" research program. *N. Engl. J. Med.* **381**, 668–676 (2019).

2. J. W. Hazel, C. Slobogin, Who knows what, and when?: A survey of the privacy policies proffered by U.S. direct-to-consumer genetic testing companies. *Cornell J. Law Public Policy* **28**, 35–66 (2018).

3. W. N. Price II, I. G. Cohen, Privacy in the age of medical big data. *Nat. Med.* **25**, 37–43 (2019).

4. US Department of Health and Human Services Office for Civil Rights, Standards for privacy and individually identifiable health information; final rule. *Fed. Regist.* **67**, 53181–53273 (2002).

5. D. G. Wheeland, Final NIH genomic data sharing policy. *Fed. Regist.* **79**, 51345–51354 (2014).

6. M. Shabani, P. Borry, Rules for processing genetic data for research purposes in view of the new EU General Data Protection Regulation. *Eur. J. Hum. Genet.* **26**, 149–156 (2018).

7. Y. Erlich, A. Narayanan, Routes for breaching and protecting genetic privacy. *Nat. Rev. Genet.* **15**, 409–421 (2014).

8. M. Naveed, E. Ayday, E. W. Clayton, J. Fellay, C. A. Gunter, J.-P. Hubaux, B. A. Malin, X. Wang, Privacy in the genomic era. *ACM Comput. Surv.* **48**, 6 (2015).

9. N. von Thenen, E. Ayday, A. E. Cicek, Re-identification of individuals in genomic data-sharing beacons via allele inference. *Bioinformatics* **35**, 365–371 (2019).

10. M. Shabani, L. Marelli, Re-identifiability of genomic data and the GDPR: Assessing the re-identifiability of genomic data in light of the EU General Data Protection Regulation. *EMBO Rep.* **20**, e48316 (2019).

11. B. Greshake, P. E. Bayer, H. Rausch, J. Reda, OpenSNP–a crowdsourced web resource for personal genomics. *PLOS ONE* **9**, e89204 (2014).

12. J. L. Roberts, S. Pereira, A. L. McGuire, Should you profit from your genome? *Nat. Biotechnol.* **35**, 18–20 (2017).

13. R. Kain, S. Kahn, D. Thompson, D. Lewis, D. Barker, C. Bustamante, C. Cabou, A. Casdin, F. Garcia, J. Paragas, A. Patrinos, A. Rajagopal, S. F. Terry, A. Van Zeeland, E. Yu, Y. Erlich, D. Barry, Database shares that transform research subjects into partners. *Nat. Biotechnol.* **37**, 1112–1115 (2019).

14. F. K. Dankar, Data privacy through participant empowerment. *Nat. Comput. Sci.* **1**, 175–176 (2021).

15. B. Malin, L. Sweeney, Determining the identifiability of DNA database entries, in *Proceedings of the AMIA 2000 Annual Symposium,* American Medical Informatics Association, Los Angeles, CA, 4 to 8 November 2000, J. M. Overhage, Ed. (Hanley & Belfus, 2000), pp. 537–541

16. B. Malin, L. Sweeney, How (not) to protect genomic data privacy in a distributed network: Using trail re-identification to evaluate and design anonymity protection systems. *J. Biomed. Inform.* **37**, 179–192 (2004).

17. L. Sweeney, A. Abu, J. Winn, Identifying participants in the personal genome project by name (a re-identification experiment). arXiv:1304.7605 [cs.CY] (29 April 2013).

18. M. Gymrek, A. L. McGuire, D. Golan, E. Halperin, Y. Erlich, Identifying personal genomes by surname inference. *Science* **339**, 321–324 (2013).

19. A. Harmanci, M. Gerstein, Quantification of private information leakage from phenotype-genotype data: Linking attacks. *Nat. Methods* **13**, 251–256 (2016).

20. I. C. Anindya, H. Roy, M. Kantarcioglu, B. Malin, Building a dossier on the cheap: Integrating distributed personal data resources under cost constraints, in *CIKM '17: Proceedings of the 2017 ACM Conference on Information and Knowledge Management*, ACM SIGIR and ACM SIGWEB, Singapore, Singapore, 6 to 10 November 2017 (Association for Computing Machinery, 2017), pp. 1549–1558.

21. C. Lippert, R. Sabatini, M. C. Maher, E. Y. Kang, S. Lee, O. Arikan, A. Harley, A. Bernal, P. Garst, V. Lavrenko, K. Yocum, T. Wong, M. Zhu, W.-Y. Yang, C. Chang, T. Lu, C. W. H. Lee, B. Hicks, S. Ramakrishnan, H. Tang, C. Xie, J. Piper, S. Brewerton, Y. Turpaz, A. Telenti, R. K. Roby, F. J. Och, J. C. Venter, Identification of individuals by trait prediction using whole-genome sequencing data. *Proc. Natl. Acad. Sci. U.S.A.* **114**, 10166–10171 (2017).

22. D. Sero, A. Zaidi, J. Li, J. D. White, T. B. G. Zarzar, M. L. Marazita, S. M. Weinberg, P. Suetens, D. Vandermeulen, J. K. Wagner, M. D. Shriver, P. Claes, Facial recognition from DNA using face-to-DNA classifiers. *Nat. Commun.* **10**, 2557 (2019).

23. The 1000 Genomes Project Consortium, A. Auton, L. D. Brooks, R. M. Durbin, E. P. Garrison, H. M. Kang, J. O. Korbel, J. L. Marchini, S. McCarthy, G. A. McVean, G. R. Abecasis, A global reference for human genetic variation. *Nature* **526**, 68–74 (2015).

24. L. L. Rodriguez, L. D. Brooks, J. H. Greenberg, E. D. Green, The complexities of genomic identifiability. *Science* **339**, 275–276 (2013).

25. Threlkeld, Free databases Ysearch and Mitosearch closing May 24 [Blog] (2018); https://casestone.com/threlkeld/home/latest-news/94-free-databases-ysearch-and-mitosearch-closing-may-24 [accessed 1 October 2021].

26. B. Malin, D. Karp, R. H. Scheuermann, Technical and policy approaches to balancing patient privacy and data sharing in clinical and translational research. *J. Invest. Med.* **58**, 11–18 (2010).

27. US Equal Employment Opportunity Commission, Genetic Information Nondiscrimination Act of 2008; Final Rule. *Fed. Regist.* **81**, 31143–31159 (2016).

28. E. W. Clayton, B. J. Evans, J. W. Hazel, M. A. Rothstein, The law of genetic privacy: Applications, implications, and limitations. *J. Law Biosci.* **6**, 1–36 (2019).

29. B. A. Malin, An evaluation of the current state of genomic data privacy protection technology and a roadmap for the future. *J. Am. Med. Inform. Assoc.* **12**, 28–34 (2005).

30. B. Malin, G. Loukides, K. Benitez, E. W. Clayton, Identifiability in biobanks: Models, measures, and mitigation strategies. *Hum. Genet.* **130**, 383–392 (2011).

31. S. Wang, X. Jiang, S. Singh, R. Marmor, L. Bonomi, D. Fox, M. Dow, L. Ohno-Machado, Genome privacy: Challenges, technical approaches to mitigate risk, and ethical considerations in the United States. *Ann. N. Y. Acad. Sci.* **1387**, 73–83 (2017).

32. B. Berger, H. Cho, Emerging technologies towards enhancing privacy in genomic data sharing. *Genome Biol.* **20**, 128 (2019).

33. D. Grishin, K. Obbad, G. M. Church, Data privacy in the age of personal genomics. *Nat. Biotechnol.* **37**, 1115–1117 (2019).

34. M. M. A. Aziz, M. N. Sadat, D. Alhadidi, S. Wang, X. Jiang, C. L. Brown, N. Mohammed, Privacy-preserving techniques of genomic data—A survey. *Brief. Bioinform.* **20**, 887–895 (2019).

35. A. Mittos, B. Malin, E. de Cristofaro, Systematizing genome privacy research: A privacy-enhancing technologies perspective, in *Proceedings on Privacy Enhancing Technologies*, PETS 2019, Stockholm, Sweden, 16 to 20 July 2019, K. Chatzikokolakis, C. Troncoso, Eds. (Sciendo, 2019), vol. 2019, pp. 87–107.

36. L. Bonomi, Y. Huang, L. Ohno-Machado, Privacy challenges and research opportunities for genomic data sharing. *Nat. Genet.* **52**, 646–654 (2020).

37. L. Sweeney, *k*-anonymity: A model for protecting privacy. *Int. J. Uncertain. Fuzz.* **10**, 557–570 (2002).

38. C. Dwork, Differential privacy, in *Lecture Notes in Computer Science: Proceedings, Part II, of the 33rd International Colloquium on Automata, Languages and Programming*, ICALP 2006, Venice, Italy, 10 to 14 July 2006, M. Bugliesi, B. Preneel, V. Sassone, I. Wegener, Eds. (Springer, 2006), vol. 4052, pp. 1–12.

39. G. Loukides, A. Gkoulalas-Divanis, B. Malin, Anonymization of electronic medical records for validating genome-wide association studies. *Proc. Natl. Acad. Sci. U.S.A.* **107**, 7898–7903 (2010).

40. F. Tramer, Z. Huang, J.-P. Hubaux, E. Ayday, Differential privacy with bounded priors: Reconciling utility and privacy in genome-wide association studies, in *CCS '15: Proceedings of the 22nd ACM Conference on Computer and Communications Security*, ACM SIGSAC, Denver, CO, 12 to 16 October 2015 (Association for Computing Machinery, 2015), pp. 1286–1297.

41. Z. Wan, Y. Vorobeychik, W. Xia, E. W. Clayton, M. Kantarcioglu, R. Ganta, R. Heatherly, B. A. Malin, A game theoretic framework for analyzing re-identification risk. *PLOS ONE* **10**, e0120592 (2015).

42. M. Humbert, E. Ayday, J.-P. Hubaux, A. Telenti, On non-cooperative genomic privacy, in *Lecture Notes in Computer Science: Revised Selected Papers of the 19th International Conference on Financial Cryptography and Data Security*, FC 2015, San Juan, Puerto Rico, 26 to 30 January 2015, R. Böhme, T. Okamoto, Eds. (Springer, 2015), vol. 8975, pp. 407–426.

43. Z. Wan, Y. Vorobeychik, W. Xia, E. W. Clayton, M. Kantarcioglu, B. Malin, Expanding access to large-scale genomic data while promoting privacy: A game theoretic approach. *Am. J. Hum. Genet.* **100**, 316–322 (2017).

44. M. P. Ball, J. R. Bobe, M. F. Chou, T. Clegg, P. W. Estep, J. E. Lunshof, W. Vandewege, A. W. Zaranek, G. M. Church, Harvard personal genome project: Lessons from participatory public research. *Genome Med.* **6**, 10 (2014).

45. J. Watson, *Strategy: An Introduction to Game Theory* (W. W. Norton, ed. 3, 2013).

46. M. Tambe, *Security and Game Theory: Algorithms, Deployed Systems, Lessons Learned* (Cambridge Univ. Press, 2012).

47. W. Xia, M. Kantarcioglu, Z. Wan, R. Heatherly, Y. Vorobeychik, B. Malin, Process-driven data privacy, in *CIKM '15: Proceedings of the 24th ACM International Conference on Information and Knowledge Management*, ACM SIGIR and ACM SIGWEB, Melbourne, Australia, 19 to 23 October 2015 (Association for Computing Machinery, 2015), pp. 1021–1030.

48. A. Acquisti, L. Brandimarte, G. Loewenstein. Privacy and human behavior in the age of information. *Science* **347**, 509–514 (2015).

49. L. Sweeney, Achieving *k*-anonymity privacy protection using generalization and suppression. *Int. J. Uncertain. Fuzz.* **10**, 571–588 (2002).

50. A. Machanavajjhala, D. Kifer, J. Gehrke, M. Venkitasubramaniam. *l*-diversity: Privacy beyond *k*-anonymity. *ACM Trans. Knowl. Discov. Data* **1**, 3-es (2007).

51. N. Li, T. Li, S. Venkatasubramanian, *t*-closeness: Privacy beyond *k*-anonymity and *l*-diversity, in *ICDE '07: Proceeding of the 2007 IEEE 23rd International Conference on Data Engineering*, IEEE Computer Society, Istanbul, Turkey, 15 to 20 April 2007 (Institute of Electrical and Electronics Engineers, 2007), pp. 106–115.

52. R. Wang, Y. F. Li, X. Wang, H. Tang, X. Zhou, Learning your identity and disease from research papers: Information leaks in genome wide association study, in *CCS '09: Proceedings of the 16th ACM Conference on Computer and Communications Security*, ACM SIGSAC, Chicago, IL, 9 to 13 November 2009 (Association for Computing Machinery, 2009), pp. 534–544.

53. D. Garcia, Leaking privacy and shadow profiles in online social networks. *Sci. Adv.* **3**, e1701172 (2017).

54. I. Deznabi, M. Mobayen, N. Jafari, O. Tastan, E. Ayday, An inference attack on genomic data using kinship, complex correlations, and phenotype information. *IEEE/ACM Trans. Comput. Biol. Bioinform.* **15**, 1333–1343 (2018).

55. K. Ayoz, E. Ayday, A. E. Cicek, Genome reconstruction attacks against genomic data-sharing beacons, in *Proceedings on Privacy Enhancing Technologies*, PETS 2021, The Internet, 12 to 16 July 2021, A. Johnson, F. Kerschbaum, Eds. (Sciendo, 2021), vol. 2021, pp. 28–48.

56. M. D. Edge, B. F. B. Algee-Hewitt, T. J. Pemberton, J. Z. Li, N. A. Rosenberg, Linkage disequilibrium matches forensic genetic records to disjoint genomic marker sets. *Proc. Natl. Acad. Sci. U.S.A.* **114**, 5671–5676 (2017).

57. J. Kim, M. D. Edge, B. F. B. Algee-Hewitt, J. Z. Li, N. A. Rosenberg, Statistical detection of relatives typed with disjoint forensic and biomedical loci. *Cell* **175**, 848–858.e6 (2018).

58. F. Prasser, J. Eicher, H. Spengler, R. Bild, K. A. Kuhn, Flexible data anonymization using ARX—Current status and challenges ahead. *Softw. Pract. Exp.* **50**, 1277–1304 (2020).

59. F. Prasser, J. Gaupp, Z. Wan, W. Xia, Y. Vorobeychik, M. Kantarcioglu, K. Kuhn, B. Malin, An open source tool for game theoretic health data de-identification, in *AMIA 2017 Annual Symposium Proceedings*, American Medical Informatics Association, Washington, DC, 4 to 8 November 2017 (American Medical Informatics Association, 2017), pp. 1430–1439.

60. T. Dalenius, Finding a needle in a haystack or identifying anonymous census record. *J. Off. Stat.* **2**, 329–336 (1986).

61. L. Sweeney. Simple demographics often identify people uniquely. (Technical Report LIDAP-WP3, Carnegie Mellon University, 2000); https://dataprivacylab.org/projects/identifiability/paper1.pdf [accessed 1 October 2021].

62. Y. Erlich, T. Shor, I. Pe'er, S. Carmi, Identity inference of genomic data using long-range familial searches. *Science* **362**, 690–694 (2018).

63. P. Ney, L. Ceze, T. Kohno, Genotype extraction and false relative attacks: Security risks to third-party genetic genealogy services beyond identity inference, in *Proceedings of the 2020 Network and Distributed System Security Symposium*, NDSS 2020, San Diego, CA, 23 to 26 February 2020 (Internet Society, 2020); https://dx.doi.org/10.14722/ndss.2020.23049 [accessed 1 October 2021].

64. M. Humbert, K. Huguenin, J. Hugonot, E. Ayday, J.-P. Hubaux, De-anonymizing genomic databases using phenotypic traits, in *Proceedings on Privacy Enhancing Technologies*, PETS 2015, Philadelphia, PA, 30 June to 2 July 2015, A. Kapadia, S. J. Murdoch, Eds. (De Gruyter, 2015), vol. 2015, pp. 99–114.

65. R. Cai, Z. Hao, M. Winslett, X. Xiao, Y. Yang, Z. Zhang, S. Zhou, Deterministic identification of specific individuals from GWAS results. *Bioinformatics* **31**, 1701–1707 (2015).

66. L. Zhang, Q. Pan, Y. Wang, X. Wu, X. Shi, Bayesian network construction and genotype-phenotype inference using gwas statistics. *IEEE/ACM Trans. Comput. Biol. Bioinform.* **16**, 475–489 (2017).

67. B. A. Malin, Protecting genomic sequence anonymity with generalization lattices. *Methods Inf. Med.* **44**, 687–692 (2005).

68. S. Sankararaman, G. Obozinski, M. I. Jordan, E. Halperin, Genomic privacy and limits of individual detection in a pool. *Nat. Genet.* **41**, 965–967 (2009).

69. M. Humbert, E. Ayday, J.-P. Hubaux, A. Telenti, Reconciling utility with privacy in genomics, in *WPES '14: Proceedings of the 13th Workshop on Privacy in the Electronic Society*, ACM SIGSAC, Scottsdale, AZ, 3 November 2014 (Association for Computing Machinery, 2014), pp. 11–20.

70. G. Kale, E. Ayday, O. Tastan, A utility maximizing and privacy preserving approach for protecting kinship in genomic databases. *Bioinformatics* **34**, 181–189 (2018).

71. E. Yilmaz, E. Ayday, T. Ji, P. Li, Preserving genomic privacy via selective sharing, in *WPES '20: Proceedings of the 19th Workshop on Privacy in the Electronic Society*, ACM SIGSAC, Virtual Event, USA, 9 November 2020 (Association for Computing Machinery, 2020), pp. 163–179.

72. S. Simmons, C. Sahinalp, B. Berger, Enabling privacy-preserving GWASs in heterogeneous human populations. *Cell Syst.* **3**, 54–61 (2016).

73. H. Cho, S. Simmons, R. Kim, B. Berger, Privacy-preserving biomedical database queries with optimal privacy-utility trade-offs. *Cell Syst.* **10**, 408–416.e9 (2020).

74. H. Bae, D. Jung, H. Choi, S. Yoon, AnomiGAN: Generative adversarial networks for anonymizing private medical data, in *Biocomputing 2020: Proceedings of the Pacific Symposium*, PSB 2020, Kohala Coast, Hawaii, 3 to 7 January 2020, R. B. Altman, A. K. Dunker, L. Hunter, M. D. Ritchie, T. Murray, T. E. Klein, Eds. (World Scientific, 2020), pp. 563–574.

75. B. Yelmen, A. Decelle, L. Ongaro, D. Marnetto, C. Tallec, F. Montinaro, C. Furtlehner, L. Pagani, F. Jay, Creating artificial human genomes using generative neural networks. *PLOS Genet.* **17**, e1009303 (2021).

76. B. K. Beaulieu-Jones, Z. S. Wu, C. Williams, R. Lee, S. P. Bhavnani, J. B. Byrd, C. S. Greene. Privacy-preserving generative deep neural networks support clinical data sharing. *Circ. Cardiovasc. Qual. Outcomes* **12**, e005122 (2019).

77. E. Halperin, D. A. Stephan, SNP imputation in association studies. *Nat. Biotechnol.* **27**, 349–351 (2009).

78. J. Marchini, B. Howie, Genotype imputation for genome-wide association studies. *Nat. Rev. Genet.* **11**, 499–511 (2010).

79. J. Kaye, L. Curren, N. Anderson, K. Edwards, S. M. Fullerton, N. Kanellopoulou, D. Lund, D. G. MacArthur, D. Mascalzoni, J. Shepherd, P. L. Taylor, S. F. Terry, S. F. Winter, From patients to partners: Participant-centric initiatives in biomedical research. *Nat. Rev. Genet.* **13**, 371–376 (2012).

80. E. W. Clayton, C. M. Halverson, N. A. Sathe, B. A. Malin, A systematic literature review of individuals' perspectives on privacy and genetic information in the United States. *PLOS ONE* **13**, e0204417 (2018).

81. A. L. McGuire, J. M. Oliver, M. J. Slashinski, J. L. Graves, T. Wang, P. Adam Kelly, W. Fisher, C. C. Lau, J. Goss, M. Okcu, D. Treadwell-Deering, A. M. Goldman, J. L. Noebels, S. G. Hilsenbeck, To share or not to share: A randomized trial of consent for data sharing in genome research. *Genet. Med.* **13**, 948–955 (2011).

82. J. M. Oliver, M. J. Slashinski, T. Wang, P. A. Kelly, S. G. Hilsenbeck, A. L. McGuire, Balancing the risks and benefits of genomic data sharing: Genome research participants' perspectives. *Public Health Genomics* **15**, 106–114 (2012).

83. D. Deuber, C. Egger, K. Fech, G. Malavolta, D. Schroder, S. A. K. Thyagarajan, F. Battke, C. Durand, My genome belongs to me: Controlling third party computation on genomic data, in *Proceedings on Privacy Enhancing Technologies*, PETS 2019, Stockholm, Sweden, 16 to 20 July 2019, K. Chatzikokolakis, C. Troncoso, Eds. (Sciendo, 2019), vol. 2019, pp. 108–132.

84. H. I. Ozercan, A. M. Ileri, E. Ayday, C. Alkan, Realizing the potential of blockchain technologies in genomics. *Genome Res.* **28**, 1255–1263 (2018).

85. K. Gammon, Experimenting with blockchain: Can one technology boost both data integrity and patients' pocketbooks? *Nat. Med.* **24**, 378–381 (2018).

86. D. Grishin, J. L. Raisaro, J. R. Troncoso-Pastoriza, K. Obbad, K. Quinn, M. Misbach, J. Gollhardt, J. Sa, J. Fellay, G. M. Church, J.-P. Hubaux, Citizen-centered, auditable and privacy-preserving population genomics. *Nat. Comput. Sci.* **1**: 192–198 (2021).

87. W. Xia, R. Heatherly, X. Ding, J. Li, B. A. Malin, R-U policy frontiers for health data de-identification. *J. Am. Med. Inform. Assoc.* **22**, 1029–1041 (2015).

88. M. Humbert, E. Ayday, J.-P. Hubaux, A. Telenti, Addressing the concerns of the lacks family: Quantification of kin genomic privacy, in *CCS '13: Proceedings of the 2013 ACM SIGSAC Conference on Computer and Communications Security*, ACM SIGSAC, Berlin, Germany, 4 to 8 November 2013 (Association for Computing Machinery, 2015), pp. 1141–1152.

89. M. Humbert, E. Ayday, J.-P. Hubaux, A. Telenti, Quantifying interdependent risks in genomic privacy. *ACM Trans. Priv. Secur.* **20**, 3 (2017).

90. D. W. Craig, R. M. Goor, Z. Wang, J. Paschall, J. Ostell, M. Feolo, S. T. Sherry, T. A. Manolio, Assessing and managing risk when sharing aggregate genetic variant data. *Nat. Rev. Genet.* **12**, 730–736 (2011).

91. Z. Wan, Y. Vorobeychik, M. Kantarcioglu, B. Malin, Controlling the signal: Practical privacy protection of genomic data sharing through beacon services. *BMC Med. Genomics* **10**, 39 (2017).

92. S. S. Shringarpure, C. D. Bustamante, Privacy risks from genomic data-sharing beacons. *Am. J. Hum. Genet.* **97**, 631–646 (2015).

93. I. Wagner, Evaluating the strength of genomic privacy metrics. *ACM Trans. Priv. Secur.* **20**, 2 (2017).

94. M. H. Wooders, Equivalence of games and markets. *Econometrica* **62**, 1141–1160 (1994).

95. Y. Shoham, K. Leyton-Brown, *Multiagent Systems: Algorithmic, Game-Theoretic, and Logical Foundations* (Cambridge Univ. Press, 2008).

96. I. Goodfellow, J. Pouget-Abadie, M. Mirza, B. Xu, D. Warde-Farley, S. Ozair, A. Courville, Y. Bengio, Generative adversarial nets, in *Advances in neural information processing systems (NIPS '14): Proceedings of the 27th International Conference on Neural Information Processing Systems – Volume 2*, NIPS 2014, Montreal, Canada, 8 to 13 December 2014, Z. Ghahramani, M. Welling, C. Cortes, N. D. Lawrence, K. Q. Weinberger, Eds. (MIT Press, 2014), pp. 2672–2680; https://dl.acm.org/doi/10.5555/2969033.2969125 [accessed 1 October 2021].

97. A. Luedtke, M. Carone, N. Simon, O. Sofrygin, Learning to learn from data: Using deep adversarial learning to construct optimal statistical procedures. *Sci. Adv.* **6**, eaaw2140 (2020).

98. R. Nix, M. Kantarcioglu, Incentive compatible privacy-preserving distributed classification. *IEEE Trans. Dependable Secure Comput.* **9**, 451–462 (2012).

99. J. Blocki, N. Christin, A. Datta, A. D. Procaccia, A. Sinha, Audit games, in *IJCAI '13: Proceedings of the Twenty-Third International Joint Conference on Artificial Intelligence*, IJCAI 2013, Beijing, China, 3 to 9 August 2013, F. Rossi, Ed. (AAAI Press, 2013), pp. 41–47; https://dl.acm.org/doi/10.5555/2540128.2540137 [accessed 1 October 2021].

100. M. Li, D. Carrell, J. Aberdeen, L. Hirschman, J. Kirby, B. Li, Y. Vorobeychik, B. A. Malin, Optimizing annotation resources for natural language de-identification via a game theoretic framework. *J. Biomed. Inform.* **61**, 97–109 (2016).

101. R. Shokri, G. Theodorakopoulos, C. Troncoso, Privacy games along location traces: A game theoretic framework for optimizing location privacy. *ACM Trans. Priv. Secur.* **19**, 11 (2016).

102. Z. Wan, Y. Vorobeychik, E. Wright Clayton, M. Kantarcioglu, B. A. Malin, Game theory for privacy-preserving sharing of genomic data, in *Responsible Genomic Data Sharing: Challenges and Approaches*, X. Jiang, H. Tang, Eds. (Academic Press, 2020), pp. 135–160.

103. L. Sweeney, "Computational disclosure control: A primer on data privacy protection," thesis, Massachusetts Institute of Technology, Cambridge, MA (2001).

104. A. Gionis, T. Tassa, *k*-Anonymization with minimal loss of information. *IEEE Trans. Knowl. Data Eng.* **21**, 206–219 (2008).

105. J. Branson, N. Good, J. Chen, W. Monge, C. Probst, K. El Emam, Evaluating the re-identification risk of a clinical study report anonymized under EMA Policy 0070 and Health Canada Regulations. *Trials* **21**, 200 (2020).

106. C. Sudlow, J. Gallacher, N. Allen, V. Beral, P. Burton, J. Danesh, P. Downey, P. Elliott, J. Green, M. Landray, B. Liu, P. Matthews, G. Ong, J. Pell, A. Silman, A. Young, T. Sprosen, T. Peakman, R. Collins, UK biobank: An open access resource for identifying the causes of a wide range of complex diseases of middle and old age. *PLOS Med.* **12**, e1001779 (2015).

107. M. Shabani, Blockchain-based platforms for genomic data sharing: A de-centralized approach in response to the governance problems? *J. Am. Med. Inform. Assoc.* **26**, 76–80 (2019).

108. A. Artyushina, The EU is launching a market for personal data. Here's what that means for privacy, in *MIT Technology Review* (11 August 2020); www.technologyreview.com/2020/08/11/1006555/eu-data-trust-trusts-project-privacy-policy-opinion [accessed 1 October 2021].

109. F. K. Dankar, K. El Emam, A method for evaluating marketer re-identification risk, in *EDBT '10: Proceedings of the 2010 EDBT/ICDT Workshops*, EDBT/ICDT 2010, Lausanne, Switzerland, 22 to 26 March 2010, F. Daniel, L. Delcambre, F. Fotouhi, I. Garrigós, G. Guerrini, J.-N. Mazón, M. Mesiti, S. Müller-Feuerstein, J. Trujillo, T. M. Truta, B. Volz, E. Waller, L. Xiong, E. Zimányi, Eds. (Association for Computing Machinery, 2010), pp. 28.

110. B. Peng, M. Kimmel, simuPOP: A forward-time population genetics simulation environment. *Bioinformatics* **21**, 3686–3687 (2005).

111. B. Peng, C. I. Amos, Forward-time simulations of non-random mating populations using simuPOP. *Bioinformatics* **24**, 1408–1409 (2008).

112. J. Comenetz, Frequently occurring surnames in the 2010 census. US Census Bureau (2016); www.census.gov/topics/population/genealogy/data/2010_surnames.html [accessed 1 October 2021].

113. US Census Bureau, Population, population change, and estimated components of population change: April 1, 2010 to July 1, 2019 (NST-EST2019-alldata). US Census Bureau (2019); www.census.gov/data/tables/time-series/demo/popest/2010s-state-total.html [accessed 1 October 2021].

114. P. Sanchez-Diz, C. Alves, E. Carvalho, M. Carvalho, R. Espinheira, O. Garcia, M. F. Pinheiro, L. Pontes, M. J. Porto, O. Santapa, C. Silva, D. Sumita, S. Valente, M. Whittle, I. Yurrebaso, A. Carracedo, A. Amorim, L. Gusmão; GEP-ISFG (The Spanish and Portuguese Working Group of the International Society for Forensic Genetics), Population and segregation data on 17 Y-STRs: Results of a GEP-ISFG collaborative study. *Int. J. Legal Med.* **122**, 529–533 (2008).

115. US Census Bureau, Source of income in 2015- people 15 years old and over, by income of specified type in 2015, age, race, Hispanic origin, and sex, in *Current Population Survey, 2016 Annual Social and Economic Supplement*, US Census Bureau (2016); www2.census.gov/programs-surveys/cps/tables/pinc-08/2016/pinc08_1_1_1.xls [accessed 1 October 2021].

116. A. Sraders, What is the middle class? Income and range. *TheStreet*, 21 January 2019 [updated 2 April 2020]; www.thestreet.com/personal-finance/what-is-middle-class-14833259 [accessed 1 October 2021].

117. M. Kimura, T. Ohta, Stepwise mutation model and distribution of allelic frequencies in a finite population. *Proc. Natl. Acad. Sci. U.S.A.* **75**, 2868–2872 (1978).

118. J. A. Martin, B. E. Hamilton, M. J. K. Osterman, A. K. Driscoll, Births: Final data for 2018 [Table 3]. *Natl. Vital Stat. Rep.* **68**, 13 (2019); www.cdc.gov/nchs/data/nvsr/nvsr68/nvsr68_13-508.pdf [accessed 1 October 2021].

119. K. El Emam, Seven ways to evaluate the utility of synthetic data. *IEEE Secur. Priv.* **18**, 56–59 (2020).
